# Salicylic acid modulates its catabolic enzymes via proteasomal degradation linked to SCF-associated proximity networks

**DOI:** 10.1038/s41467-026-72241-x

**Published:** 2026-04-20

**Authors:** Natalie Hamada, Malathy Palayam, Jacob Moe-Lange, Gabrielle Wyatt, Christian Montes, Sun Hyun Chang, Annie Hu, Savithramma P. Dinesh-Kumar, Philipp Zerbe, Justin W. Walley, Nitzan Shabek

**Affiliations:** 1 Department of Plant Biology, College of Biological Sciences, University of California, Davis, Davis, CA USA; 2 Department of Plant Pathology, Entomology and Microbiology, College of Agriculture and Life Sciences, Iowa State University, Ames, IA USA; 3 The Genome Center, College of Biological Sciences, University of California, Davis, Davis, CA USA; 4 Innovative Genomics Institute, University of California, Berkeley, CA USA

**Keywords:** Enzymes, Plant signalling, Proteomic analysis, Proteasome

## Abstract

Salicylic acid (SA) is a central regulator of plant immunity, and precise control of its levels is essential to balance defense and growth. However, the mechanisms controlling the stability and abundance of SA-catabolizing enzymes remain elusive. Here we show that the SA hydroxylases DOWNY MILDEW RESISTANT 6 (DMR6) and DMR6-LIKE OXYGENASE 1 (DLO1) are targeted for ubiquitin–proteasome-dependent degradation. SA promotes DMR6 turnover but stabilizes DLO1, linking catalytic activity and conformational dynamics to protein fate. Structural and biochemical analyses indicate that SA binding induces conformational changes in DMR6, particularly in a conserved C-terminal helix, which may contribute to its susceptibility to degradation. Proximity labeling of DMR6 and DLO1 identified a previously uncharacterized Kelch-type F-box protein, which we designate as DMR6-ASSOCIATED F-BOX 1 (DAF1), that contributes to SCF-type E3 ligase-mediated proteasomal turnover of DMR6 in planta, thereby modulating SA-mediated cell death. Complementary proximity labeling of the SCF adaptor ASK1 in Arabidopsis during *Pseudomonas syringae* infection uncovered remodeling of F-box networks while consistently recovering DMR6 and DLO1, highlighting their integration within immune-responsive proteolytic circuits. These findings support a self-limiting regulatory circuit in which SA simultaneously induces and destabilizes its catabolic enzymes, coupling hormone metabolism with proteasome-mediated control of immune homeostasis.

## Abstract (web summary)

Salicylic acid (SA) controls plant immunity but requires tight regulation. Here, the authors show that SA modulates its own inactivation by controlling the stability of its catabolic enzymes through cellular protein degradation pathways, revealing a feedback mechanism that balances defense and growth.

## Introduction

Protein degradation by the ubiquitin-proteasome system (UPS) plays a crucial role in maintaining proteome homeostasis and regulating signal transduction in plants^1,2^. Central to this system are E3 ubiquitin ligases, which confer substrate specificity and direct the polyubiquitination of target proteins, marking them for degradation by the 26S proteasome. Among these, SKP1-Cul1-F-box (SCF)-type E3 ligases serve as major regulatory hubs in hormone signaling, controlling processes, such as auxin, jasmonic acid (JA), gibberellin, strigolactone, abscisic acid (ABA), and cytokinin responses^1,3^. Their role in degrading transcriptional repressors or pathway intermediates is well established; however, the contribution of the UPS to hormone catabolism, particularly the turnover of metabolic enzymes, remains poorly understood.

Salicylic acid (SA) is a key hormone that accumulates rapidly upon pathogen attack to activate local and systemic resistance^4^. Tight control of SA levels is essential, as excessive accumulation leads to growth inhibition and autoimmunity, while insufficient SA impairs defense^4,5^. In Arabidopsis, SA catabolism is mediated by two 2-oxoglutarate Fe(II)-dependent dioxygenases (2OGDDs), downy mildew resistant 6 (DMR6) and its paralog DMR6-like oxygenase 1 (DLO1)^6,7^. These enzymes hydroxylate SA to form inactive metabolites—DMR6 generates 2,5-dihydroxybenzoic acid (2,5-DHBA), and DLO1 catalyzes the formation of 2,3- and 2,5-DHBA—thus acting as negative regulators of immunity^6,7^. Loss-of-function mutations in these genes elevate SA levels and enhance pathogen resistance, while overexpression promotes susceptibility^6,8,9^. Notably, DMR6 appears to play a more prominent role in immunity than DLO1, due to its stronger transcriptional induction during infection and its distinct localization at infection sites^9^.

*DMR6* and *DLO1* expression is upregulated in response to infection by *Hyaloperonospora arabidopsidis, Erysiphe orontii*, and *Pseudomonas syringae*, as well as by SA itself, forming a canonical negative feedback loop^6,8,9^. *DMR6* is expressed in tissues directly adjacent to the pathogen entry site, while *DLO1* is localized to vascular regions, suggesting spatial partitioning in SA inactivation^9^. Although these expression dynamics have been characterized, the mechanisms regulating the stability of DMR6 and DLO1 proteins remain largely unknown.

Our recent proximity-labeling analysis of ASK1 (Arabidopsis SKP1 homolog) and Cul1, both tagged with TurboID, identified DMR6 and DLO1 among candidate SCF complex substrates, suggesting a potential link between SA catabolism and SCF-mediated protein degradation^10^. Overexpression of tagged DMR6 and DLO1 in *Nicotiana benthamiana* leaves can co-immunoprecipitate tagged ubiquitin, showing a characteristic polyubiquitination pattern. Moreover, a recent proteomic study further detected di-glycine (ubiquitin remnant) modifications on DMR6 and DLO1, providing orthogonal evidence that these enzymes are bona fide ubiquitination targets^10,11^. Given that other 2OGDD enzymes involved in hormone metabolism, such as JA oxidases (JOXs), are degraded in a JA-responsive manner, we hypothesized that SA levels and enzyme activity might influence DMR6/DLO1 turnover via proteasome-mediated pathways^12^.

Here, we show that DMR6 and DLO1 undergo UPS-dependent degradation and that this process is modulated by SA: DMR6 degradation is accelerated, whereas DLO1 degradation is slowed. We combined biochemical, structural, in planta, and proteomic approaches to define the basis of this regulation. Catalytic mutants exhibit altered degradation kinetics, suggesting that enzymatic function influences their stability. Computational and biochemical analyses revealed that SA binding induces conformational rearrangements in DMR6, particularly within a conserved C-terminal helix (CTH). This CTH is less dynamic and less conserved in DLO1, suggesting a structural basis for differential degradation. TurboID-based proximity labeling identified a previously uncharacterized Kelch-type F-box protein as a putative DMR6 and DLO1 interactor, while proximity labeling of the SCF adapter ASK1 in transgenic Arabidopsis lines during *Pseudomonas syringae* pv. Tomato (*Pst* DC3000) infection suggested constitutive SCF-mediated regulation of DMR6 and DLO1. Together, our findings support a hormone-responsive degradation circuit in which SA controls the stability of its own catabolic enzymes. This circuit links ligand perception, catalytic activity, and conformational dynamics to SCF E3 ubiquitin ligase-mediated proteolysis, integrating metabolic feedback into immune regulation.

## Results

### Proteasomal turnover of DMR6 and DLO1 is modulated by SA and catalytic activity

**Fig. 1:**
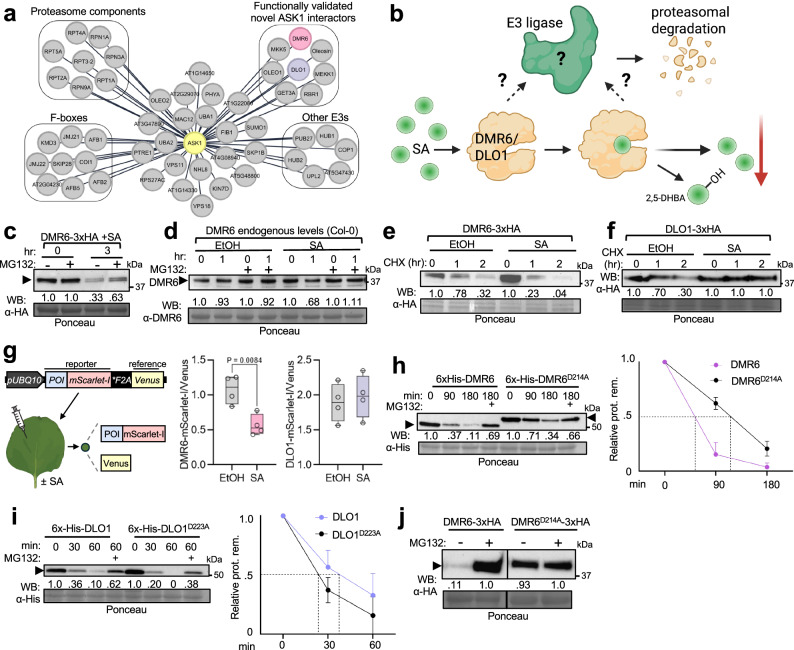
DMR6 and DLO1 proteasomal degradation rates are linked to SA and catalytic activity. **a** A protein interaction network depicts a subset of previously captured ASK1 interactors^10^, highlighting either known or newly linked UPS associations. **b** A summary of the current hypothesis of DMR6/DLO1-mediated SA hydroxylation and proteasomal regulation. *In planta* DMR6 proteasomal degradation during SA treatment in stable pUBQ::DMR6-3xHA (**c**) and WT Col-0 (**d**) plants. Hours indicates the time elapsed since the SA application. **d** Endogenous levels of DMR6 in Col-0 were detected using anti-DMR6 antibody. Endogenous DMR6 abundance is low, and SA-dependent shifts are subtle, consistent with its role as a homeostatic SA-catabolic enzyme subject to both transcriptional induction and proteasomal turnover. DMR6 (**e**) and DLO1 (**f**) undergo proteasomal degradation under SA treatment in *N. benthamiana*. **g** Schematic and ratiometric degradation analysis of DMR6 and DLO1. Left, diagram of the Venus–mScarlet ratiometric reporter used to quantify protein stability. Right, box plot showing representative results of the assay depicting relative fluorescence of DMR6-mScarlet-I-Venus and DLO1 fusion proteins in response to SA or EtOH treatment. Data represent four biological replicates (*n* = 4). Box plots show the median (center line), interquartile range (box, 25th to 75th percentile), and minimum to maximum values (whiskers). All individual data points are shown. The catalytic mutants DMR6^D214A^ (**h**) and DLO1^D223A^ (**i**) exhibit proteasomal turnover relative to the wild-type in vitro, as determined in three independent experiments. Data are depicted as mean ± SEM. **j** Western blot depicting representative *in planta* proteasome inhibition assay of DMR6 and DMR6^D214A^. All samples were resolved by SDS-PAGE and visualized by Western blot using the indicated antibodies. Ponceau staining serves as loading control. All degradation experiments were repeated at least three times. Elements in (**b**, **g**) were created in BioRender. Moe-Lange, J. (2026) BioRender.com/6sequgz.

We previously generated a proximity-dependent protein interaction network for the SCF E3 ligase adapter protein ASK1^10^. Among the ASK1 interactors, a significant subset included known components of the UPS, such as F-box proteins, proteasome subunits, and other UPS regulators (Fig. 1a)^10^. Validation of eight ASK1 interactors from this dataset via *in planta* co-immunoprecipitation (Co-IP) and bimolecular fluorescence complementation identified DMR6 and DLO1 as bona fide ASK1-associated proteins (Fig. 1a)^10^. These findings suggested that SCF-type E3 ligases could regulate DMR6 and DLO1 through proteasomal degradation. Given their role in SA hydroxylation and immune regulation, we hypothesized that the enzymatic activity of DMR6 and DLO1 could impact their susceptibility to proteasomal degradation (Fig. 1b). To gain initial insight into factors affecting DMR6 and DLO1 turnover, we generated stable Col-0 Arabidopsis lines expressing DMR6 fused to 3xHA under the control of ubiquitin promoter (hereafter DMR6-OX). The expression of DMR6-3xHA was confirmed by immunoblotting with HA antibodies (Supplementary Fig. 1a). We examined the stability of DMR6 in DMR6-OX lines following SA treatment. Our results showed that DMR6 protein levels declined after treatment with SA for 3 h, and the addition of the proteasome inhibitor MG132 inhibited DMR6 degradation (Fig. 1c), indicating that SA promotes proteasomal degradation of DMR6 *in planta*. In this and subsequent degradation assays, MG132 consistently stabilized DMR6 but did not completely prevent its degradation. This pattern likely reflects a combination of factors, including secondary proteolytic routes, variable MG132 peptide stability, and differential uptake of the inhibitor by plant tissues^13–18^. To determine whether endogenous DMR6 in wild-type Col-0 is also regulated during SA treatment, we generated a specific anti-DMR6 antibody to monitor DMR6 expressed under its native promoter (see “Methods” and Supplementary Fig. 1b, c). DMR6 protein abundance modestly decreased one hour after SA treatment, and this reduction was minimized by MG132, although degradation was not completely prevented (Fig. 1d). The effect on endogenous levels was expectedly less pronounced than in overexpression lines, consistent with simultaneous SA-induced transcriptional upregulation of DMR6^6^, which can partially offset proteasome-dependent turnover. To assess whether DMR6 and DLO1 exhibit distinct SA responses, we performed transient expression and cycloheximide (CHX) chase assays in *N. benthamiana*. SA treatment accelerated DMR6 degradation compared to the control ethanol (EtOH) treatment (Fig. 1e). However, SA treatment stabilized DLO1 relative to EtOH-treated control (Fig. 1f). These results indicate opposing effects of SA on the turnover of DMR6 and DLO1 paralogs. To further validate and quantify DMR6 protein dynamics with higher precision, we implemented an independent detection method that complements the *in planta* degradation assays. Endogenous DMR6 protein levels are naturally low in Arabidopsis leaves, so SA-dependent changes are therefore small in magnitude. This reflects the physiological regulation of DMR6 in plants, in which SA both induces DMR6 transcription and promotes proteasomal degradation. As a result, the steady-state differences are subtle and difficult to detect by immunoblotting. The relative band-intensity values shown below the blot highlight this low-amplitude pattern. Due to the limited dynamic range of endogenous protein detection, we use the ratiometric mScarlet-I-Venus degradation assay (Fig. 1g) to get a more sensitive and quantitative understanding of the SA-dependent increase in DMR6 turnover. Specifically, we established a ratiometric fluorescent reporter system, in which DMR6-mScarlet-I-Venus and DLO1 fusions were transiently expressed in *N. benthamiana* and monitored following SA or EtOH treatment. SA treatment reduced DMR6 fluorescence. In contrast, EtOH-treated controls showed minimal change, indicating that SA promotes DMR6 degradation (Fig. 1g). In contrast, SA had a negligible effect on DLO1 stability in this assay, consistent with the differential behavior observed in planta.

To complement the ratiometric and in planta analyses with a biochemical approach, we also performed a cell-free degradation assay using recombinant DMR6 and DLO1 proteins incubated with wild-type Arabidopsis extracts (Supplementary Fig. 2a, b). This assay recapitulated proteasome-dependent turnover for both enzymes and revealed that DLO1 was stabilized in the presence of SA and MG132, whereas DMR6 remained rapidly degraded. The magnitude of SA responsiveness was lower for DMR6 than in planta, likely reflecting the absence of subcellular compartmentalization and residual endogenous SA in the extract. Accordingly, we interpret the cell-free degradation assay as qualitative support for SA-enhanced turnover rather than as a quantitative comparison. Importantly, these altered degradation rates were not observed when samples were treated with the SA hydroxylation product, 2,5-dihydroxybenzoic acid (2,5-DHBA), or a solvent alone control (Supplementary Fig. 3a, b), indicating that the observed effects are specific to SA.

In all cell-free assays, MG132 treatment stabilized DMR6 and DLO1, confirming that proteasome-mediated turnover is a major pathway regulating their stability. To investigate the contribution of enzymatic activity to protein stability, we generated catalytically inactive mutants of DMR6 and DLO1 (Supplementary Fig. 3c, d). As members of the 2-oxoglutarate/Fe(II)-dependent dioxygenase (2-ODD) family, both enzymes require an Fe(II) cofactor coordinated by a conserved HX(D/E)X_n_H motif to facilitate oxygen binding and substrate hydroxylation^19^. Following approaches previously applied to DMR6^9,20^, we substituted the conserved aspartic acid (D) residue within this triad with alanine (A) to create the catalytic mutants DMR6^D214A^ and DLO1^D223A^. In cell-free degradation assays, purified DMR6^D214A^ protein exhibited a longer half-life of approximately 120 min compared to ~85 min for wild-type DMR6 (Fig. 1h), while DLO1^D223A^ degraded more rapidly than wild-type DLO1, with an in vitro half-life of approximately 35 min versus ~20 min for wild-type DLO1 (Fig. 1i). In *N. benthamiana* transient expression experiments, CHX chase assays similarly showed that proteasome inhibition with MG132 stabilized wild-type DMR6 over the mutant (Fig. 1j) and that DMR6^D214A^ turns over at a modestly increased rate relative to wild-type *in planta* (Supplementary Fig. 3e). In the transient assay, the difference in turnover between wild-type DMR6 and the catalytic mutant was modest, as expected in this lower-dynamic-range system. We present this result in Supplementary Fig. 3e and highlight the clearer, more reproducible turnover differences seen in the cell-free degradation assay and in the MG132 stabilization experiment (Fig. 1h–j). These results collectively demonstrate that catalytic activity promotes DMR6 proteasomal degradation, while suggesting distinct turnover dynamics for DLO1. Given that DMR6 plays a more prominent role than DLO1 in regulating immune responses, and that DMR6 turnover was more responsive to catalytic activity status in both in vitro and *in planta* assays, we focused subsequent mechanistic investigations on DMR6.

### Differential SA-induced structural remodeling of DMR6 and DLO1 correlates with their proteasomal stability

**Fig. 2:**
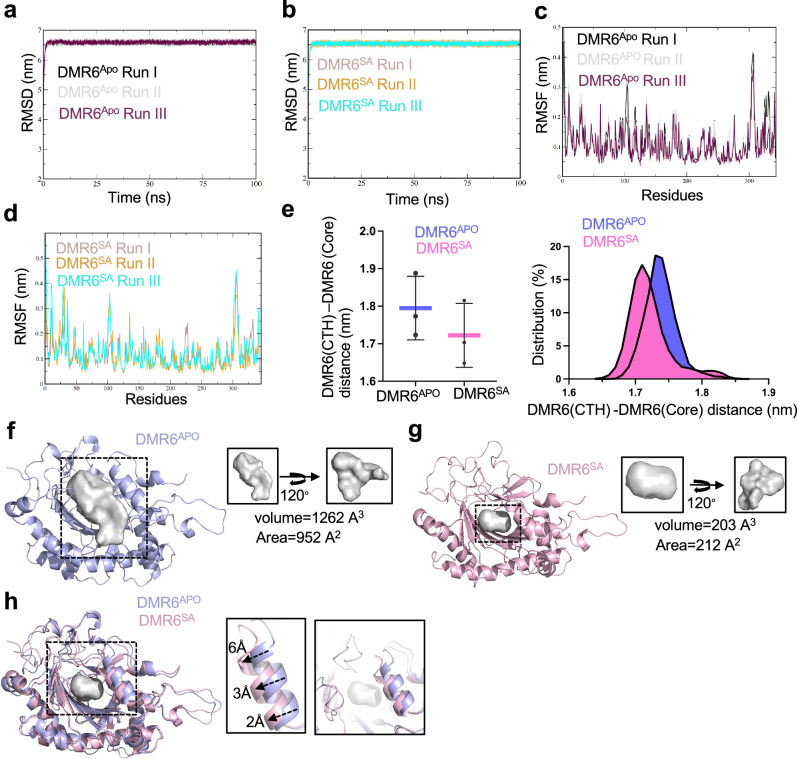
Molecular dynamics analysis of DMR6^apo^ and DMR6^SA^ bound states. **a**, **b** RMSD profiles for DMR6^apo^ and DMR6^SA^ from three independent 100-ns MD trajectories (run I,-brown, run II-gray, and run III-maroon), showing rapid equilibration and stable backbone conformations across replicates. **c**, **d** RMSF plots for DMR6^apo^ and DMR6^SA^ ((run I,-brown, run II-gray and run III-maroon) showing similar residue-level flexibility except for increased fluctuations within the C-terminal helix (CTH; residues T316–S326). **e** The distance between DMR6^CTH(316–326)^ and DMR6^Core(1–315)^ in the apo and salicylic acid (SA) bound states. (Left) Points represent the mean distance from each of three independent molecular dynamics simulations per condition (*n* = 3 independent simulations), with horizontal bars indicating the mean and error bars representing ± SD, showing a shift toward shorter distances in the SA-bound state relative to the apo state, consistent with ligand-induced closure of the catalytic pocket. (Right) Frequency distribution of distances calculated from frames sampled from the equilibrated portions of the trajectories. Independent simulations were initiated with different velocity seeds and analyzed separately without pooling prior to statistical representation. **f**, **g** Representative snapshots of DMR6^apo^ (left, lavender) and DMR6^SA^ (right, pink) highlighting pocket closure and reduction in cavity surface area (from 952 to 212 Å²) and volume (from 1262 to 203 Å³) measured using KVFinder. Volume highlighted in box represents the 120° rotational views illustrating the reduced catalytic pocket volume in the SA-bound state. **h** Structural superposition of DMR6^apo^ and DMR6^SA^ depicting a 3–6 Å inward shift of the CTH upon SA binding, consistent with ligand-induced closure of the catalytic cavity.

Given that SA influences the proteasomal turnover of DMR6 and DLO1, and that catalytic activity modulates DMR6 stability, we next investigated whether SA binding induces conformational changes that could affect susceptibility to proteasomal degradation. Although both enzymes bind SA, they exhibit distinct kinetic behavior: DMR6 has a higher affinity for SA (Km = 5.15 ± 1.44 μM) compared to DLO1 (Km =  58.29 ± 5.27 μM) and uniquely displays substrate inhibition at elevated SA concentrations^6,7^. These differences suggest that SA interaction might trigger distinct structural responses in DMR6 and DLO1, potentially affecting their proteasomal regulation, as structural remodeling is a known mechanism for modulating substrate recognition by E3 ubiquitin ligases^21–24^. To elucidate how SA binding affects the structural dynamics of DMR6 and DLO1, we performed 100 ns all-atom molecular dynamics (MD) simulations for both enzymes in apo and SA-bound states across three independent replicates (hereafter referred to as DMR6^apo^/DMR6^SA^ and DLO1^apo^/DLO1^SA^) (Supplementary Movies 1–4). Analysis of the root-mean-square deviation (RMSD) plotted as mean ± SD across the three independent trajectories showed that both DMR6^apo^ and DMR6^SA^ equilibrated rapidly within the first 10 ns and maintained nearly identical RMSD profiles throughout the simulations. (Supplementary Fig. 4a, b, and Fig. 2a, b). The low standard deviations across replicates confirmed the reproducibility and overall structural stability of both systems. (Supplementary Fig. 4a, b, and Fig. 2a, b). Next, we assessed residue-specific flexibility by calculating the root mean square fluctuation (RMSF). Both DMR6^apo^ and DMR6^SA^ exhibited similar fluctuation patterns across most residues, with overall flexibility of 0.1–0.3 nm (Supplementary Fig. 4c, d, and Fig. 2c, d). However, both states (DMR6^apo^ and DMR6^SA^) displayed slightly increased fluctuations in the region spanning residues 300–330, corresponding to the C-terminal helix (CTH, residues T316–S326) of DMR6 (Supplementary Fig. 4c, d, and Fig. 2c, d). Closer examination of the trajectories indicated that this helix adopted an open and flexible conformation in DMR6^apo^ (Fig. 2e, f, and Supplementary Fig. 4e) and upon SA binding, the CTH in DMR6^SA^ transitioned to a closed conformation, covering the catalytic cavity (Fig. 2e, g, and Supplementary Fig. 4f). To quantitatively evaluate this conformational difference, we measured the distance between the CTH and the protein core (CTH-core distance) across all three replicates. The DMR6^apo^ form consistently exhibited a larger average distance, representing an open catalytic pocket (Fig. 2e, f, h, and Supplementary Fig. 4e), whereas the DMR6^SA^ form showed a ligand-induced inward shift of the CTH, forming a compact, closed conformation. (Fig. 2e, g, h, and Supplementary Fig. 4f). Collectively, these analyses show that while SA does not alter overall DMR6 stability, it drives a localized closure of the catalytic pocket. Furthermore, hydrogen-bond and metal-coordination analyses in DMR6^SA^ revealed that D223 and H212 of DMR6 form stable interactions with both SA and the catalytic Fe^3+^ ion. Specifically, the carboxylate oxygen atoms (OD1 and OD2) of D223 maintained tight coordination to Fe^3+^ while simultaneously forming hydrogen bonds with the oxygens (O1 and O2) of SA. Additionally, the NE2 atom of H212 consistently interacted with oxygen (O2) of SA (Supplementary Fig. 4g). These stable interactions indicate a well-defined coordination geometry, supporting a structurally stable active-site configuration. To further assess the stability of SA within the catalytic pocket, the ligand RMSD analyses were performed across triplicate. The ligand RMSD of DMR6^SA^ remained low and stable (0.02 nm) throughout simulation with minimal variation among replicates (Supplementary Fig. 4h). This tight clustering indicates that SA maintained a stable binding pose within the active site of DMR6.

Similar to DMR6, DLO1 exhibited stable RMSD and RMSF profiles in both states, indicating a well-equilibrated system across replicates (Supplementary Fig. 4i–l). The CTH of DLO1 (F323–N332) exhibited the opposite trend across all three replicates (Supplementary Fig. 5a). The DLO1^apo^ form maintained a closed conformation with the CTH positioned near the catalytic core, whereas DLO1^SA^ displayed a ligand-induced outward shift, adopting an open conformation that exposed the active site (Supplementary Figs. 5a–f). The average CTH-core distance was consistently larger in the SA-bound state (Supplementary Fig. 5a, c, d,f), confirming that SA binding promotes CTH opening, opposite to the closure observed in DMR6. Hydrogen bond and Fe co-ordination analyses revealed that D214 and H212 play a central role in stabilizing the SA-bound state of DLO1^SA^. Specifically, the carboxylate oxygen (OD1 and OD2) of D214 formed bifurcated hydrogen bonds with the oxygen (O2) of SA while simultaneously maintaining coordination to Fe^3+^. In addition, the NE2 atom of H212 forms a stable hydrogen bond with the oxygen (O3) of SA. These interactions were characterized by short and stable distances (0.15–0.35 nm) indicating that Fe^3+^ -D-H catalytic triad remains structurally stable during simulation (Supplementary Fig. 5g). Consistent with these findings, ligand RMSD analysis of DLO1^SA^ demonstrated that SA remained stably bound within DLO1 active site with average fluctuations of 0.03 nm across triplicates (Supplementary Fig. 5h). Together, these results indicate that SA binding stabilizes the catalytic pocket of DLO1, preserving a well-coordinated and dynamically resilient active site. Although intermediate states during SA catalysis were not directly captured, the simulations support the notion that SA promotes dynamic conformational transitions in DMR6 distinct from those in DLO1.

**Fig. 3:**
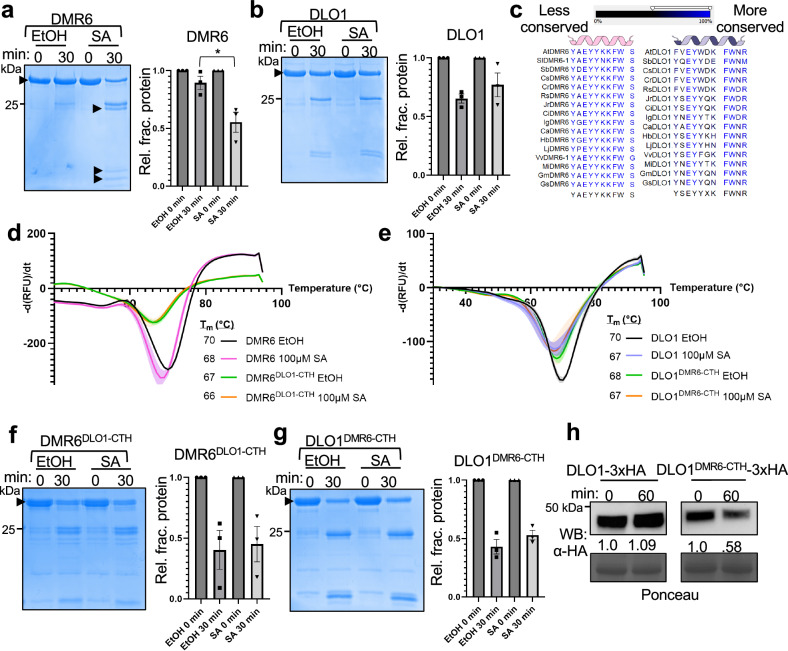
Characterization of DMR6 and DLO1 interaction with SA. **a**, **b** Trypsin-limited proteolysis assay of DMR6 (**a**) and DLO1 (**b**) in the presence of SA or EtOH control. *n* = 3, data are presented as mean ± SEM. In panel (**a**), the single asterisk represents *p* = 0.0316 on an unpaired, two-tailed Student’s *t* test, performed in GraphPad Prism. A representative Coomassie gel is shown for each experiment. **c** Sequence conservation of the DMR6 and DLO1 CTH among selected orthologs. Melting curve representing the average of three technical replicates of DMR6 and DMR6^DLO1-CTH^ (**d**), DLO1 and DLO1^DMR6-CTH^ (**e**) in the presence of SA or EtOH, determined through differential scanning fluorimetry (DSF). Trypsin-limited proteolysis assay of DMR6^DLO1-CTH^ (**f**) and DLO1^DMR6-CTH^ (**g**) in the presence of SA or EtOH. Data are presented as mean ± SEM (*n* = 3). Individual data points are superimposed. The colored error envelopes indicate the standard error across the three technical replicates. **h** Representative Western blot depicting an *in planta* CHX chase assay of DLO1 and DLO1^DMR6-CTH^ in the presence of exogenous SA. Samples were resolved by SDS-PAGE and visualized by Western blot using the indicated antibodies. Ponceau staining serves as loading control. Data are representative of three independent experiments (*n* = 3).

To biochemically characterize these modeled SA-induced changes in structural dynamics, we performed trypsin-limited proteolysis. In this assay, increased protease susceptibility reflects enhanced exposure or flexibility of specific protein regions. Upon SA treatment, DMR6 exhibited accelerated proteolytic cleavage (Fig. 3a), consistent with ligand-induced conformational remodeling. In contrast, DLO1 showed no substantial change in protease sensitivity (Fig. 3b), suggesting that it maintains a relatively static conformation upon SA binding. Sequence conservation analysis revealed that the DMR6 CTH is highly conserved among orthologs, whereas the DLO1 CTH exhibits lower sequence conservation (Fig. 3c and Supplementary Fig. 6a–d). These findings suggest that CTH dynamics and evolutionary conservation are correlated with proteolytic regulation. We then used differential scanning fluorimetry (DSF) to probe ligand-induced changes in protein stability further. Both DMR6 and DLO1 exhibited modest decreases in melting temperature (~1–3 °C) in the presence of SA (Fig. 3d, e), consistent with conformational remodeling upon ligand binding^25^.

To investigate whether the CTH determines these SA-responsive structural changes, we purified chimeric CTH swap mutants, DMR6^DLO1-CTH^ and DLO1^DMR6-CTH^, and subjected them to DSF and trypsin-limited proteolysis. Both chimeras exhibited reduced intrinsic thermal stability compared to their wild-type counterparts and minor SA-induced shifts in Tm (Fig. 3d, e), indicating that the native DMR6 CTH is crucial for ligand-responsive conformational remodeling. In trypsin assays, DMR6^DLO1-CTH^ lost the SA-induced proteolysis sensitivity seen in wild-type DMR6, further implicating the CTH in mediating SA-triggered structural changes (Fig. 3f). As with wild-type DLO1, DLO1^DMR6-CTH^ did not display increased proteolysis in response to SA (Fig. 3g), suggesting that the presence of the DMR6-CTH alone is not sufficient to confer SA-induced conformational remodeling detectable by trypsin-limited proteolysis. However, in planta CHX chase assays revealed that DLO1^DMR6-CTH^ undergoes faster proteasomal turnover than wild-type DLO1 (Fig. 3h), indicating that the CTH swap affects proteasomal turnover. Together, these findings support a role for the DMR6 CTH in mediating proteasomal degradation, while also highlighting that SA-induced conformational responsiveness likely emerges from cooperative contributions beyond the CTH alone.

### Proximity labeling identifies a putative Kelch F-box regulator of DMR6 and DLO1

**Fig. 4:**
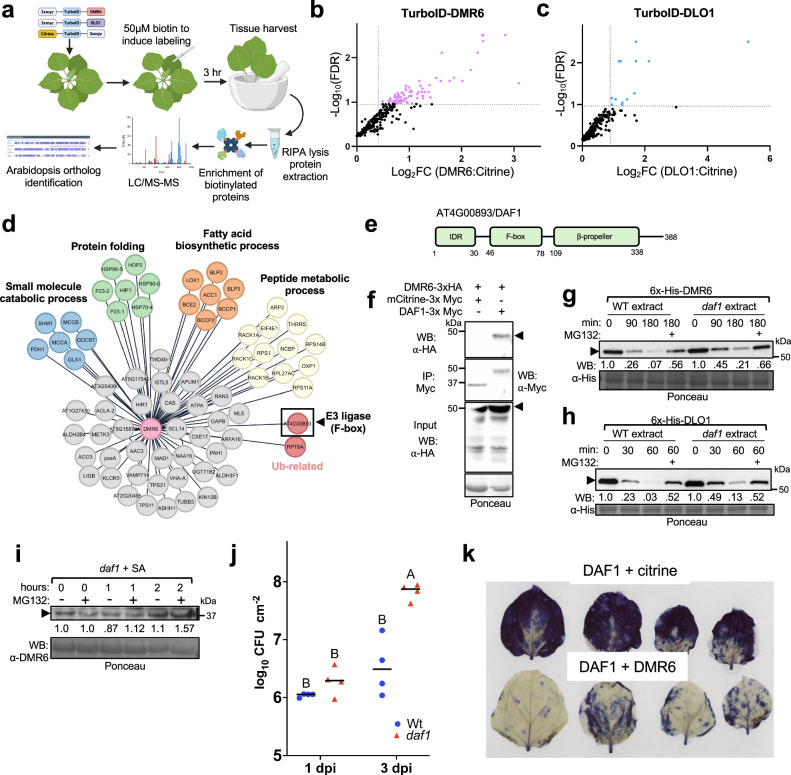
Proximity labeling identifies a potential Kelch F-box regulator of DMR6 and DLO1. **a** Experimental design and workflow for transient expression-based DMR6 and DLO1 proximity labeling (PL) experiments. Volcano plot depicting proteins identified in DMR6 (**b**) and DLO1 (**c**) PL experiments. Colored dots indicate proteins with fold change ≥ 1.7 and *q* ≤ 0.1 compared to citrine control. **d** Protein interaction network for DMR6 based on PL data. **e** Predicted protein functional domains of AT4G00893/Kelch F-box. **f** Western blot depicting Co-IP assay of DMR6-3xHA and DAF1-3xmyc transiently co-expressed in *N. benthamiana*. Citrine-3xmyc served as a negative control. Magnetic myc beads were used to pull citrine-3xmyc and DAF1-3xmyc from native extracts. Extracted protein was separated via SDS-PAGE and coupled to Western blotting with myc and HA antibodies. Ponceau serves as total protein loading control. Representative cell-free degradation assay of recombinant DMR6 (**g**) and DLO1 (**h**) in the presence of WT Col-0 or AT4G00893 (SALK_072882) T-DNA insertion mutant extracts. Assays repeated three times. **i** Representative MG132 chase assay in the AT4G00893 (SALK_072882) mutant during SA treatment. Direct comparison of DMR6 levels in Col-0 and *daf1* is shown separately in Supplementary Fig. 9d because co-exposure on the same membrane bleaches the endogenous DMR6 signal in *daf1* relative to Col-0 and prevents accurate measurement. All samples were resolved by SDS-PAGE and visualized by Western blot using the indicated antibodies. Ponceau staining serves as loading control. Experiments were repeated at least three times. **j** Colony counts for a representative plating assay of *Pst* bacterial recovery assays from Col-0 (WT, blue circles) and *daf1* (red triangles, T-DNA mutant). Data are plotted as mean colony-forming units (CFUs). Bars represent medians. Data were analyzed using the GraphPad Prism 2-way ANOVA test. Assays repeated three times. **k** Representative leaves of *N. benthamiana* plants transiently expressing either DAF1 + citrine or DAF1 + DMR6 (OD_600_ = 0.5 for all) at 2 dpi, following trypan blue staining and overnight clearing in 90% EtOH. Elements in (**a**) were created in BioRender. Moe-Lange, J. (2026) BioRender.com/wjrzdxz.

We previously identified DMR6 and DLO1 among the ASK1-TurboID proxitomes^10^, suggesting that SCF-type E3 ligases may regulate them. While ASK1-based proximity labeling broadly captures SCF-associated networks, it does not resolve the substrate-specific recognition mediated by individual F-box proteins. To explore this further, we performed TurboID-based proximity labeling of DMR6 and DLO1 to identify candidate F-box regulators. Since DMR6 and DLO1 undergo proteasomal degradation in *N. benthamiana* during transient expression and given that these enzymes and their regulatory pathways are broadly conserved across angiosperms, this system provides a suitable context for capturing E3-associated interactions under steady-state conditions. To that end, TurboID fusions of DMR6, DLO1, or citrine (negative control) were transiently expressed in *N. benthamiana*, followed by biotin labeling, streptavidin-based enrichment, TMT labeling, and quantitative proteomic analysis across three biological replicates (Fig. 4a and Supplementary Fig. 7a). A total of 583 and 582 proteins were quantified in the DMR6 and DLO1 datasets, respectively (Supplementary Data 1). Of these, 79 (DMR6) and 13 (DLO1) were enriched and designated as candidate interactors (Fig. 4b–d and Supplementary Data 1). Among these, nine proteins were shared between DMR6 and DLO1, including HOP2, a tetratricopeptide-repeat (TPR) protein previously linked to F-box protein accumulation^26,27^, (Supplementary Fig. 7b and Supplementary Data 1). Other enriched proteins included NAA15, CXE17, and OXP1, enzymes associated with cytoplasmic metabolism and stress response^28–30^ (Fig. 3d, Supplementary Fig. 7c, and Supplementary Data 1). These results suggest that DMR6 and DLO1 may interact with a conserved subset of metabolic and regulatory proteins. Although no E3 ligases passed our enrichment threshold, one Kelch-domain F-box candidate, NbD015204.1, was identified with modest enrichment in both DMR6 and DLO1 datasets (linear fold enrichment ~1.1–1.2) (Supplementary Data 1). Given that DMR6 is broadly conserved among land plants, we next focused on characterizing this potential F-box regulator. The *Arabidopsis thaliana* ortholog AT4G00893 encodes a previously uncharacterized Kelch-repeat F-box (KFB) protein identified in our DMR6–TurboID interactome. We hereafter refer to this F-box as DMR6-associated F-box 1 (DAF1). F-box proteins are typically expressed at low levels and can form transient interactions with substrates, often leading to modest enrichment values in proximity labeling datasets^31,32^. Similar low fold-change values have been observed for biologically relevant F-box proteins in prior TurboID studies^10^. Based on its domain architecture, evolutionary conservation, and reproducible co-enrichment with both DMR6 and DLO1, DAF1 was selected for further characterization as a candidate E3 component—reports of other KFBs in hormone signaling further support its potential regulatory role^33–35^. Furthermore, following transcriptomic analyses of publicly available datasets, we observed that *DAF1* is modestly upregulated in roots in response to treatment with the bacterial peptide flg22, suggesting a possible role in immune signaling. Further transcriptomic data on Col-0, *coi1-1*, and *npr1-1* infected with various isolates of the fungal pathogen *Botrytis cinerea* show that *DAF1* may undergo differential transcriptional regulation in a genotype- and *B. cinerea* isolate-dependent manner, further suggesting an association between *DAF1* and defense hormone signaling^36^. Protein sequence analysis revealed an N-terminal intrinsically disordered region (residues 1–30), a canonical F-box motif (residues 46–78), and a C-terminal Kelch β-propeller (residues 109–338) (Fig. 4e). Orthologs were identified across diverse land plants, with conserved Kelch domains and variable IDRs, suggesting conserved substrate recognition with lineage-specific regulatory adaptation (Supplementary Fig. 8).

### DMR6 and DAF1 interact and may co-regulate cell death and immunity

We next characterized the biological significance of DAF1 and focused on its potential regulatory effects on DMR6 *in planta*. To determine whether DAF1 physically associates with DMR6, we co-expressed 3×myc–DAF1 and 3×HA–DMR6 in *N. benthamiana*. Co-immunoprecipitation confirmed that DAF1 pulls down DMR6 from native extracts, indicating their physical association in planta (Fig. 4f). We additionally performed AlphaFold3-based complex modeling and protein–protein docking of DAF1 assembled within the SCF complex together with SA-bound DMR6, which revealed a high-confidence configuration positioning the DMR6 C-terminal region near the DAF1 Kelch domain and oriented toward the cullin-E2-Ub module, consistent with a substrate poised for ubiquitination (Supplementary Fig. 9a). However, simulations using the CTH alone did not reproduce the whole interface, implying that additional surrounding residues contribute to stable binding. We therefore interpret the DMR6 CTH as a dynamic contributing element rather than a fixed degron or direct contact site, whose recognition is enhanced by SA-induced conformational changes that modulate the overall surface topology for E3 engagement. To assess whether DAF1 contributes to the proteasomal degradation of DMR6 and DLO1, recombinant proteins were incubated with extracts from wild-type Col-0 or *daf1* T-DNA insertion mutant (SALK_072882; Supplementary Fig. 9b). Recombinant DMR6 and DLO1 both underwent rapid degradation in wild-type extracts, but degradation was delayed in *daf1* extracts (Fig. 4g, h), suggesting that DAF1 promotes but may not be the sole regulator of DMR6 and DLO1 proteins level. Consistently, in a ratiometric mScarlet-I-Venus degradation assay, co-expression of DAF1 led to decreased DMR6 fluorescence relative to control, indicating enhanced DMR6 degradation (Supplementary Fig. 9c). Endogenous DMR6 protein levels were faintly detectable in *daf1* and showed only a modest increase compared with Col-0 after MG132 treatment during SA exposure (Fig. 4i), indicating attenuated proteasomal degradation but overall low steady-state accumulation in the mutant background. In this assay, we imaged *daf1* only because endogenous DMR6 is low compared to Col-0, and co-exposing both genotypes on the same membrane bleaches the endogenous DMR6 signal relative to Col-0, making accurate measurement of protein levels difficult. The corresponding Col-0 comparison is shown in Supplementary Fig. 9d, where each genotype could be imaged under appropriate exposure conditions. These data reveal genotype-dependent differences in DMR6 abundance and are consistent with altered post-translational regulation in the absence of DAF1. Moreover, immunoprecipitation assays performed with the DMR6 antibody and Col-0 and *daf1* extracts coupled to Western blotting with a Ub antibody revealed diminished intensity of DMR6-Ub conjugates isolated from *daf1* extracts, further supporting aberrant DMR6 protein regulation relative to WT in the *daf1* background (Supplementary Fig. 9e). This result is consistent with published di-Gly proteomics datasets identifying DMR6/DLO1 as ubiquitinated and with our functional data showing DMR6 stabilization in *daf1*^11^. Overall, DMR6 ubiquitylation occurs at low stoichiometry and was detected at low intensity, consistent with transient ubiquitin adducts typical of proteasomal substrates. To examine whether disruption of SA perception affects DMR6 regulation, we analyzed DMR6 protein levels in the *npr1-1* mutant, which is defective in SA signaling. Immunoblotting revealed reduced DMR6 abundance in *npr1-1* compared to Col-0 (Supplementary Fig. 9f). Previous studies have shown that SA-induced DMR6 expression is attenuated in *npr1-1* and that TGA2, a transcription factor that interacts with NPR1, binds the DMR6 promoter to promote its transcription^37^. Consistent with these findings, reduced DMR6 protein levels in *npr1-1* indicate that SA-NPR1 signaling contributes to DMR6 induction.

Since DMR6 inactivates SA via hydroxylation, forming 2,5-DHBA, and decreased 2,5-DHBA was observed at multiple developmental stages in the *dmr6* mutant^6^, we quantified SA and 2,5-DHBA levels in Col-0 and *daf1* using LC–MS/MS across six biological replicates in four independent experiments (Supplementary Fig. 9g, h). While SA levels did not differ significantly between genotypes, 2,5-DHBA levels were lower in daf1 (*p* = 0.0036; Supplementary Fig. 9g, h), consistent with the reduced steady-state abundance of DMR6 protein observed in this background. A similar decrease in 2,5-DHBA was previously reported in *dmr6* mutants compared to *dmr6 dlo1* double mutants^6^, supporting the interpretation that altered DMR6 abundance may contribute to reduced 2,5-DHBA accumulation. Likewise, *dmr6* single mutants maintain near-wild-type SA levels across most developmental stages^6^, highlighting compensatory mechanisms that buffer SA homeostasis and mask changes in DMR6 expression or turnover. Although DAF1 acts as an F-box protein predicted to regulate DMR6 ubiquitination, the *daf1* mutant maintains low but detectable DMR6 protein levels. These patterns suggest a potential self-limiting feedback loop in which DMR6 abundance and stability are regulated by SA-mediated transcriptional induction and DAF1-mediated degradation that is enhanced by SA. Loss of DAF1 alters this balance and is associated with reduced 2,5-DHBA levels and altered DMR6 steady-state abundance.

Next, we examined whether the aberrant DMR6 regulation observed in *daf1* affects the growth of the bacterial pathogen *Pseudomonas syringae* pv. Tomato (*Pst*) DC3000^6^. In a syringe infiltration assay, the growth of *Pst* DC3000 after 1 days post-infiltration showed no significant differences comparing wild-type Col-0 to *daf1* mutants (6.04 log_10_ CFU cm^−2^ vs. 6.33 log_10_ CFU cm^−2^; *p* = 0.998) whereas at 3 days post-infiltration we observed increased susceptibility in *daf1* mutants (6.73 log_10_ CFU cm^−2^ vs. 7.84 log_10_ CFU cm^−2^; *p* < 0.0001) (Fig. 4j and Supplementary Fig. 10). Given that DMR6 acts as a negative regulator of immunity and DAF1 promotes DMR6 turnover, the immune phenotype of *daf1* depends on the net balance between transcriptional induction and post-translational control. This phenotype aligns with altered DMR6 regulation in the mutant background.

Having analyzed the molecular and immune-associated phenotypes of the *daf1* mutant, we next examined whether DAF1 overexpression elicits a biological response in planta. During optimization of DAF1 transient expression in *N. benthamiana* for co-IP assays, we consistently observed leaf necrosis exceeding the typical variation associated with *Agrobacterium*-mediated expression. We therefore systematically tested whether DAF1 overexpression triggers cell death. Remarkably, DAF1 overexpression alone induced visible necrosis beginning at ~2 dpi, whereas co-expression of DMR6 partially suppressed this phenotype (Fig. 4k and Supplementary Fig. 11a, b). Co-expression of DAF1 with the bacterial salicylate hydroxylase NahG^38^ similarly mitigated the necrosis (Supplementary Fig. 11c), suggesting that SA inactivation suppresses DAF1-induced cell death. DAF1 overexpression in *N. benthamiana* induced leaf necrosis, which was alleviated by co-expression of DMR6 or by SA depletion via NahG. Together with the enhanced *Pst* susceptibility observed in daf1 mutants, these findings indicate that perturbation of DAF1 activity influences immune outcomes. Collectively, the data are consistent with a coordinated regulatory relationship between DAF1 and DMR6 in modulating SA-associated immune responses.

### *Pst* DC3000 infection increases DMR6 protein levels and reshapes ASK1-associated SCF networks

**Fig. 5:**
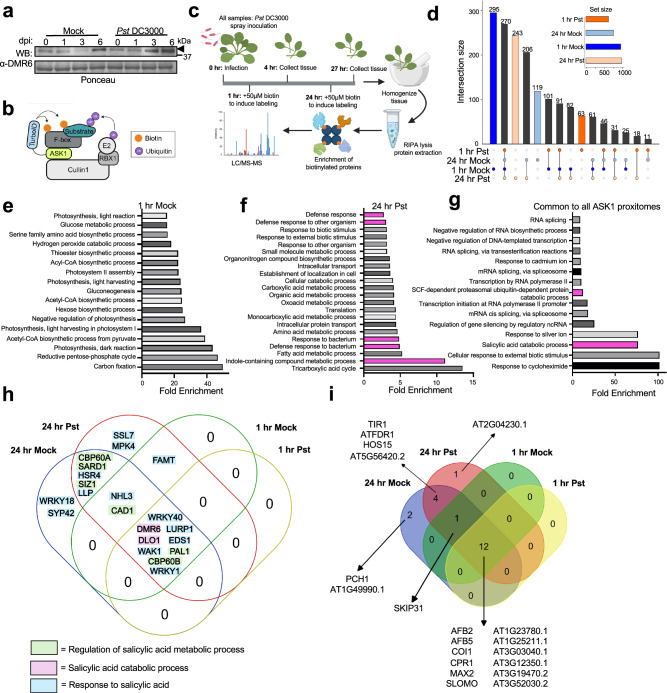
The SCF interactome during *Pst* DC3000 infection captures DMR6, DLO1, and various other putative substrates. **a** In vivo levels of DMR6 protein in Col-0 during *Pst* DC3000 infection. **b** Schematic representation of an SCF E3 and the TurboID fusion construct used. **c** Experimental workflow for *Pst* DC3000 infection, proximity labeling initiation, tissue collection, and generation of proteomic proxitome datasets. Created in BioRender. **d** Upset Plot depicting overlapping and distinct candidate ASK1 interactors identified across treatments and time points. Colored vertical histogram bars represent the number of unique ASK1 interactors for each treatment and time point. Selected GO biological processes terms enriched among ASK1 interactors of 1 h post-mock treatment (**e**), ASK1 interactors of 24 h post-infection (hpi) with *Pst* DC3000 (**f**), and ASK1 interactors found in all treatments and time points (**g**). Pink colored bars represent processes related either to immunity or the ubiquitin system. Venn diagrams depicting SA-related proteins enriched among ASK1 interactors (**h**) and F-box proteins enriched among ASK1 interactors (**i**). Elements in (**c**) were created in BioRender. Moe-Lange, J. (2026) BioRender.com/p3isv74.

DMR6 transcription is induced by a wide range of pathogens, including *Pst* DC3000 and oomycetes, in both Arabidopsis and crop species^39^. However, whether this transcriptional induction is reflected at the protein level remains unclear. Prior studies have shown that DMR6 transcripts accumulate from 1 to 6 days post-inoculation (dpi) with *Pst* DC3000^6^. Given that *Pst* DC3000 infection leads to a rapid and substantial increase in SA levels^40–42^, we hypothesized that DMR6 accumulation may help buffer this SA burst. Moreover, as *Pst* DC3000 suppresses SA-mediated immunity via coronatine, a jasmonate mimic, increased DMR6 levels may fine-tune SA levels during infection^43,44^. To test this, we infected Col-0 plants with *Pst* DC3000 and assessed DMR6 protein levels at 0, 1, 3, and 6 dpi using DMR6 antiserum. DMR6 levels increased steadily in infected tissue, peaking at 6 dpi (Fig. 5a). Although mock-treated plants showed a modest rise over time, DMR6 abundance was consistently higher in infected plants at all time points beyond 0 dpi, indicating that *Pst* DC3000 infection induces DMR6 accumulation at the protein level.

Given the *Pst* DC3000-induced accumulation of DMR6 protein and our previous identification of DMR6 and DLO1 as ASK1-associated proteins^10^, we reasoned whether *Pst* DC3000 infection is a factor that affects ASK1/SCF association with DMR6, DLO1, or other SA-related regulators. While DAF1 may contribute to the proteolysis of DMR6 and DLO1 under basal conditions, pathogen infection may further influence their protein abundance and regulatory interactions. In particular, *Pst* DC3000-induced SA accumulation and immune signaling could modulate SCF complex composition, altering target recruitment and degradation. These observations prompted us to examine how DMR6 protein levels and ASK1-associated networks are reshaped during bacterial infection, providing insight into context-dependent regulation of SA catabolic enzymes. More broadly, we asked whether infection triggers changes in SCF network composition that could influence substrate targeting. SCF E3 ligases are central regulators of hormone signaling and proteome dynamics, yet how their composition or substrate engagement shifts during pathogen infection remains largely unexplored. To capture dynamic SCF network interactions during infection, we performed TurboID-based proximity labeling of ASK1 in Arabidopsis during *Pst* DC3000 infection (Fig. 5b, c). Previous studies have shown that the ubiquitin system responds rapidly to bacterial pathogens, with significant ubiquitylome remodeling occurring within 30 min of pattern-triggered immunity (PTI) induction and broader proteomic shifts detected as early as 4 h post-infection^45,46^. Based on this temporal sensitivity, we performed proximity labeling 1 and 24 h post-infection (hpi) to capture early and sustained changes in ASK1-associated networks (Fig. 5c). To better simulate natural infection and avoid interference with the biotin infiltration that is performed to initiate labeling, we used foliar spray inoculation of a *Pst* DC3000 suspension supplemented with 0.02% Silwet L-77 on 5-week-old TurboID-ASK1 and citrine-TurboID Arabidopsis lines (Fig. 5c and Supplementary Fig. 12a). Bacterial recovery assays confirmed that all genotypes received an equivalent amount of inoculum and supported equal *Pst* DC3000 uptake and proliferation during the first 24 h of infection (Supplementary Fig. 11a). Following streptavidin pulldown, TMT labeling, and LC–MS/MS, we quantified between 1983 and 2420 proteins per sample across the four experimental conditions: 1 hpi mock, 1 hpi *Pst* DC3000, 24 hpi mock, and 24 hpi *Pst* DC3000 (Supplementary Fig. 12b–i). Based on a threshold of fold change ≥ 1.23 and *q* ≤ 0.1, 631–1002 proteins per dataset were designated as ASK1 interactors (Supplementary Data 2). Comparative analysis revealed dynamic shifts in ASK1-associated networks over time and across treatments (Fig. 5d and Supplementary Data 3). At 1 hpi, 295 proteins were unique to mock-treated plants and were enriched for GO biological process (BP) terms related to photosynthesis and carbon fixation (Fig. 5d, e, and Supplementary Data 4). In contrast, 1 hpi *Pst* DC3000-specific interactors showed no significant GO BP enrichment, but included several proteins previously linked to SA-mediated immunity (Supplementary Data 5). These included PAPP2C (AT1G22280.3), a phosphatase involved in SA-dependent basal defense^47^; PWR (AT3G52250.1), a chromatin regulator implicated in SAR activation^48^; and NUP82 (AT5G20200.1), a nuclear pore component associated with defense gene expression^49^. These findings are consistent with prior observations that photosynthetic activity is rapidly downregulated during *Pst* DC3000 infection and that flg22 treatment reduces the abundance of photosynthesis-related proteins^50,51^. At 24 hpi, a more distinct divergence emerged. Among the 243 proteins unique to 24 hpi *Pst* DC3000-treated samples, GO BP enrichment analysis revealed strong enrichment for defense-associated biological processes, including response to bacterium and indole-containing compound metabolism (Fig. 5f and Supplementary Data 4). By contrast, mock-specific interactors at 24 hpi were enriched only for general cellular organization and process terms (Supplementary Data 4). Several 24 hpi *Pst* DC3000-specific interactors, including MOS4, PEN3, and MPK4, have established roles in regulating immunity, further supporting infection-dependent remodeling of SCF-associated networks. Several ASK1 interactors identified during infection overlapped with previously reported ubiquitylated proteins^46^ (Supplementary Fig. 13 and Supplementary Data 5). Five were unique to 1 hpi of *Pst* DC3000, 43 were specific to 24 hpi of *Pst*, and 18 were found in all conditions, indicating broad integration of UPS signaling into immune-responsive SCF networks. Among the 270 proteins shared across all ASK1 proxitomes, GO BP enrichment analysis identified significant overrepresentation of SA catabolism, SCF-mediated ubiquitination, and proteasomal degradation (Fig. 4g). DMR6 and DLO1 were consistently enriched and annotated under SA catabolic process, indicating a stable association with ASK1 under both basal and infected states (Fig. 5h). Of the 21 SA-related proteins enriched across all datasets, 9 were common to all conditions, and four (WRKY18, MPK4, SYP42, and SSL7) were specific to *Pst* DC3000-infected samples, suggesting context-specific SCF-mediated regulation of SA signaling components during infection.

Twenty F-box proteins were identified across the datasets, including key hormone signaling adapters, such as TIR1, COI1, CPR1, and MAX2 (Fig. 5i and Supplementary Data 6). Twelve F-boxes were shared across all conditions, while others were specific to treatment or time points. For instance, AT2G04230 was unique to 24 hpi *Pst* DC3000 and has previously been linked to pathogen response. Conversely, PCH1 and AT1G49990 were exclusive to the 24 hpi mock. Several others, including HOS15 and ATFDR1, were found in both 24 hpi datasets and are implicated in hormone and immune signaling. Although HOS15 is known to mediate degradation of the SA receptor NPR1, its association with ASK1 appears to be infection-independent^52^. Together, these analyses demonstrate that, while ASK1 associates with DMR6, DLO1, and numerous F-box proteins constitutively, *Pst* DC3000 infection triggers a broader remodeling of the ASK1 interactome.

## Discussion

SA is a central immune phytohormone whose accumulation must be tightly controlled to balance defense and growth. Extensive work has established the biochemical roles of the SA hydroxylases DMR6 and DLO1 in modulating SA levels and catabolic products. Their transcription is rapidly induced by SA and pathogen infection, forming a negative feedback loop that limits prolonged immune activation^6,9^. Despite detailed knowledge of their enzymatic and transcriptional control, the post-translational mechanisms governing the stability of DMR6 and DLO1 have remained largely unexplored. Here, we provide evidence consistent with a proteasome-mediated regulatory circuit in which SA modulates the stability of its own catabolic enzymes through conformational regulation and E3 ubiquitin ligase-associated turnover. This dual layer of control contributes to dynamic SA homeostasis during immune activation and recovery. Endogenous DMR6 protein levels show modest yet consistent changes following SA treatment. Such subtle shifts are consistent with the biological role of DMR6 as a homeostatic SA-catabolic enzyme whose abundance reflects a balance between rapid transcriptional induction and proteasomal degradation. Seemingly low-amplitude yet biologically meaningful changes have been described for tightly regulated hormone components, including Aux/IAA proteins and EIN3, where proteostatic buffering yields limited steady-state variation while enabling substantial downstream signaling outputs^53^.

**Fig. 6:**
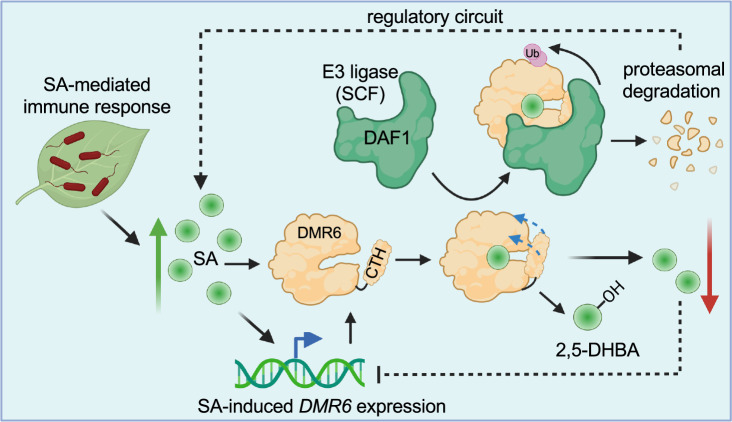
Proposed model for SA-mediated regulation of DMR6 stability and immune signaling via E3 SCF ligase complex. Salicylic acid (SA) accumulation during immune activation induces *DMR6* transcription, increasing DMR6 protein abundance. SA binding to DMR6 is associated with localized conformational remodeling of a conserved C-terminal helix (CTH). DMR6 interacts with the Kelch-type F-box protein DAF1, a component of an SCF-type E3 ubiquitin ligase complex, and undergoes ubiquitination and proteasomal degradation. Together, these findings support a regulatory circuit in which SA promotes both the expression and turnover of its catabolic enzyme. DMR6 hydroxylates SA to generate 2,5-dihydroxybenzoic acid (2,5-DHBA), contributing to attenuation of SA-mediated immune responses. The balance between transcriptional induction and proteasome-dependent turnover likely establishes a self-limiting feedback mechanism that fine-tunes SA homeostasis during plant immunity. Created in BioRender. Moe-Lange, J. (2026) BioRender.com/wn3tq5s.

Structural and biochemical analyses further define the determinants of DMR6 stability. DMR6 undergoes SA-accelerated proteasomal degradation, whereas DLO1 is stabilized by SA and destabilized when catalytically impaired. These contrasting responses align with prior models proposing that DMR6 modulates early immune responses, whereas DLO1 contributes later or in systemic tissues^6,9^. MD simulations show that SA binding induces closure of a conserved CTH in DMR6 but not in DLO1. In DMR6, this ligand-triggered conformational shift remodels surface topology in a manner consistent with increased accessibility to ubiquitin ligases (Fig. 6). Chimeric swaps further demonstrate that the DMR6 CTH enhances degradation when introduced into DLO1, although it does not independently confer SA-accelerated turnover. These findings indicate that the CTH functions as a degron-like module whose recognition can occur independently of SA-induced flexibility, while ligand-driven conformational dynamics enhance the efficiency of proteasomal targeting. A related ligand-dependent structural transition occurs in NPR1, where SA binding promotes conformational rearrangements required for transcriptional activation^54^. Together, these observations support the broader concept that SA can function as a structural switch that influences protein fate through allosteric control.

Catalytic state further modulates enzyme stability. The catalytically inactive DMR6 mutant exhibits increased stability, whereas the corresponding DLO1 mutant is destabilized relative to wild type. Although the transient expression system shows limited dynamic range, cell-free degradation assays and MG132-dependent stabilization provide stronger support for reduced turnover of catalytically inactive DMR6. Because the catalytic residue is buried within the active site and unlikely to directly contact an E3 interface, these stability differences most likely arise indirectly through activity-associated conformational dynamics. For DMR6, catalytic cycling may generate transient structural states that favor exposure of degradation determinants and enhance E3 engagement. In contrast, DLO1 activity appears to maintain structural integrity. Even in the absence of exogenous SA, DMR6 remains intrinsically unstable, likely reflecting sensitivity to basal endogenous SA levels detected in untreated plants. Together, these observations indicate that DMR6 degradation is influenced by catalytic activity, basal hormone levels, and structural plasticity acting in concert.

To identify the E3 ligase components contributing to this regulation, we performed TurboID-based proximity labeling and identified a previously uncharacterized Kelch-type F-box protein, which we term DMR6-associated F-box 1 (DAF1), as a candidate regulator of DMR6 and DLO1 stability. Functional analyses indicate that loss of *DAF1* slows degradation of both enzymes, reduces DMR6 ubiquitination, modestly alters SA and 2,5-dihydroxybenzoic acid profiles, and increases *Pst* DC3000 susceptibility. Ubiquitination and degradation are diminished but not abolished in the daf1 background, consistent with partial redundancy among plant E3 ligases. Conversely, DAF1 overexpression induces necrosis that is suppressed by DMR6 or NahG co-expression, indicating that DAF1 activity influences SA homeostasis in planta. Together, these findings are consistent with DAF1 functioning within an SCF-type E3 ligase complex that contributes to DMR6 turnover.

Our findings support a framework in which SA promotes DMR6 transcription while also facilitating its degradation through DAF1-associated proteasomal pathways, thereby constraining excessive hormone inactivation (Fig. 6). During pathogen infection, transcriptional induction and post-translational turnover operate within a broader immune network. Whereas exogenous SA provides an acute stimulus that favors proteasomal turnover, infection triggers coordinated SA biosynthesis, robust DMR6 transcription, and remodeling of SCF assemblies that modulate substrate engagement. Under these conditions, transcriptional upregulation can exceed degradation, resulting in net DMR6 accumulation, consistent with prior transcriptomic and metabolite data^6^. Although DMR6 and DAF1 function within the same regulatory module, they act at distinct levels. DMR6 directly attenuates SA signaling through hydroxylation, whereas DAF1 regulates DMR6 stability and thereby shapes SA homeostasis. The increased susceptibility of *daf1* indicates that disruption of this proteolytic layer perturbs immune balance rather than simply amplifying defense responses. Reduced DMR6 abundance in the *npr1-1* mutant further suggests that SA signaling through NPR1 contributes to DMR6 regulation, consistent with coordinated transcriptional and proteasomal inputs within the SA-DMR6-DAF1 module.

Large-scale ASK1-TurboID proximity labeling during *Pst* infection reveals extensive remodeling of SCF networks. Hundreds of proteins display dynamic association changes between early and late infection stages, including regulators of SA metabolism and defense signaling. DMR6 and DLO1 remain consistently associated with SCF assemblies, whereas infection promotes selective enrichment of additional F-box proteins and proteasome components. This dynamic reconfiguration provides a mechanistic basis for how *DMR6* can be transcriptionally induced yet only partially stabilized during infection and highlights that the UPS integrates metabolic and immune cues through flexible E3-substrate partnerships rather than fixed enzyme–ligase pairs.

The DMR6–DAF1 module exemplifies a broader class of self-limiting hormone feedback systems. Comparable architectures include the EIN3–EBF2 loop in ethylene signaling and the Aux/IAA–SCF^TIR1^ module in auxin signaling, in which transcriptional induction of an E3 adapter facilitates the degradation of its own activation^13,55–57^. Similarly, SA induces both DMR6 transcription and DAF1-mediated degradation, coupling synthesis and turnover to maintain homeostasis. At the molecular level, DMR6 mirrors other metabolite-sensitive E3 substrates. In strigolactone signaling, ligand-induced rearrangements of the receptor D14 enable recognition by the F-box protein MAX2^23,58^.

In contrast, in strigolactone catabolism, the carboxylesterases CXE15 and CXE20 undergo catalytic-state-dependent conformational shifts that modulate activity^29^. DMR6 appears to represent a parallel case, where SA binding remodels the conserved CTH to facilitate recruitment by the putative DAF1 E3 ligase. To corroborate the physical interaction with DAF1 and delineate the exact sequence of molecular events, future structural and biochemical studies will be essential to define how ligand-induced allosteric remodeling couples enzymatic activity to ubiquitin-mediated degradation. In addition, extending proximity-labeling approaches to the *daf1* background during pathogen challenge will help clarify how DAF1 shapes SCF-associated regulatory networks in vivo and whether infection alters its recruitment dynamics.

This dynamic reconfiguration suggests that SCF assemblies adjust substrate specificity to match immune needs, underscoring their central role in proteome plasticity. Our findings provide mechanistic evidence that SA hydroxylases are subject to post-translational regulation through the UPS. DMR6 thus joins a limited group of metabolite-responsive enzymes, including the jasmonate oxidase JOX2, whose stability is regulated through F-box-mediated degradation^12^. Together, these observations indicate that UPS sensitivity can be influenced by catalytic state and ligand-associated conformational changes, adding an additional layer to hormone feedback control.

Analogous regulatory strategies operate across biological kingdoms. In mammals, HMG-CoA reductase is degraded via sterol-sensitive degron exposure^59^, and prolyl hydroxylases regulate HIFα degradation in response to oxygen and metabolite availability^60^. In bacteria, SA-responsive transcription factors induce salicylate hydroxylases like *NahG* to modulate host-derived SA levels^61^. Although mechanistically distinct, these systems share a common principle: small molecules coordinate enzymatic activity with protein stability, thereby integrating metabolic regulation with proteostasis.

In summary, our results support a post-translational regulatory circuit where the SA hydroxylase DMR6 undergoes ligand-accelerated proteasomal degradation, in contrast to the stabilization observed for its paralog DLO1. We show in vitro and in silico that SA binding triggers a conformational closure of a conserved CTH in DMR6, which may contribute to remodeling surface topology to facilitate ubiquitination. Through proximity labeling, we identify DAF1 as a Kelch-type F-box protein that interacts with DMR6 and may promote its ubiquitination and proteasomal degradation. The observation that *daf1* mutants exhibit reduced DMR6 ubiquitination and altered 2,5-DHBA, combined with the finding that *npr1-1* further diminishes DMR6 abundance, supports a model in which transcriptional and proteasomal inputs are integrated within a SA-DMR6-DAF1 module. Finally, our large-scale proteomic analysis reveals that the SCF-associated network undergoes extensive remodeling during *Pst* DC3000 infection, suggesting that the dynamic regulation of these hydroxylases is part of a broader shift in the UPS during the plant immune response. Together, these observations suggest a multilayered feedback circuit in which SA functions both as an inducer and a destabilizer of its catabolic enzymes. This architecture may enable plants to fine-tune the balance between defense and growth and provides a framework for manipulating proteasome-dependent hormone homeostasis in crops. DMR6 orthologs are highly conserved and function as susceptibility factors across diverse plant lineages, suggesting that this proteolytic feedback mechanism may be broadly maintained. Loss of DMR6 orthologs in tomato, rice, grapevine, and barley confers enhanced resistance^20,39,62–64^, indicating that modulating DMR6 stability, rather than eliminating its expression, could offer a refined strategy for engineering durable immunity without growth penalties.

## Methods

### Plant material and growth conditions

Unless otherwise specified, *Arabidopsis thaliana* ecotype Columbia-0 (Col-0) plants and *Nicotiana benthamiana* plants were grown at 22 °C under 24 h light and 16 h:8 h light:dark photoperiod, respectively. To generate stable transgenic pUBQ::DMR6-3xHA lines, wild-type Col-0 were transformed with *Agrobacterium* harboring the pUBQ::DMR6-3xHA vectors via the floral dip method. Transformants were identified via glufosinate ammonium (Basta) selection on 0.5 Murashige & Skoog (MS) + 0.8% agar plates. Expression of DMR6-3xHA was confirmed via Western blotting (Supplementary Fig. 1a). T2 individuals were used in subsequent experiments following the Basta selection.

T-DNA insertion mutant (SALK_072882) for Kelch F-box AT4G00893 was obtained from the Arabidopsis Biological Resource Center in Columbus, Ohio (https://abrc.osu.edu/). Presence of the T-DNA insertion and absence of the wild-type locus-specific product was confirmed via PCR using the primers listed in Supplementary Data 7.

### DMR6 antiserum generation

A custom antiserum against DMR6 using a synthetic peptide corresponding to residues 298–312 (KPLWEAEDDETKPVY) was generated through the PhytoAb antibody production service. Antiserum was validated by blotting against recombinant DMR6 and DLO1 as well as native extracts from Col-0 and pUBQ::DMR6-3xHA (Supplementary Fig. 1b, c).

### Plasmid construction

All primers used can be found in Supplementary Data 7. For recombinant protein expression constructs, DMR6 and DLO1 were amplified with the appropriate adapter sequences via PCR from the p35S:DMR6/DLO1-^YN^3xMyc vectors used in Sun et al.^10^. The respective fragments were recombined into pAL backbone vectors with either a 6xHis-mysB or 6xHis-SUMO fusion tag. DMR6^D214A^ and DLO1^D223A^ point mutants were created via site-directed mutagenesis with the pAL-6xHis-mysB constructs. To make HA-tagged expression constructs and TurboID fusion constructs, genes were amplified from plasmid DNA via PCR and combined into the pUBQ::MCS-3xHA or pUBQ::TurboID-3xmyc-MCS backbone via the Takara In-Fusion Snap Assembly cloning kit.

### Recombinant protein preparation and purification

A 120 mL flask of BL21 *Escherichia coli* harboring the desired plasmid was grown in Luria-Bertani (LB) broth + 100 mg/mL chloramphenicol at 37 °C overnight. Two to six liters of LB with 100 mg/mL chloramphenicol were subsequently seeded with 13 mL each of start culture, grown at 37 °C to an OD_600_ of ~0.6, and induced with 25 mM IPTG (isopropyl ß-D-1-thiogalactopyranoside) at 16 °C for 16 h. Cells were harvested via centrifugation and resuspended in lysis buffer (50 mM Tris, 8, 300 mM NaCl, 1 mM β-mercaptoethanol, 5 mM imidazole, protease inhibitor cocktail). Resuspended cells were lysed using an Avestin Emulsiflex C3 high-pressure homogenizer. The resulting lysate was clarified via centrifugation, and the 6xHis-SUMO or mysB fusion proteins were isolated from the soluble fraction with Ni-NTA resin. For brevity, the 6xHisSUMO-DMR6 and 6xHis-mysB-DMR6 recombinant proteins are annotated as His-DMR6 in figures. The resin was washed with buffer containing 50 mM Tris-HCl, pH 8, 500 mM NaCl, 10 mM imidazole, eluted with 250 mM imidazole in 50 mM Tris-HCl, pH 8, and subjected to anion exchange chromatography (IEX). To generate tag-less protein, selected eluted fractions from IEX were combined and cleaved overnight at 4 °C with tobacco etch virus protease. Cleaved tag was removed by passing over Ni-NTA resin and further purified via size exclusion chromatography over a Superdex 75 increase 10/300 column in 20 mM Tris-HCl, pH 8, 300 mM NaCl, 8% glycerol, and 5 mM DTT. Proteins were concentrated to 2–7 mg/mL using 30 K MWCO Amicon^®^ Ultra Centrifugal Filter and stored at −80 °C.

### *Agrobacterium*-mediated transient expression

*Agrobacterium tumefaciens* strain GV3101 was transformed with expression constructs for the proteins of interest. Overnight cultures were grown at 28 °C and adjusted to an OD_600_ = 0.5 (final concentration when mixed 1:1) for co-immunoprecipitation assays and OD_600_ = 1.0 for other experiments and infiltrated into 5-week-old wild-type N. benthamiana leaves with a needless syringe.

### Co-immunoprecipitation assay

Co-immunoprecipitation (Co-IP) experiments involving DMR6 and DAF1/mCitrine were performed following established protocols^10^ (Sun et al.). Briefly, *N. benthamiana* leaves co-expressing the indicated constructs (pUBQ::DMR6-3xHA, pUBQ::citrine-3xMyc, or pUBQ::DAF1-3xMyc) were harvested and flash-frozen in liquid nitrogen. The frozen tissue was ground to a fine powder and homogenized in 2.5 volumes (w/v) of Co-IP extraction buffer (25 mM Tris-HCl, pH 7.5, 1 mM EDTA, 150 mM NaCl, 10% glycerol, 0.15% NP-40, 10 mM DTT, 1 mM PMSF, 2% PVPP, and a protease inhibitor cocktail from Roche). The resulting lysate was mixed by vortexing and inverting for 30 min at 4 °C and then clarified by two consecutive high-speed centrifugations for 10 min each. The cleared supernatant was incubated for 3 h at 4 °C with antibody-conjugated beads that had been pre-equilibrated in wash buffer (extraction buffer without PVPP). Following incubation, the beads were collected using a magnetic rack and washed three times with the wash buffer. Finally, immunoprecipitated proteins were eluted by resuspending the beads in 2× sample buffer and boiling for 5 min.

### SDS-PAGE and Western blotting

Unless otherwise indicated, proteins were separated using 13% SDS-PAGE gels and transferred to nitrocellulose membranes. Prior to blocking, total protein was visualized via Ponceau S stain. Following blocking in 5% milk in 0.01% TBST, proteins were detected via their respective antibodies: Ubiquitin with ubiquitin monoclonal antibody at a 1:1000 dilution (Enzo BML-PW0930), His-tagged proteins with monoclonal 6x-His (Invitrogen MA1-21315) at a 1:2000 dilution, HA-tagged proteins with monoclonal HA-HRP at 1:2000 (Invitrogen #26183-HRP), and myc-tagged proteins with monoclonal myc-HRP at 1:2000 (Invitrogen #R951-25). For non-HRP-conjugated monoclonal antibodies, secondary antibody goat anti-mouse-HRP was used at a 1:2000 dilution (Invitrogen #32430). For imaging, SuperSignal™ West Femto Maximum Sensitivity Substrate was used as the chemiluminescent substrate, and a ChemiDoc™ Touch Imaging System (Bio-Rad) was used for western visualization. Relative band intensity was quantified using Bio-Rad Image Lab. All uncropped gel images are found in Supplementary Figs. 15 and 16.

### Cell-free degradation assays

Native protein extracts were obtained from wild-type Col-0 flower buds with the Minute™ Total Protein Extraction Kit for plant tissues as previously described^65^. For a 12.5 μL reaction, 20 μg extract was combined with 40 mM Tris-HCl, pH 8, 5 mM MgCl_2_, 2 mM DTT, 0.8 mg/mL ubiquitin, 4 mM ATP, 2 μg recombinant protein, and 100 μM SA or the equivalent amount of ethanol. In all experiments, SA was dissolved in ethanol as previously described; parallel assays using sodium salicylate in water yielded indistinguishable results (Supplementary Fig. 14). Reactions containing MG132 were spiked with 1 μL of 1 mM MG132. Reactions were incubated at 28 °C and terminated at the indicated time points by adding 4.5 μL 4X Laemmli sample buffer and boiling at 95 °C for 3 min. Samples were visualized via SDS-PAGE and Western blotting.

### Cycloheximide chase and proteasomal inhibition assays

To assess levels of transiently overexpressed DMR6 and DLO1 protein, leaves from 5-week-old *N. benthamiana* plants were infiltrated as described above. For cycloheximide (CHX) chase assays, 30 h later, the leaves were re-infiltrated with 1 mM SA (or the equivalent amount ethanol) and 5 μg/μL CHX. For proteasomal inhibition assays, leaves were infiltrated with 25 μM MG132 or the equivalent amount DMSO 30 h post-infiltration, and tissue was collected 6 h later. For both assays, at least 6 leaves from three *N. benthamiana* plants were infiltrated for each condition, and an equivalent amount of tissue was collected from each leaf, pooled, and flash frozen for each time point. To assess DMR6 protein levels both endogenously and in a stable overexpression context, wild-type Col-0, stable transgenic pUBQ::DMR6-3xHA, or SALK_072882 T-DNA mutant seeds were surface-sterilized with 70% ethanol and 0.1% Triton X-100 and plated on 0.5-strength MS media with 8% agar. Seeds were vernalized at 4 °C for 2 days and then moved to 24-h light for 2 weeks. Seedlings were subsequently transferred to 15 mL sterile liquid 0.5 MS media in Petri dishes at 6:00 PM and acclimated overnight. At approximately 9:00AM, an additional 15 mL 0.5 MS media was added for final concentration of 500 μM SA and 25 μM MG132, or the equivalent amount of ethanol (SA solvent) or DMSO (MG132 solvent). Following the *t* = 0 time point, the seedlings were incubated with the respective mixtures of SA with MG132 or controls, and at the indicated time points, seedlings from each group were blotted dry and immediately flash-frozen in liquid nitrogen. Tissue was homogenized and subjected to native protein extraction for subsequent Western blotting analysis. All *in planta* degradation assays were repeated three to five times.

### Ratiometric degradation assays

Three-week-old *N. benthamiana* plants were used to express various construct combinations by *Agrobacterium* (GV3101 pMP90)-mediated transient transformation of lower epidermal leaf cells as previously described by Khosla et al. with slight modifications^66^. To generate ratiometric constructs for degradation assays in *N. benthamiana*, DMR6 and DLO1 entry clones were transferred into pRATIO4212 destination vectors by gateway LR reaction. *A. tumefaciens* cultures were resuspended in infiltration buffer (10 mM MgCl₂, 10 mM MES pH 5.8, 150 µM acetosyringone) prior to injection into *N. benthamiana* leaves. *A. tumefaciens* containing ratiometric reporter vectors and a plasmid expressing p19 (suppressor of gene silencing) were co-injected at an optical density at 600 nm (OD_600_) of 0.24 and 0.16, respectively. For the DMR6 degradation assay with the presence of DAF1, A. *tumefaciens* containing ratiometric reporter vectors, p19, and pUBQ10::DAF1-3xmyc or its control pUBQ10::GUS-3xmyc were co-injected at OD_600_ of 0.3, 0.15, and 0.001, respectively. For degradation assays, 96-well black polystyrene plates (Corning Costar) were filled with 200 µl of chemical treatments (10 µM or 1 mM SA, or 0.01% (v/v) ethanol). Leaf discs were excised 2 days post-infiltration and transferred to the plate (one leaf disc per well) with the abaxial side facing up. The relative abundance of reporter proteins was measured using a SYNERGY H1 microplate reader (BioTeK) after 6 h of chemical treatment. mScarlet-I (reporter) was monitored using excitation at 560 nm and emission at 595 nm; Venus (reference) was measured using excitation at 497 nm and emission at 540 nm. Protein abundance was quantified as the mScarlet-I:Venus ratio after background subtraction using leaf discs transformed with equivalent concentrations of *Agrobacterium*, where pUBQ10::GUS-3xmyc was substituted for the reporter construct while maintaining identical concentrations of all other constructs, including pUBQ10::DAF1-3xmyc.Each data point represents the average ratio of mScarlet-I to Venus fluorescence of 3–6 leaf discs from a single transformed leaf (*n* = 4–6 leaves).

### Differential scanning fluorimetry

Reactions were prepared in triplicate with 40 μM recombinant protein incubated with 100 μM SA or the equivalent amount of ethanol in a buffer containing 20 mM Tris-HCl, pH 8, 300 mM NaCl, 8% glycerol, 0.4 mM FeSO_4_, and 5 mM DTT. Following 15 min of incubation with SA, 0.5 mM Sypro Orange was added and used as the reporter dye. Samples were subsequently heat denatured using a linear 4–95 °C gradient at a rate of 1 °C per min. Protein unfolding was monitored by detecting changes in Sypro Orange fluorescence using a Bio-Rad CFX96 real-time system with HEX emission (533 nm) and excitation (559 nm) fluorophore settings.

### AlphaFold structure prediction and molecular docking of salicylic acid

The three-dimensional structures of DMR6 (UniProt Q9FLV0) and DLO1 (UniProt Q9ZSA8) were obtained from the AlphaFold Protein Structure Database (AF-DB)^67^. The top-ranked models were selected based on model confidence scores (pLDDT) and inspection of the predicted aligned error maps. Both models displayed high confidence in their overall structure and particularly in within the catalytic core region. To accurately position the catalytic Fe³⁺ ion, each AF-DB model was structurally aligned with the experimentally solved 2-OG-dependent oxygenase from *Arabidopsis thaliana* (PDB ID: 6LSV), which shares greater than 40 % sequence and structural identity with DMR6 and DLO1. The Fe³⁺ ion from 6LSV was transferred into the aligned active sites, preserving the canonical H–D–H metal-binding geometry. The Fe³⁺-fitted models were then refined using the protein preparation Wizard in Schrödinger’s Maestro, optimizing hydrogen bonding, protonation states, and side-chain orientations, followed by restrained energy minimization to relieve local steric clashes and ensure correct coordination geometry. Molecular docking of SA into the Fe³⁺-bound DMR6 and DLO1 models was performed using Maestro Schrödinger Glide (release 2025-2)^68,69^. Proteins were preprocessed with the Protein Preparation Wizard^70^ at neutral pH 7.0, while the ligand was prepared using LigPrep, generating low-energy conformers at pH 7.0 ± 0.5. Docking was carried out using induced-fit docking (IFD)^71^ in standard precision (SP) mode, with the docking grid centered on the Fe³⁺ coordination site defined by the H–D–H catalytic triad. Metal-binding constraints were applied to maintain correct Fe³⁺ coordination during docking. Docked poses were evaluated based on GlideScore, Glide E-model, and total binding energy. The lowest-energy pose from each complex was selected for subsequent MD simulations, ensuring preservation of the Fe³⁺ coordination geometry observed in the homologous template. These refined protein–ligand complexes served as the starting structures for the MD simulations described below.

### Molecular dynamics simulations

All-atom MD simulations were performed using GROMACS (version 2023.3)^72^ with the CHARMM36m force field and TIP3P water model. The initial coordinates were prepared using the CHARMM-GUI interface^73,74^, where each protein–ligand complex was solvated in a rectangular periodic box (110 × 110 × 110 Å) ensuring a minimum distance of 12 Å between any solute atom and the box edge. The system was neutralized and adjusted to a physiological salt concentration of 150 mM NaCl by Monte Carlo placement of Na⁺ and Cl^−^ ions.

Electrostatic interactions were calculated using the particle Mesh Ewald method^75^ with a Fourier grid spacing of 0.115 nm and a real-space cutoff of 1.2 nm. Van der Waals interactions were treated with a cutoff of 1.2 nm using a force-switching function between 1.0 and 1.2 nm. All covalent bonds involving hydrogen atoms were constrained using the LINCS algorithm, allowing a 2 fs integration timestep. After steepest-descent energy minimization, equilibration was performed in two stages. The first stage (NVT) consisted of a 1 ns simulation at 300 K using the V-rescale thermostat (coupling constant τ_t_ = 1 ps) with position restraints on heavy atoms of the protein and ligand to relax solvent and ions. The second stage (NPT) consisted of a 1 ns simulation at 1 bar using the Parrinello–Rahman barostat (isotropic coupling, τ_p_ = 5 ps, compressibility 4.5 × 10^−^^5^ bar⁻^1^), maintaining the same thermostat settings as in NVT. Position restraints were retained during NPT to prevent large structural deviations. Production simulations were then performed in the NPT ensemble for 100 ns at 300 K and 1 bar with no restraints. Each system was simulated in triplicate using different random initial velocities to ensure reproducibility. Periodic boundary conditions were applied in all directions, and center-of-mass motion was removed every 100 steps. Trajectories were saved every 10 ps for analysis. Trajectory analyses included calculation of RMSD, root-mean-square fluctuation (RMSF), ligand RMSD, and hydrogen-bond and Fe³⁺-coordination distances between the ligand and active-site residues. In addition, the distance between the CTH and the core domain was computed to quantify domain opening and closure. Representative conformations were extracted based on the 10th and 90th percentile values of the CTH-core distance distributions, corresponding to closed and open states, respectively (summary of parameters in Supplementary Table 1). Pocket volume and surface-area analyses were performed on these representative structures using KVFinder^76^.

All graphs were plotted using Xmgrace, [Turner P (2005) XMGRACE, version 5.1. 19. Center for Coastal and Land-Margin Research, Oregon Graduate Institute of Science and Technology, Beaverton, OR] and the plots with mean ± SD were generated in GraphPad Prism (version 10.0). Molecular visualizations were produced using PyMOL version 3.0. (http://www.pymol.org/).

### Trypsin-limited proteolysis

In a buffer containing 20 mM Tris-HCl, pH 8, 300 mM NaCl, 8% glycerol, 0.4 mM FeSO4, and 5 mM DTT, 5 μg recombinant protein (DMR6, DLO1, DMR6^DLO1-CTH^, or DLO1^DMR6-CTH^) were incubated on ice for 15 min in the presence of 100 μM SA or an equivalent amount EtOH. At time (*t*) = 0, 1 μg/mL trypsin diluted in the same buffer was added to the protein-SA/EtOH mixtures. At each time point, proteolysis was terminated by adding 4X Laemmli sample buffer and immediately boiling at 95 °C. Samples were visualized by SDS-PAGE followed by Coomassie blue staining. All uncropped images are found in Supplementary Figs. 15 and 16.

### Protein sequence conservation analysis

DMR6, DLO1, or AT4G00893 homologs were identified using NCBI BLASTp with Arabidopsis DMR6, DLO1, or AT4G00893 as the respective query sequences. Homolog sequence alignment and conservation analysis were performed in CLC Genomics Workbench v22.

### *Pst* DC3000 infection and bacterial recovery assays

For the *Pst* resistance phenotyping of Col-0 vs *daf1* genotypes, syringe infiltration was performed. *Arabidopsis thaliana* (4–5 weeks old) were leaf-infiltrated using a needleless syringe with *Pseudomonas syringae* pv. Tomato DC3000 (~10^4^ CFU/mL in 10 mM MgCl_2_). To quantify in planta bacterial growth, six leaf discs (0.384 cm^2^ each) were collected per biological replicate at 1 and 3 days post-inoculation (dpi). Samples were homogenized in 1 mL of 10 mM MgCl_2_ using a TissueLyser, and the resulting homogenate was serially diluted. Bacterial titers were determined by plating 3 μL droplets of each dilution onto King’s B medium supplemented with 100 μM Rifampicin. Droplets were plated in duplicate on two independent plates. Plates were incubated at 28 °C for 48 h, and CFU were quantified and expressed as CFU/cm^2^.

In the TurboID proximity labeling of ASK1 during *Pseudomonas syringae* pv. Tomato DC3000 (*Pst* DC3000) infection, spray inoculation was performed following a protocol adapted from Yao et al.^77^. Arabidopsis plants were grown in soil at 20 °C and 70–80% relative humidity with a 12 h:12 h light (100–150 μE/m^2^/s) /dark photoperiod. Five-week-old Arabidopsis plants were spray inoculated with a *Pst* bacterial suspension diluted to OD_600_ = 0.25 in sterile water with 0.02% Silwet L-77, corresponding to 1.25 × 10^8^ CFU/mL. The mock infection consisted of sterile water with 0.02% Silwet L-77 under the same growth conditions. After spraying, plants were covered with a plastic lid to maintain humidity and returned to the growth chamber. Bacterial recovery assays were also performed as previously described^77^. In brief, three leaves for each genotype and condition were harvested at the indicated time points. Leaves were surface sterilized by gently shaking in 70% EtOH for 10–15 s, rinsed in sterile water, and blotted dry. One 0.5 cm^2^ disc was punched from each leaf and placed in a 1.5 mL tube with 100 μL sterile water. Leaf tissue was homogenized with a plastic micropestle. An additional 900 μL sterile water was added to each tube, and serial dilutions of this mixture were performed to 10^−3^ by mixing 100 μL suspension with 900 μL sterile water. 10 μL from each sample were pipetted onto NYGA with Kanamycin_25_ Rifampicin_100_ agar plates in triplicate. The plates were subsequently incubated at 28 °C for 30 h. Colonies were counted and used to calculate bacterial CFU/cm^2^. Two-way ANOVA analysis was performed using GraphPad Prism to compare mean bacterial CFU/cm^2^ between genotypes and timepoints.

### Leaf imaging and analysis

Images of detached leaves were captured at 600 DPI using a flatbed scanner (Epson Perfection V600). The resulting RGB images were analyzed using Fiji/ImageJ (Version 1.53, NIH)^78^. To specifically quantify the area of trypan blue staining, images were converted to 8-bit grayscale, and a consistent manual threshold was applied across all images to create a binary mask of the stained areas. The total leaf area was determined by creating a separate binary mask of the entire leaf from the original image. Using the analyze particles function, the area of trypan blue staining and the total leaf area were measured from their respective masks. Cell death was expressed as the percentage of the stained area relative to the total leaf area. The analysis was performed on at least three biological replicates for each condition.

### Proximity labeling and isolation of biotinylated proteins

For Arabidopsis proximity labeling experiments, TurboID-ASK1 and citrine-TurboID stable transgenic lines were syringe-infiltrated with 50 μM biotin 1 and 24 h post *Pst* infection.

For *N. benthamiana* proximity labeling experiments, leaves of 5-week-old *N. benthamiana* plants were infiltrated with Agrobacterium harboring TurboID-DMR6, TurboID-DLO1, and citrine-TurboID. 48 h later, 50 μM biotin was applied to the previously infiltrated leaves.

For both Arabidopsis and *N. benthamiana* experiments, leaf tissues were collected by genotype and treatment, pooled, and flash-frozen after 3 h biotin incubation. To isolate biotinylated proteins, native protein extraction and affinity purification were carried out based as previously described^79^. In brief, the frozen tissues were homogenized using a mortar and pestle. One gram of tissue was used per replicate and incubated at 4 °C for 30 min with RIPA lysis buffer (50 mM Tris-HCl, pH 7.5; 500 mM NaCl; 1 mM EDTA; 1% NP-40 (v/v); 0.1% SDS (w/v); 0.5% deoxycholate (w/v); 1 mM DTT; and Roche protease inhibitor cocktail). The soluble fraction was collected by centrifugation at 16,500 × *g* for 10 min and then passed through a ZebaTM Spin Desalting Column (Thermo Fisher Scientific, Waltham, MA, USA) with a 7 K MWCO to remove free biotin not bound to proteins. Protein concentrations of the desalted samples were determined using the Bradford assay. To enrich the biotinylated proteins, 200 µL of DynabeadsTM MyOneTM Streptavidin C1 (Thermo Fisher Scientific) were pre-washed with RIPA buffer and then incubated with 6 mg of the desalted protein extract. The mixture was rotated at 4 °C for 16 h, followed by washes at room temperature with 1.7 mL of buffer I (2% SDS), 1.7 mL of buffer II (50 mM HEPES, pH 7.5; 500 mM NaCl; 1 mM EDTA; 0.1% deoxycholic acid; 1% Triton X-100), and 1.7 mL of buffer III (10 mM Tris, pH 7.4; 250 mM LiCl; 1 mM EDTA; 0.1% deoxycholic acid; 1% NP-40). The beads were then transferred to 4 °C, moved to a new tube, and washed with 1.7 mL of 50 mM Tris, pH 7.5, followed by 6× 1 mL washes with 50 mM ammonium bicarbonate. After the final wash, 30 µL of bead resuspension from each replicate was collected for immunoblot analysis. The supernatant containing ammonium bicarbonate was subsequently discarded, and the beads were flash-frozen in liquid nitrogen and stored at −80 °C for subsequent LC–MS/MS analysis.

### LC–MS/MS detection of biotinylated proteins for proximity labeling

Following isolation of biotinylated proteins from total extracts from four biological replicates via streptavidin bead affinity purification, enriched samples were eluted from beads by incubation at 95 °C for 10 min in 1x S-Trap lysis buffer (5% SDS, 50 mM TEAB, pH 8.5) supplemented with 12.5 mM biotin. Eluted samples were subjected to S-Trap sample processing technology (Catalog number C02-micro-80, ProtiFi, USA), following the manufacturer protocol. Samples were reduced in 2 mM TCEP, alkylated in 50 mM iodoacetamide (IAM), and digested into peptides at 37 °C in one round of overnight incubation with 1 µg of trypsin and a second incubation of 4 h with 0.1 µg trypsin plus 0.1 µg Lys-C. Peptides were further desalted using SepPack C18 columns (Waters). Tandem Mass Tag (TMT, Thermo Scientific) labeling was performed on purified peptides from each sample as previously reported^80^. TMT labeling reaction was stopped using 5% hydroxylamine, and the quenched samples were then pooled into three multiplexes (one multiple for the DMR6-DLO1 experiment and one multiplex for each time point of the ASK1 *Pst* experiment). Each ASK1-*Pst* multiplex included two pooled references to account for between-plex technical differences^81^.

All three multiplexes were subjected to high pH fractionation using Pierce High pH Reversed-Phase Peptide Fractionation Kit (Thermo Scientific) following the manufacturer's instructions. The obtained 8 fractions were further concatenated (pooled) as follows: fraction 1 with fraction 5, fraction 2 with fraction 6, fraction 3 with fraction 7, and fraction 4 with 8. Samples were finally dried on a SpeedVac and resuspended in 0.1% Optima™ grade formic acid (Fisher, Cat. no. A117-50) in Optima™ grade H_2_O (Fisher, Cat. no. W64). An injection volume of 20 µL, containing 1.2 µg from each concatenated fraction, was used for LC–MS/MS analysis.

For Arabidopsis proximity labeling experiments, chromatography was performed on a Thermo UltiMate 3000 UHPLC RSLCnano. Peptides were desalted and concentrated on a PepMap100 trap column (300 µM i.d. × 5 mm, 5 µm C18, 100 Å µ-Precolumn, Thermo Scientific) at a flow rate of 10 µL min^−1^. Sample separation was performed on a 200 cm Micro-Pillar Array Column (µ-PAC, Pharmafluidics) with a flow rate of ~300 nL min^−1^ over a 150 min reverse phase gradient (80% ACN in 0.1% FA from 1% to 15% over 5 min, 15% to 20.8% over 20 min, from 20.8% to 43.8% over 80 min, from 43.8% to 99.0% in 11 min and kept at 99.0% for 5 min). Eluted peptides were analyzed using a Thermo Scientific Q-Exactive Plus high-resolution quadrupole Orbitrap mass spectrometer, which was directly coupled to the UHPLC. Data-dependent acquisition was obtained using Xcalibur 4.0 software in positive ion mode with a spray voltage of 2.3 kV and a capillary temperature of 275 °C and an RF of 60. MS1 spectra were measured at a resolution of 70,000, an automatic gain control (AGC) of 3e6 with a maximum ion time of 100 ms and a mass range of 400–2000 m/z. Up to 15 MS2 were triggered at a resolution of 35,000. A fixed first mass of 115 m/z. An AGC of 1e5 with a maximum ion time of 50 ms, a normalized collision energy (NCE) of 33, and an isolation window of 1.3 m/z were used. Charge exclusion was set to unassigned, 1, 5–8, and >8. MS1 that triggered MS2 scans were dynamically excluded for 25 s.

For *N. benthamiana* proximity labeling experiments, chromatography was performed on a Thermo Vanquish Neo UHPLC in “heated trap-and-elute, backward flush” mode. Peptides were desalted and concentrated on a PepMap Neo trap column (300 µM i.d. × 5 mm, 5 µm C18, 100 Å µ-Precolumn, Thermo Scientific) at a flow rate of 10 µL min^−1^. Sample separation was performed on a 110 cm Micro-Pillar Array Column (µ-PAC Neo, Thermo Scientific) with a flow rate of ~300 nL  min^−1^ over a 120 min reverse phase active gradient (80% ACN in 0.1% FA from 15 to 43.8% over 109 min, from 43.8% to 62.5% for 11 min). Followed by a column/trap wash at 80% ACN for 10 min. Eluted peptides were analyzed using a Thermo Scientific Orbitrap Exploris 480 mass spectrometer with a FAIMS Pro Duo interface installed, which was directly coupled to the UHPLC through an Easy Spray Ion source (Thermo Scientific). Data-dependent acquisition was obtained using Xcalibur 4.0 software in positive ion mode with a spray voltage of 2.1 kV, a capillary temperature of 280 °C, an RF of 45, FAIMS compensation voltages of −45 and −60, and a total carrier gas flow of 4.6 l/min. MS1 spectra were measured at a resolution of 120,000, an AGC of 3e6 with auto maximum ion time, and a mass range of 400–1400 m/z. A cycle time of 0.8 s was used to capture triggered MS2 at a resolution of 15,000 with the “Turbo TMTpro” setting on. A fixed first mass of 110 m/z. An AGC of 1e5 with a maximum ion time of 22 ms, a NCE of 33, and an isolation window of 0.7 m/z were used. Charge inclusion was set to 2–6. MS1 that triggered MS2 scans were dynamically excluded for 30 s.

### Proteomics data and gene ontology (GO) analyses

For Arabidopsis proximity labeling experiments, raw data were analyzed using MaxQuant version 2.4.2.0^82^, spectra were searched, using the Andromeda search engine^83^, against the Arabidopsis TAIR10 annotation (www.arabidopsis.org). The proteome files were complemented with reverse decoy sequences and common contaminants by MaxQuant. Carbamidomethyl cysteine was set as a fixed modification, while methionine oxidation and protein N-terminal acetylation were set as variable modifications. The sample type was set to “Reporter Ion MS2” with “TMT18plex” selected for both lysine and N-termini. Digestion parameters were set to “specific” and “Trypsin/P;LysC”. Up to two missed cleavages were allowed. A false discovery rate, calculated in MaxQuant using a target-decoy strategy^84^, less than 0.01 at both the peptide spectral match and protein identification level was required. Significant interactors were assessed using the TMT-NEAT R pipeline^85^ with the PoissonSeq R package^86^.

For *N. benthamiana* proximity labeling experiments, MaxQuant version 2.5.2.0 was used. Spectra were searched using the Andromeda search engine^83^, against a concatenated FASTA file containing the following databases: NbDE, Sol Genomics v2.6.1, NbeHZ1 v1.0, Lab360v103, and Qld183v103^87–90^. The proteome files were complemented with reverse decoy sequences and common contaminants by MaxQuant. Carbamidomethyl cysteine was set as a fixed modification, while methionine oxidation and protein N-terminal acetylation were set as variable modifications. The sample type was set to “FAIMS-reporter Ion MS2” with “TMT18plex” selected for both lysine and N-termini. Digestion parameters were set to “specific” and “Trypsin/P;LysC”. Up to two missed cleavages were allowed. A false discovery rate, calculated in MaxQuant using a target-decoy strategy^84^, less than 0.01 at both the peptide spectral match and protein identification level was required. Significant interactors were assessed using the TMT-NEAT R pipeline^85^ with the PoissonSeq R package^86^.

For ASK1 data sets, ASK1 significant interactors were designated as candidates with a *q* ≤ 0.1 and linear fold change ≥ 1.23 (ASK1:citrine) as previously described^10^. Overlapping and distinct ASK1 interactors between time points and treatments were determined and visualized using the R packages UpsetR and ggplot2. Significant enrichment of GO::biological processes (BPs) among ASK1 interactors was performed using the PANTHER-powered GO statistical overrepresentation tool from The Arabidopsis Information Resource (TAIR). ASK1 interactors that were previously connected to immunity or the UPS were identified through a combination of manual curation through literature searches and comparison with the previously described ubiquitylome data set^46^. SA-related proteins were identified as proteins part of the following BPs: (GO:0010337) regulation of SA metabolic process, (GO:0046244) SA catabolic process, (GO:0009751) response to SA.

For DMR6 and DLO1 data sets generated from *N. benthamiana*, candidate interactors were designated as proteins with a linear fold change bait:citrine or bait:WT ≥ 1.7 and a false-discovery rate (*q*-value) ≤ 0.1. Arabidopsis orthologs for each DMR6/DLO1 candidate interactor were determined via protein sequence BLAST, and GO BP enrichment analysis was performed via the ClueGO Cytoscape^91^ plugin on Cytoscape v.10.0.1^92^. Terms with *p* < 0.05 were selected as enriched.

### LC–MS/MS detection and quantification of salicylic acid and 2,5-DHBA

To quantify SA and 2,5-DHBA *in planta*, ~100 mg of 15-day-old *Arabidopsis thaliana* whole seedlings were harvested for four independent experiments. Tissues were flash-frozen and extracted in 500 µL of 80% (v/v) Optima LC–MS-grade methanol with 0.1% (v/v) Optima formic acid (Thermo Fisher Scientific). Samples were vortexed briefly and homogenized using a bead mill (2 min at 25 Hz/s). Extracts were centrifuged, and 200 µL of the supernatant was collected and filtered through 0.2 µm PTFE filters prior to LC–MS analysis.

Liquid chromatography was performed on a Thermo Scientific Vanquish UHPLC equipped with an Acquity UPLC BEH C18 column (2.1 × 100 mm, 1.7 μm particle size; Waters). Mobile phases consisted of Optima LC–MS-grade water with 0.1% formic acid (eluent A) and acetonitrile with 0.1% formic acid (eluent B). The gradient was run at a flow rate of 0.6 mL min^−^^1^ as follows: −1 to 0 min, 1% B; 0 to 0.5 min, 40% B; 0.5 to 7.5 min, 80% B; 7.5 to 9 min, 80% B; 9.0 to 9.0 min, 1% B; and 9.0 to 10.0 min, 1% B. The column compartment was maintained at 40 °C. Mass spectrometry was performed using a Q Exactive Orbitrap mass spectrometer (Thermo Fisher Scientific) operating in parallel reaction monitoring (PRM) mode. The instrument was run in negative ionization mode (spray voltage, −4 kV; capillary temperature, 350 °C; auxiliary gas heater, 350 °C; sheath gas flow rate, 45; auxiliary gas flow rate, 15; sweep gas flow rate, 3). PRM parameters were: resolution 35,000 (at m/z 200); AGC target 2 × 10⁵; maximum injection time 200 ms; isolation window 1.0 m/z; NCE, stepped 35; microscans 1; default charge state 1; in-source CID 0 eV. The inclusion list targeted the following precursor ions: SA (m/z 137.0244; 1.50–1.60 min; NCE 38) and 2,5-DHBA (m/z 153.0193; 1.10–1.20 min; NCE 35). Chromatographic peak width was set to 15 s (FWHM). Data were collected in profile mode over a 0–10 min runtime. Quantification of SA and 2,5-DHBA was performed using external calibration curves. Authentic standards (Thermofisher #419225000 and #A11459.14) were prepared over a concentration range of 1 ×  10⁻⁵–0.1 mg mL⁻¹ and analyzed under identical PRM conditions. Peak areas were integrated and plotted against known concentrations to generate linear calibration curves (*R*² ≥ 0.99). Sample metabolite concentrations were interpolated directly from these curves using log–log regression. Raw data were visualized, and peaks were integrated using Thermo FreeStyle 1.8 software, and calculations were performed in Excel. Concentration values were normalized to tissue fresh weight to yield metabolite abundance per g fresh tissue (ug/g).

### Reporting summary

Further information on research design is available in the Nature Portfolio Reporting Summary linked to this article.

## Supplementary information

Supplementary Information

Description of Additional Supplementary Files

Supplementary Data 1

Supplementary Data 2

Supplementary Data 3

Supplementary Data 4

Supplementary Data 5

Supplementary Data 6

Supplementary Data 7

Supplementary Movie 1

Supplementary Movie 2

Supplementary Movie 3

Supplementary Movie 4

Reporting Summary

Transparent Peer Review File

## Source data

Source Data

## Data Availability

All data generated in this study are provided in the Supplementary Information, Supplementary Data, and Source data files. Source data are provided with this paper. The mass spectrometry proteomics data have been deposited to the ProteomeXchange Consortium via the PRIDE [1] partner repository with the dataset identifiers (PXD063820) for ASK1-*Pst* proximity labeling, and dataset identifiers (PXD063821) for DMR6 and DLO1 proximity labeling. All other related materials are available from the corresponding author upon request. Source data are provided with this paper.
