# Supplementary material for: Salicylic acid modulates its catabolic enzymes via proteasomal degradation linked to SCF-associated proximity networks
Source: Nat Commun. 2026 Apr 20;17:5468. doi: 10.1038/s41467-026-72241-x (PMC13284210; doi:10.1038/s41467-026-72241-x)
Supplement: Supplementary file 1 — Supplementary Information [file 41467_2026_72241_MOESM1_ESM.pdf]

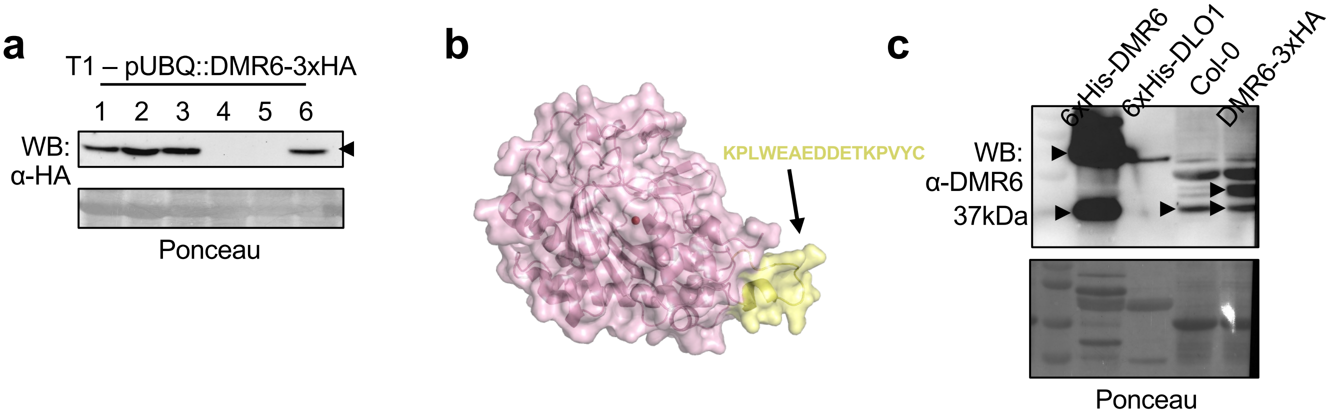

Supplementary Figure 1. DMR6 antisera characterization and isolation of stable DMR6-3xHA lines. . (**a**) anti-HA Western blot of T1 individuals following BASTA plate screening. (**b**) Visualization of DMR6 antisera antigen on the DMR6 molecular structure. (**c**) DMR6 antisera western blot verification. Left to right: Recombinant 6xHis-MysB-DMR6, lower arrow represents untagged DMR6, 6xHis-MysB-DLO1, Col-0 native extracts, pUBQ::DMR6-3xHA native extracts.

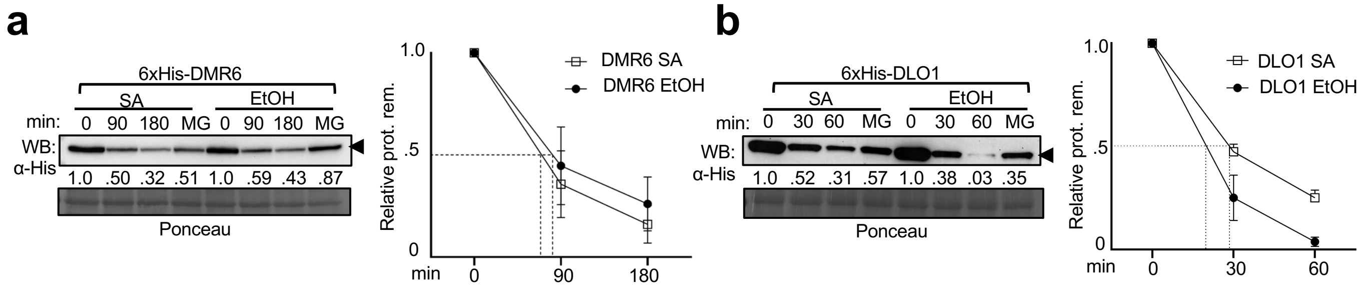

Supplementary Figure 2. Cell-free degradation assays with wild-type DMR6 and DLO1. (a, b) Western blots depicting cell-free degradation assays depicting proteasomal degradation of DMR6 (a) and DLO1 (b) under exogenous SA *in vitro*, n = 3 experiments, representative image shown.

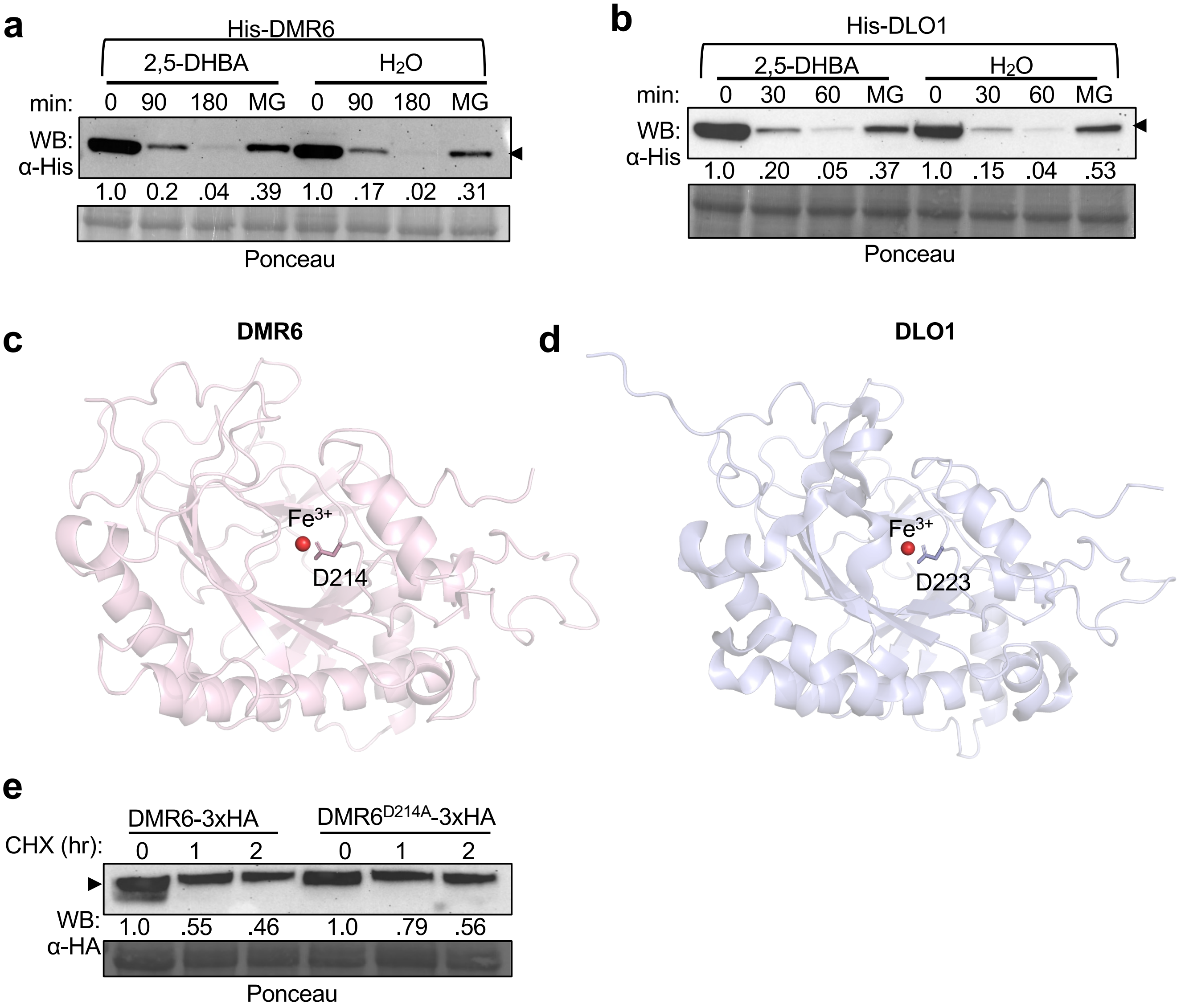

Supplementary Figure 3. DMR6/DLO1 cell-free degradation under 2,5-DHBA and catalytic mutant visualization. **(a, b)** DMR6 (a) and DLO1 (b) proteasomal degradation rates under exogenous 2,5-DHBA. N = 3 experiments, representative image shown. **(c, d)** Visualization of aspartic acid residues mutated to create DMR6 (c) and DLO1 (d) catalytic mutants. **(e)** *In planta* CHX chase assay of DMR6 and DMR6^{D214A}. N = 3 experiments, representative image shown.

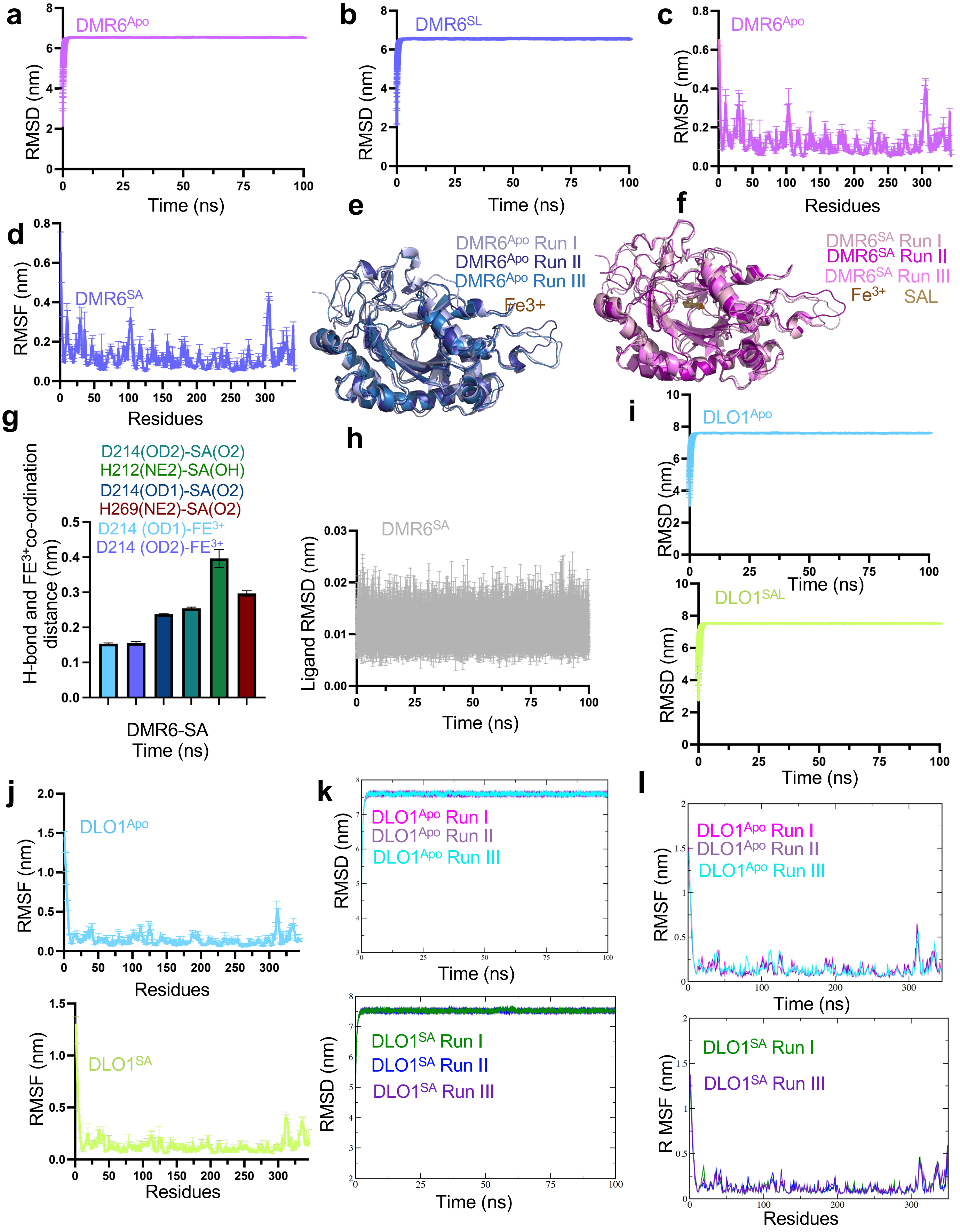

a
DMR6Apo
RMSD (nm)
Time (ns)
b
DMR6SL
RMSD (nm)
Time (ns)
c
DMR6Apo
RMSF (nm)
Residues
d
DMR6SA
RMSF (nm)
Residues
e
DMR6Apo Run I
DMR6Apo Run II
DMR6Apo Run III
Fe3+
f
DMR6SA Run I
DMR6SA Run II
DMR6SA Run III
Fe3+
SAL
g
D214(OD2)-SA(O2)
H212(NE2)-SA(OH)
D214(OD1)-SA(O2)
H269(NE2)-SA(O2)
D214 (OD1)-FE3+
D214 (OD2)-FE3+
H-bond and FE3+co-ordination distance (nm)
DMR6-SA
Time (ns)
h
DMR6SA
Ligand RMSD (nm)
Time (ns)
i
DLO1Apo
RMSD (nm)
Time (ns)
DLO1SAL
RMSD (nm)
Time (ns)
j
DLO1Apo
RMSF (nm)
Residues
DLO1SA
RMSF (nm)
Residues
k
DLO1Apo Run I
DLO1Apo Run II
DLO1Apo Run III
RMSD (nm)
Time (ns)
DLO1SA Run I
DLO1SA Run II
DLO1SA Run III
RMSD (nm)
Time (ns)
l
DLO1Apo Run I
DLO1Apo Run II
DLO1Apo Run III
RMSF (nm)
Time (ns)
DLO1SA Run I
DLO1SA Run III
R MSF (nm)
Residues

Supplementary Figure 4. Molecular dynamics analysis of DMR6 and DLO1 in *apo* and SA-bound states. (a–b) RMSD profiles of $DMR6^{apo}$ (*pink*) and $DMR6^{SA}$ (*purple*) showing rapid equilibration within the first 10 ns and stable trajectories over 100 ns. Error bars represent the standard deviation (SD) across three independent replicates. **(c–d)** RMSD profiles of *$DLO1^{apo}$ (pink)* and $DLO1^{SA}$, (*purple*) showing similar backbone stability and reproducibility across triplicate runs, with SD indicated by error bars. **(e–f)** Representative snapshots from triplicate simulations of $DMR6^{apo}$ and $DMR6^{SA}$ (Run I-lightpink, Run II-magenta; Run III-pink) highlighting consistent overall conformational with Fe^{3+} (sphere, orange) and SA (sticks, light-orange) positioning within the catalytic pocket. **(g)** Hydrogen-bond and Fe^{3+}coordination distances for $DMR6^{SA}$, showing persistent interactions between catalytic residues (D214, H212) and SA (O2, OH) together with Fe^{3+}. **(h)** Ligand RMSD plot (mean ± SD) for $DMR6^{SA}$ demonstrating that SA remained tightly bound within the active site with minimal deviation throughout the 100-ns simulation. (**i**) Average RMSD profiles of $DLO1^{apo}$ (right-up, *cyan*) and $DLO1^{SA}$ (right-bottom, *green*) across triplicates trajectories. error bars represent SD across three replicates. Bar indicates the SD. (**j**) Average RMSF profiles of $DLO1^{apo}$ (left-up, *cyan*) and $DLO1^{SA}$ (left-bottom, *green*) across triplicates trajectories. error bars represent SD across three replicates. Bar indicates the SD. (**k**) Superposition of RMSD profile of $DLO1^{apo}$ (*up*, Run I- magenta; Run II-brown; Run III-Cyan) and $DLO1^{SA}$ (*down,* Run I-green, Run II-blue; Run III-purple) in triplicates showing highly consistent equilibration and stable backbone trajectories. (**l**) Superposition of RMSF profile of $DLO1^{apo}$ (*up*, Run I- magenta; Run II-brown; Run III-Cyan) and $DLO1^{SA}$ (*down,* Run I-green, Run II-blue; Run III-purple) in triplicates showing highly consistent equilibration and stable backbone trajectories.

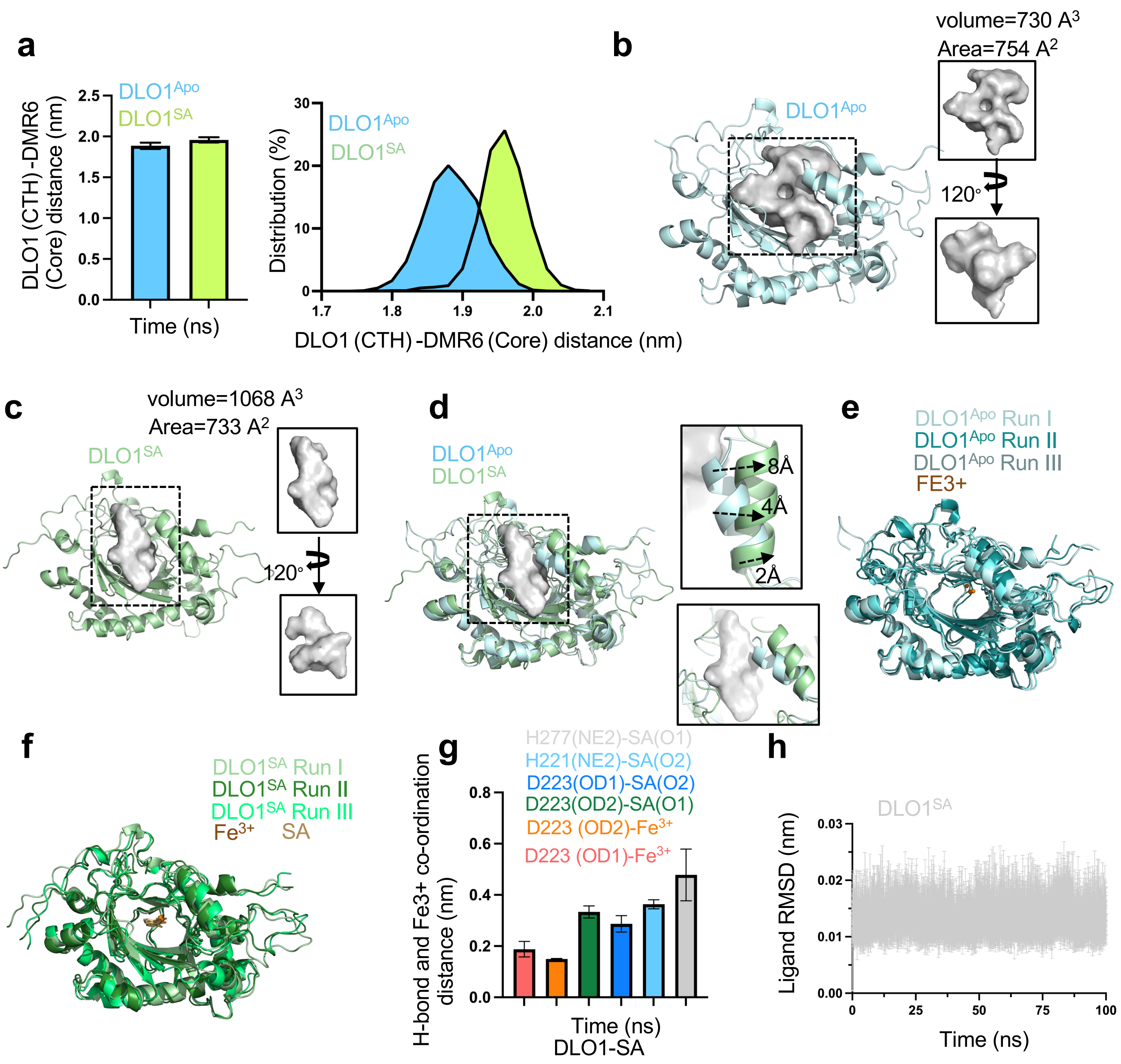
a
DLO1 (CTH) -DMR6 (Core) distance (nm)
2.5
2.0
1.5
1.0
0.5
0.0
DLO1Apo
DLO1SA
Time (ns)
Distribution (%)
30
20
10
0
1.7
1.8
1.9
2.0
2.1
DLO1 (CTH) -DMR6 (Core) distance (nm)
b
volume=730 A3
Area=754 A2
DLO1Apo
120°
c
volume=1068 A3
Area=733 A2
DLO1SA
120°
d
DLO1Apo
DLO1SA
8Å
4Å
2Å
e
DLO1Apo Run I
DLO1Apo Run II
DLO1Apo Run III
FE3+
f
DLO1SA Run I
DLO1SA Run II
DLO1SA Run III
Fe3+
SA
g
H-bond and Fe3+ co-ordination distance (nm)
H277(NE2)-SA(O1)
H221(NE2)-SA(O2)
D223(OD1)-SA(O2)
D223(OD2)-SA(O1)
D223 (OD2)-Fe3+
D223 (OD1)-Fe3+
0.8
0.6
0.4
0.2
0.0
Time (ns)
DLO1-SA
h
DLO1SA
Ligand RMSD (nm)
0.03
0.02
0.01
0.00
0
25
50
75
100
Time (ns)

Supplementary Figure 5. Molecular dynamics analysis of C-terminal Helix (CTH) in DLO1^{apo} and DLO1^{SA} bound states. (a) Quantitative analysis of the DLO1-CTH(323-332)–DLO1-Core(1-322) distance across triplicates, showing a reproducible ligand-induced outward shift in DLO1^{SA} relative to DLO1^{apo}, corresponding to the transition from an open to a closed catalytic pocket. **(b-c)** Representative snapshots of DLO1^{apo} (*cyan*) and DLO1^{SA} (*green*) depicting changes in catalytic pocket surface area (from 754 Å^2 to 733 Å^2) and volume (from 730 Å3 to 1068 Å3) measured using KVFinder. **(d)** Structural Superposition of of DLO1^{apo} (*cyan*) and DLO1^{SA} (*green*) states depicting 2-8 Å outward shift upon SA binding . (left-up), Close up views of CTH between DLO1^{apo} (*cyan*) and DLO1^{SA} (*pale-green*) illustrating the transition from a closed to open state. (left-down), Close-up view of the CTH corresponding to the pocket volume calculated using KVfinder. **(e–f)** Representative triplicate snapshots of DLO1^{apo} (Run I-cyan, Run II-blue and Run III-palecyan)and *DLO1^{SA}* (Run I-palegreen, Run II-forest green and Run III-lightgreen) depicting conformational differences in CTH positioning and Fe^{3+} (sphere, orange) coordination and SA (sticks, light-orange) within the catalytic site. **(g)** Hydrogen-bond and Fe^{3+}coordination distances for DLO1^{SA}, showing stable interactions between catalytic residues (D223, H221) and SA (O1, O2). Error bar indicates SD across three replicates. **(h)** Ligand RMSD plot (mean ± SD) for DLO1^{SA} confirming that SA maintained a stable binding pose within the catalytic pocket across all three simulations.

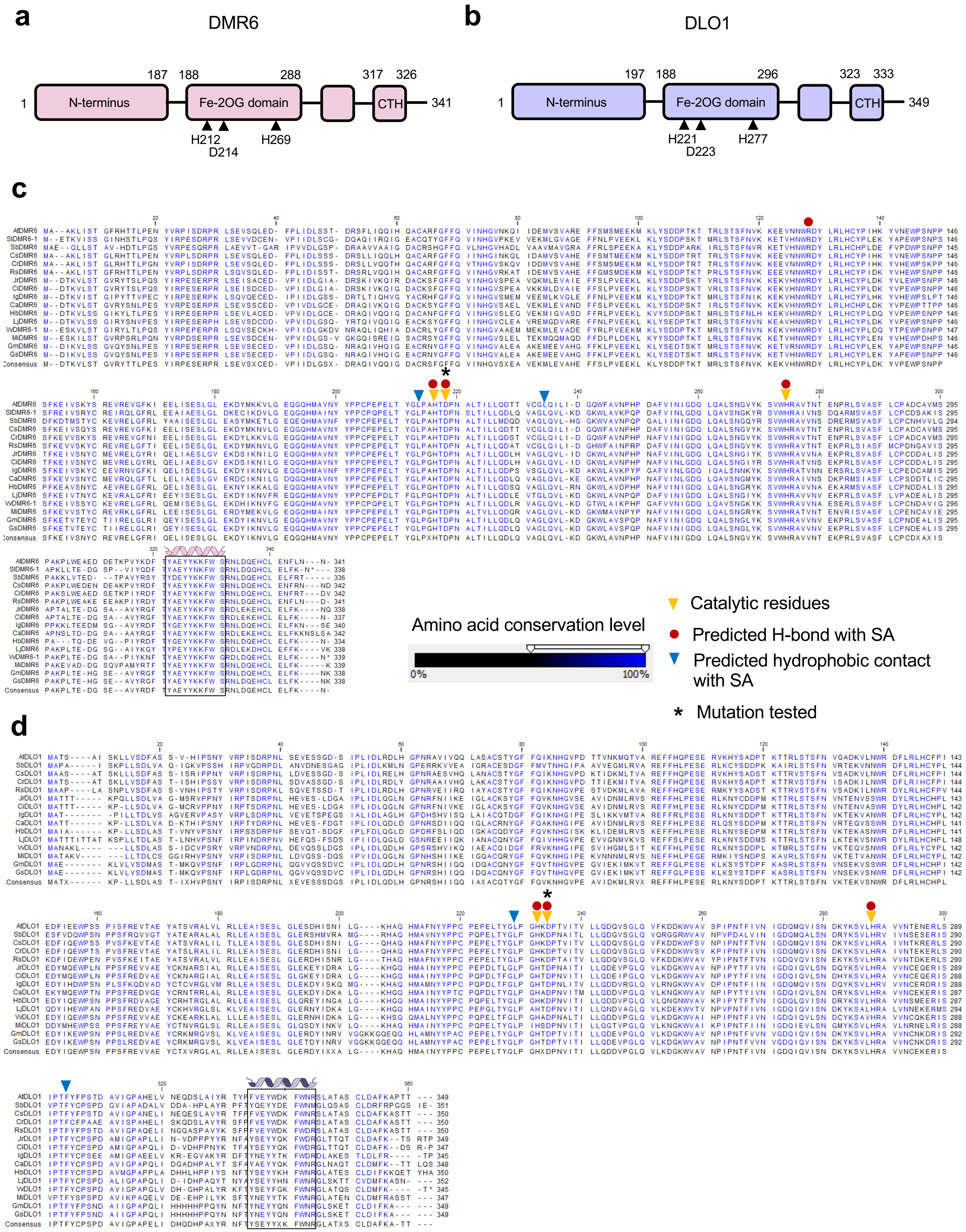
a
DMR6
1
N-terminus
187
188
Fe-2OG domain
288
317
326
CTH
341
H212
D214
H269
b
DLO1
1
N-terminus
197
188
Fe-2OG domain
296
323
333
CTH
349
H221
D223
H277
c
d
Amino acid conservation level
0%
100%
Catalytic residues
Predicted H-bond with SA
Predicted hydrophobic contact with SA
* Mutation tested

Supplementary Figure 6. Sequence analysis of DMR6 and DLO1. (a,b) Cartoon schematic of DMR6 **(a)** and DLO1 **(b)** putative functional domains and catalytic residues. **(c,d)** Protein multiple sequence alignments of DMR6 (c) and DLO1 (d) along with putative orthologs identified via NCBI BLASTp and aligned and visualized using CLC Genomics Workbench v22. Amino acids are colored by conservation level, where bright blue residues are more conserved. Yellow triangles indicate previously described catalytic residues. Blue triangles indicate residues with predicted hydrophobic contacts with SA per IFD, red circles indicate residues predicted to H-bond with SA per IFD, and black asterisks indicate tested amino acid point mutants. Naming scheme: At = *Arabidopsis thaliana*, Sl = *Solanum lycopersicum*, Sb = *Sorghum bicolor*, Cs = *Camelina sativa*, Cr = *Capsella rubella*, Rs = *Raphanus sativus*, Jr = *Juglans regia*, Ci = *Carya illinoinensis*, Ig = *Impatiens glandulifera*, Ca = *Corylus avellana*, Hb = *Hevea brasiliensis*, Lj = *Lotus japonicus*, Vv = *Vitis vinifera*, Mi = *Macadamia integrifolia*, Gm = *Glycine max,* Gs = *Glycine soja*

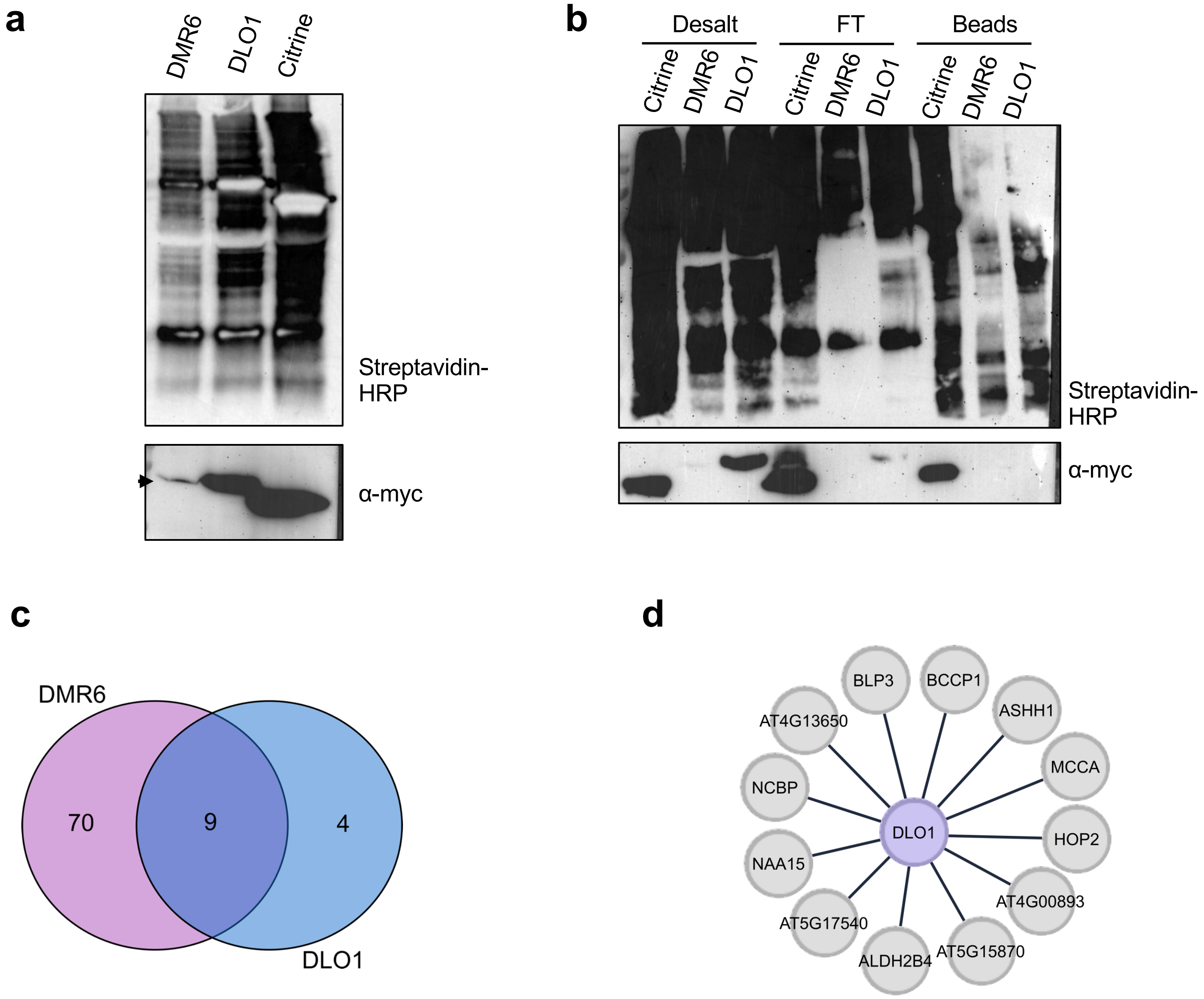

Supplementary figure 7. DMR6 and DLO1 proximity labeling. (a) Anti-myc and streptavidin-HRP western blots on crude total protein extracts from *N. benthamiana* plants done to confirm transient expression of DMR6, DLO1, and Citrine TurboID baits and biotinylation activity. These tissues were subsequently used for extraction of biotinylated proteins and LC/MS-MS analysis. **(b)** Western blot depicting the purification process of biotinylated proteins from total cell extracts from N. benthamiana transiently expressing DMR6, DLO1, or Citrine TurboID fusions. Desalt = total protein extract following desalting to remove free biotin, FT = flowthrough sample of proteins remaining in extract following incubation with streptavidin beads, beads = proteins bound to streptavidin beads. **(c)** Venn diagram depicting number of overlapping and unique candidate DMR6 and DLO1 interactors. **(d)** Protein interaction network of candidate DLO1 interactors.

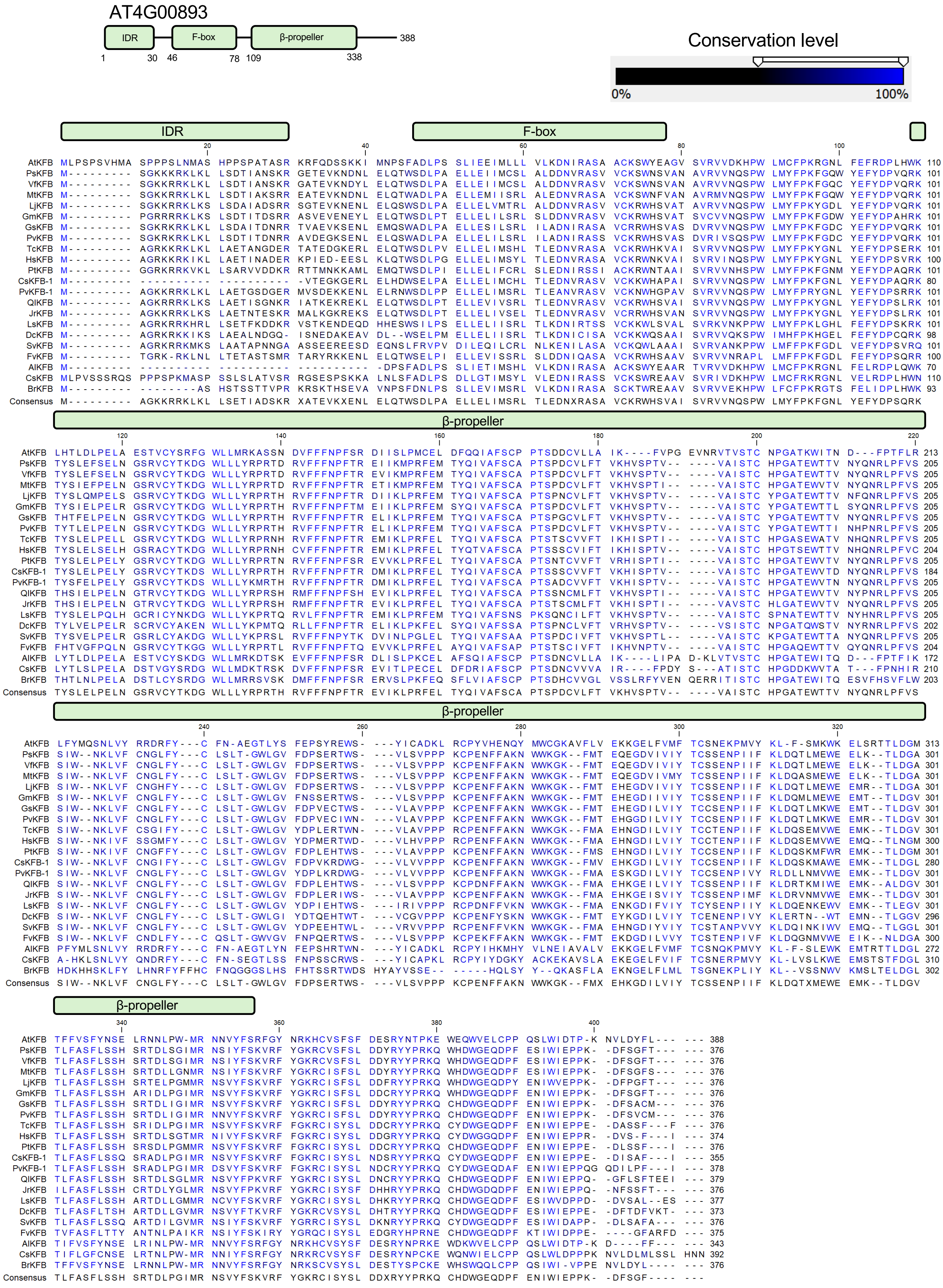
AT4G00893
IDR
F-box
β-propeller
1
30
46
78
109
338
388
Conservation level
0%
100%
AtKFB
PsKFB
VfKFB
MtKFB
LjKFB
GmKFB
GsKFB
PvKFB
TcKFB
HsKFB
PtKFB
CsKFB-1
PvKFB-1
QlKFB
JrKFB
LsKFB
DcKFB
SvKFB
FvKFB
AlKFB
CsKFB
BrKFB
Consensus

Supplementary Figure 8. DAF1/AT4G00893 protein sequence analysis. Protein multiple sequence alignments of putative DMR6-regulating Kelch F-box protein (KFB) or AT4G00893, along with putative orthologs identified via NCBI BLASTp and aligned and visualized using CLC Genomics Workbench v22. Amino acids are colored by conservation level, where bright blue residues are more conserved. Naming scheme: At = *Arabidopsis thaliana*, Ps = *Pisum sativum*, Vf = *Vicia faba*, Mt = *Medicago truncatulata*, Lj = *Lotus japonicus*, Gm = *Glycine max*, Gs = *Glycine soja*, Pv = *Phaseolus vulgaris*, Tc = *Theobroma cacao*, Hs = *Hibiscus syriacus*, Pt = *Populus trichocarpa*, Cs = *Citrus sinensis*, Pv = *Pistacia vera*, Ql = *Quercus lobata*, Jr = *Juglans regia*, Ls = *Lactuca sativa*, Dc = *Dacusu carota* subsp. *sativus*, Sv = *Solanum verrucosum*, Fv = *Fragaria vesca* subsp. *vesca*, Al = *Arabidopsis lyrata*, Cs = *Camelina sativa*, Br = *Brassica rapa*

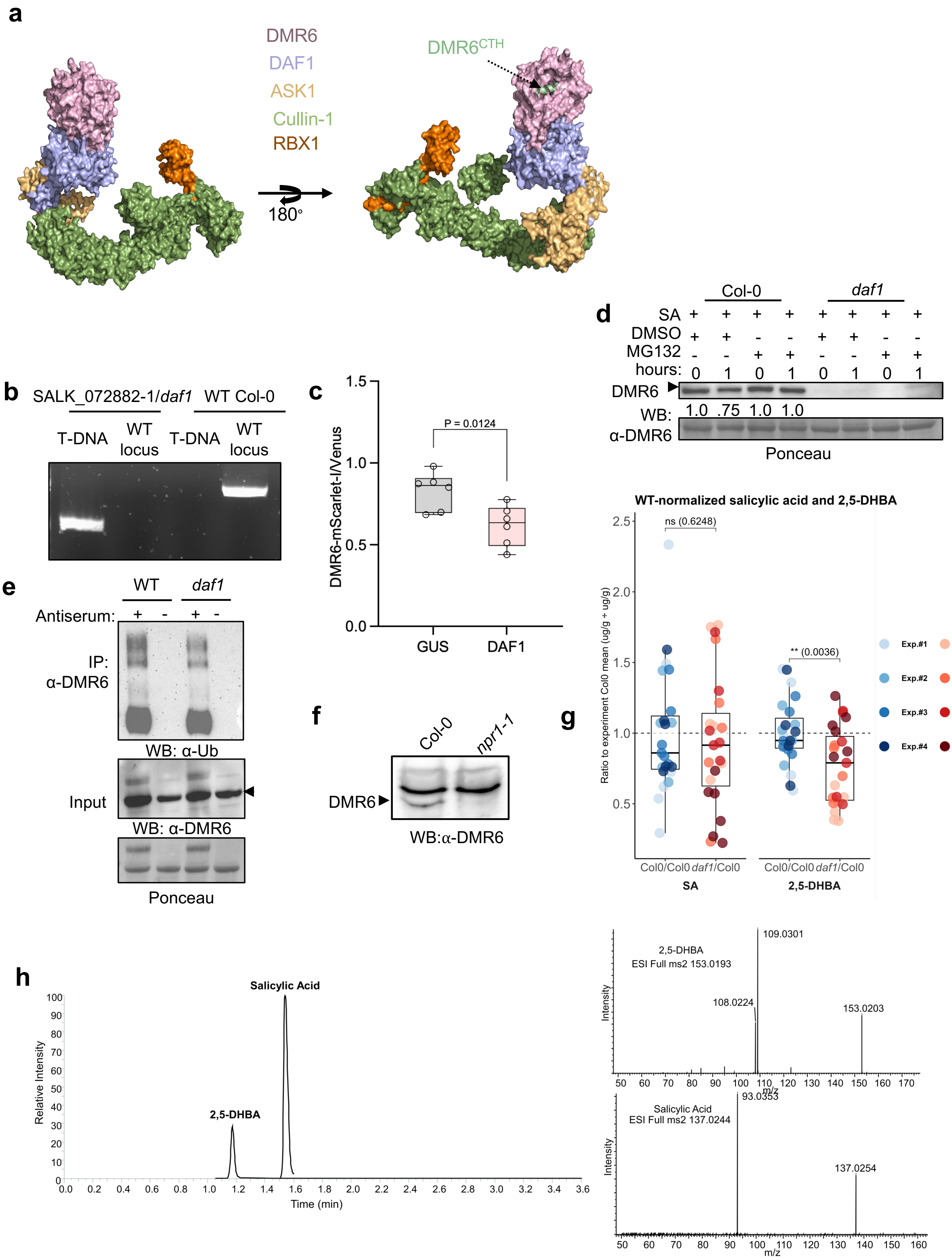
a
DMR6
DAF1
ASK1
Cullin-1
RBX1
DMR6CTH
180°
b
SALK_072882-1/daf1
WT Col-0
T-DNA
WT locus
T-DNA
WT locus
c
DMR6-mScarlet-I/Venus
P = 0.0124
1.5
1.0
0.5
0.0
GUS
DAF1
d
Col-0
daf1
SA + + + + + + + +
DMSO + + - - + + - -
MG132 - - + + - - + +
hours: 0 1 0 1 0 1 0 1
DMR6
WB: 1.0 .75 1.0 1.0
α-DMR6
Ponceau
e
WT
daf1
Antiserum: + - + -
IP: α-DMR6
WB: α-Ub
Input
WB: α-DMR6
Ponceau
f
Col-0
npr1-1
DMR6
WB:α-DMR6
g
WT-normalized salicylic acid and 2,5-DHBA
Ratio to experiment Col0 mean (ug/g ÷ ug/g)
ns (0.6248)
** (0.0036)
2.5
2.0
1.5
1.0
0.5
Col0/Col0 daf1/Col0
Col0/Col0 daf1/Col0
SA
2,5-DHBA
Exp.#1
Exp.#2
Exp.#3
Exp.#4
h
Salicylic Acid
2,5-DHBA
Relative Intensity
100
90
80
70
60
50
40
30
20
10
0
0.0 0.2 0.4 0.6 0.8 1.0 1.2 1.4 1.6 1.8 2.0 2.2 2.4 2.6 2.8 3.0 3.2 3.4 3.6
Time (min)
2,5-DHBA
ESI Full ms2 153.0193
109.0301
108.0224
153.0203
Intensity
50 60 70 80 90 100 110 120 130 140 150 160 170
m/z
Salicylic Acid
ESI Full ms2 137.0244
93.0353
137.0254
Intensity
50 60 70 80 90 100 110 120 130 140 150 160
m/z

Supplementary Figure 9. Biological characterization of the *daf1* mutant. (a) Surface presentation of AlphaFold 3 (AF3) structural prediction modeling the putative SCFDAF1 E3 ligase complex. Color scheme is as follows: Cullin1 (forest-green), RBX1 (orange), ASK1 (yellow), DAF1 (lavender), DMR6 (pink), DMR6-CTH (lightgreen). The ASK1-DMR6-DAF1 model was aligned to the SCF-Cullin-1 reference structure (PDB ID:6TTU). **(b)** Genotyping PCR depicting amplification of WT DMR6 and T-DNA specific PCR products from WT Col-0 and T-DNA insertion mutant SALK_072882-1, hereafter referred to as *daf1*. **(c)** Box plot depicting ratio of fluorescent signal of reporter protein DMR6 relative to fluorescent reference protein VENUS when co-expressed with GUS (negative control, grey) or DAF1 (pink). N = 6 biological replicates.**(d)** Western blot depicting relative endogenous DMR6 levels in Col-0 vs *daf1* T-DNA mutants treated with SA and DMSO and/or MG132. **(e)** Western blot depicting an immunoprecipitation assay of DMR6 pulled down from native extracts of wild-type Col-0 (WT) and *daf1* seedlings using the custom DMR6 antiserum and enriched with magnetic Pierce Protein A/G beads. Empty lanes indicate control samples of magnetic beads incubated only with extract and no DMR6 antiserum. **(f)** Western blot depicting relative endogenous DMR6 levels in Col-0 and *npr1-1* seedlings **(g)** Relative abundance of salicylic acid (SA) and 2,5-DHBA in *daf1* and Col-0 across four independent experiments. For each sample, metabolite levels were normalized to the mean of the corresponding Col-0 group within each experiment. Each genotype had n = 6 biological replicates per experiment (n = 4). Data are shown as boxplots with individual points overlaid. Statistical significance was assessed using two-tailed Student's *t*-tests comparing Col-0 and *daf1* for each metabolite. **(h)** LC-MS/MS chromatogram for SA and 2,5-DHBA and mass spectra(MS2) for SA and 2,5-DHBA.

a

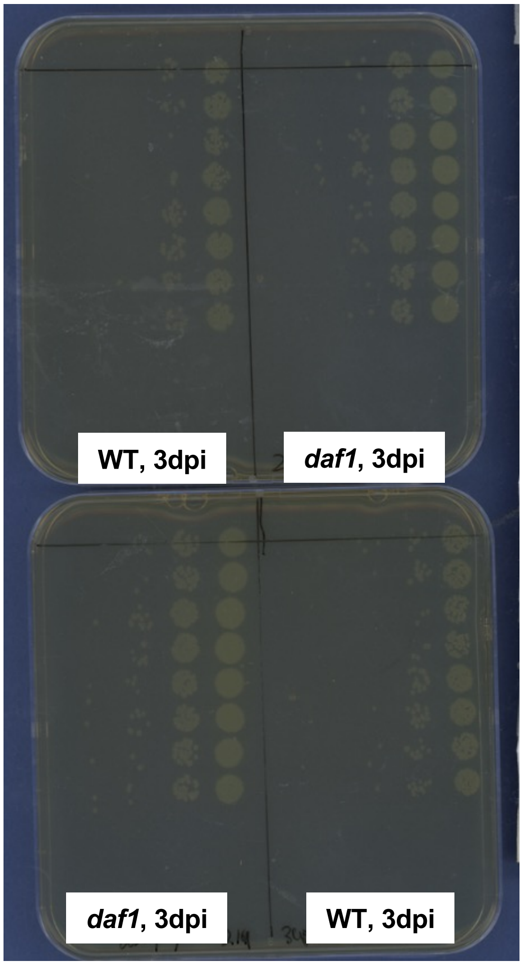

Supplementary Figure 10. *Pst* infection assays on Col-0 and *daf1*. Representative scans of plates harboring recovered *Pst* colonies from Col-0 and *daf1* at 3dpi. N = 3 independent experiments.

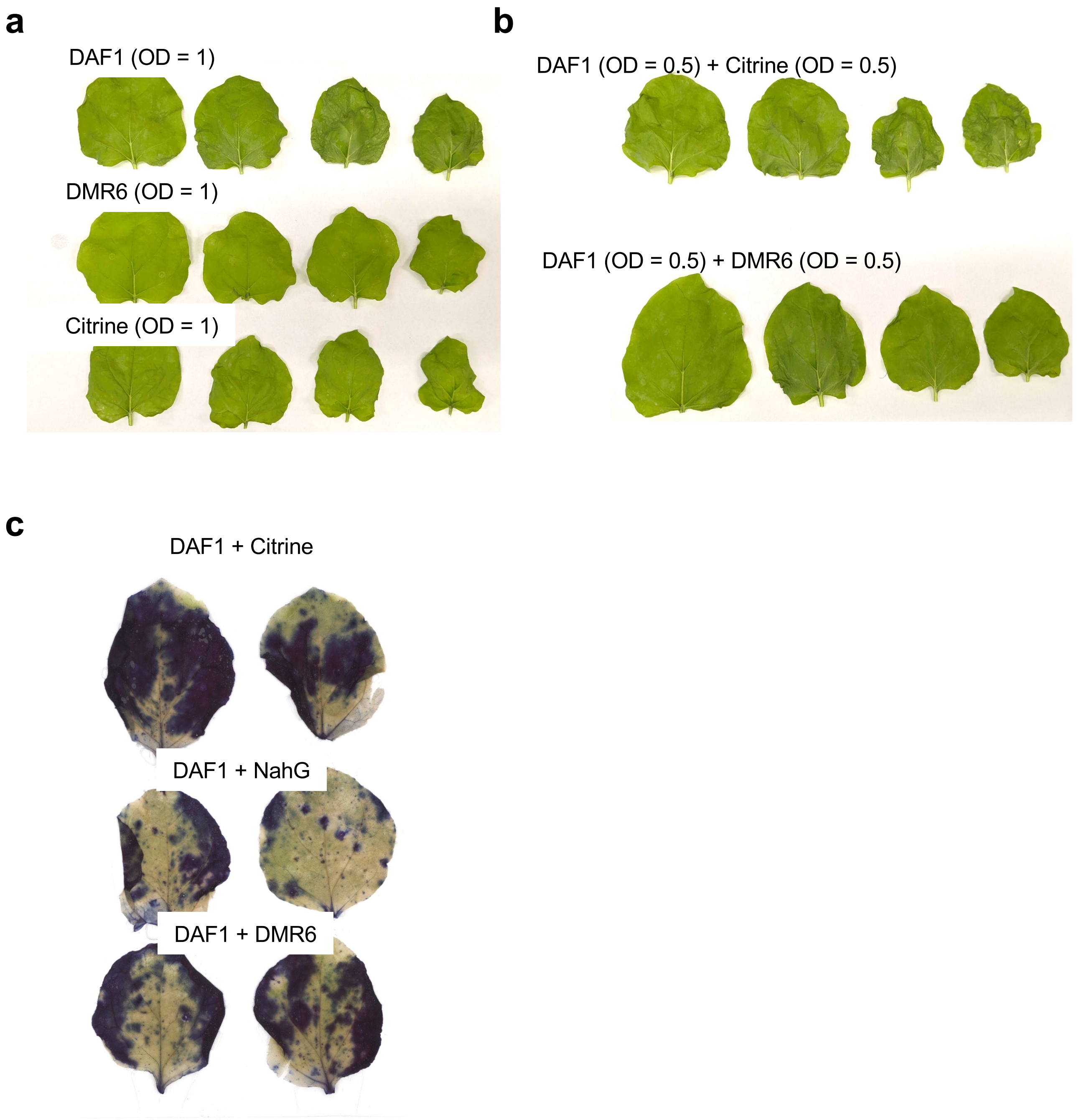

Supplementary Figure 11. Characterizing interplay between DMR6 and DAF1 and contributions to cell death. (a) Representative scans of *N. benthamiana* leaves individually expressing DAF1, DMR6, or Citrine via Agrobacterium-mediated transient transformation, n = 3 experiments. **(b)** Representative scans of *N. benthamiana* leaves transiently co-expressing DAF1 + citrine or DAF1 + DMR6, corresponding scans of trypan-blue stained leaves shown in Figure 4l, n = 3 experiments. **(c)** Representative trypan-blue stained *N. benthamiana* leaves transiently co-expressing DAF1 and Citrine, NahG, or DMR6, n = 3 experiments.

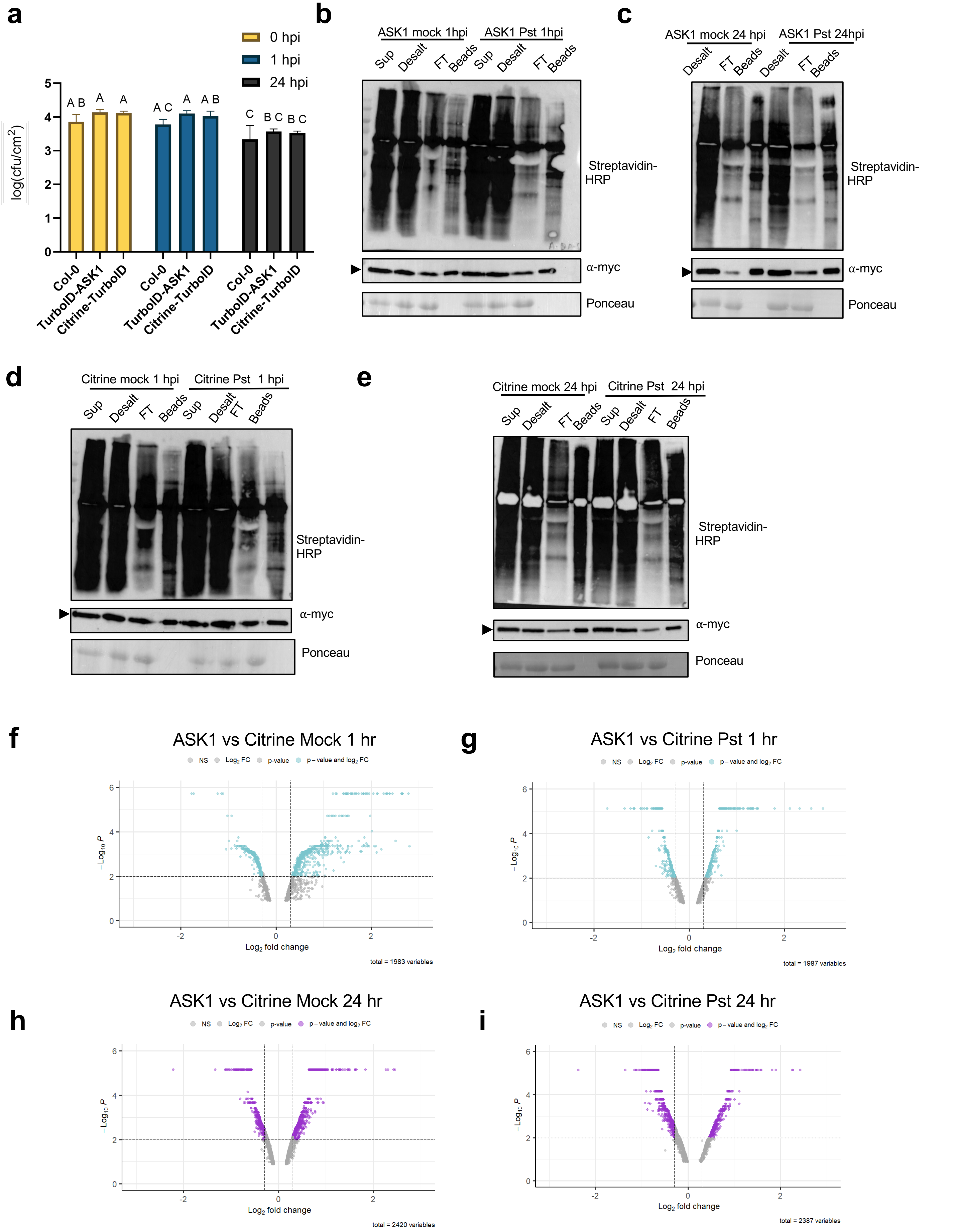
a
0 hpi
1 hpi
24 hpi
log(cfu/cm2)
A B
A
A
A C
A
A B
C
B C
B C
Col-0
TurboID-ASK1
Citrine-TurboID
b
ASK1 mock 1hpi
ASK1 Pst 1hpi
Sup
Desalt
FT
Beads
Streptavidin-HRP
α-myc
Ponceau
c
ASK1 mock 24 hpi
ASK1 Pst 24hpi
d
Citrine mock 1 hpi
Citrine Pst 1 hpi
e
Citrine mock 24 hpi
Citrine Pst 24 hpi
f
ASK1 vs Citrine Mock 1 hr
NS
Log2 FC
p-value
p – value and log2 FC
Log2 fold change
total = 1983 variables
g
ASK1 vs Citrine Pst 1 hr
total = 1987 variables
h
ASK1 vs Citrine Mock 24 hr
total = 2420 variables
i
ASK1 vs Citrine Pst 24 hr
total = 2387 variables

Supplementary Figure 12. Isolation of putative ASK1 interactors during *Pst* DC3000 infection. **(a)** *Pst* DC3000 bacterial recovery assay from Col-0 and TurboID genotypes at 0, 1, and 24hpi. **(b-e)** Western blot analysis depicting the purification process of biotinylated proteins from TurboID stable transgenic lines subjected to either *Pst* infection or mock treatment. **(f-i)** Volcano plots depicting proteins identified in Mock 1 hr (f), *Pst* DC3000 1 hour (g), Mock 24 hour (h), *Pst* DC3000 24 hour (i) PL experiments. Colored dots indicate proteins with fold change ASK1:Citrine ≥ 1.23 and *q*-value ≤ 0.1.

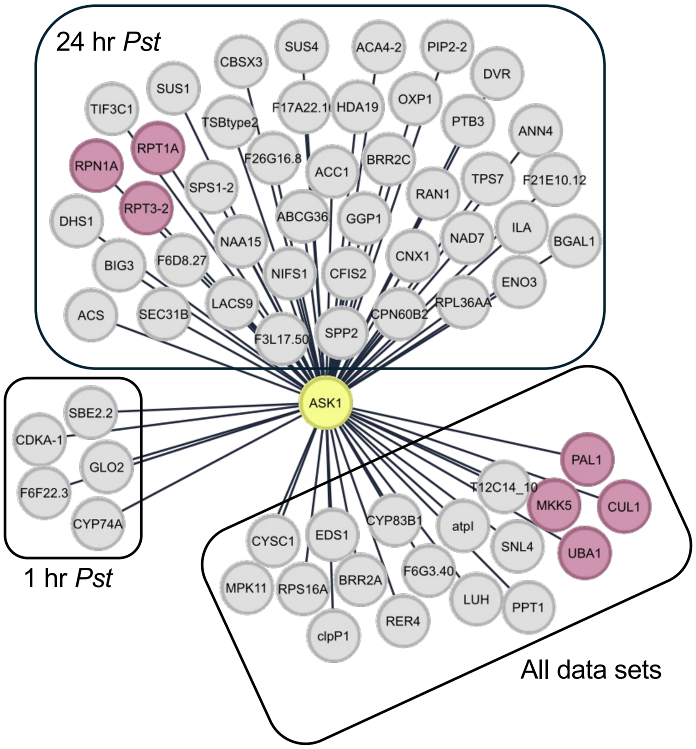

Supplementary Figure 13. Previously identified ubiquitinated proteins among ASK1 interactors. Protein interaction network depicting putative ASK1 interactors previously identified in the flg22-associated ubiquitylome generated in Ma et al 2021. 24 hr *Pst* = proteins unique to the 24 hr Pst data set, 1 hr *Pst* = proteins unique to the 1 hr *Pst* data set, All data sets = Proteins found across all 4 data sets. Red-colored nodes indicate proteins with a previously known association to the UPS.

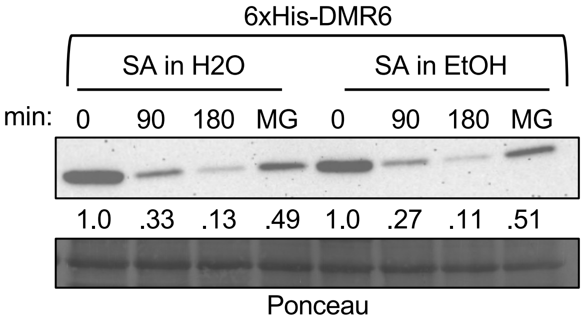

Supplementary Figure 14. Comparison of DMR6 stability in cell-free systems with EtOH vs H2O as SA solvent. Western blot depicting cell-free degradation assay of recombinant DMR6 protein incubated with native protein extract and exogenous SA dissolved in either H2O (left lanes) or EtOH as the solvent (right lanes). Ponceau serves as the loading control.

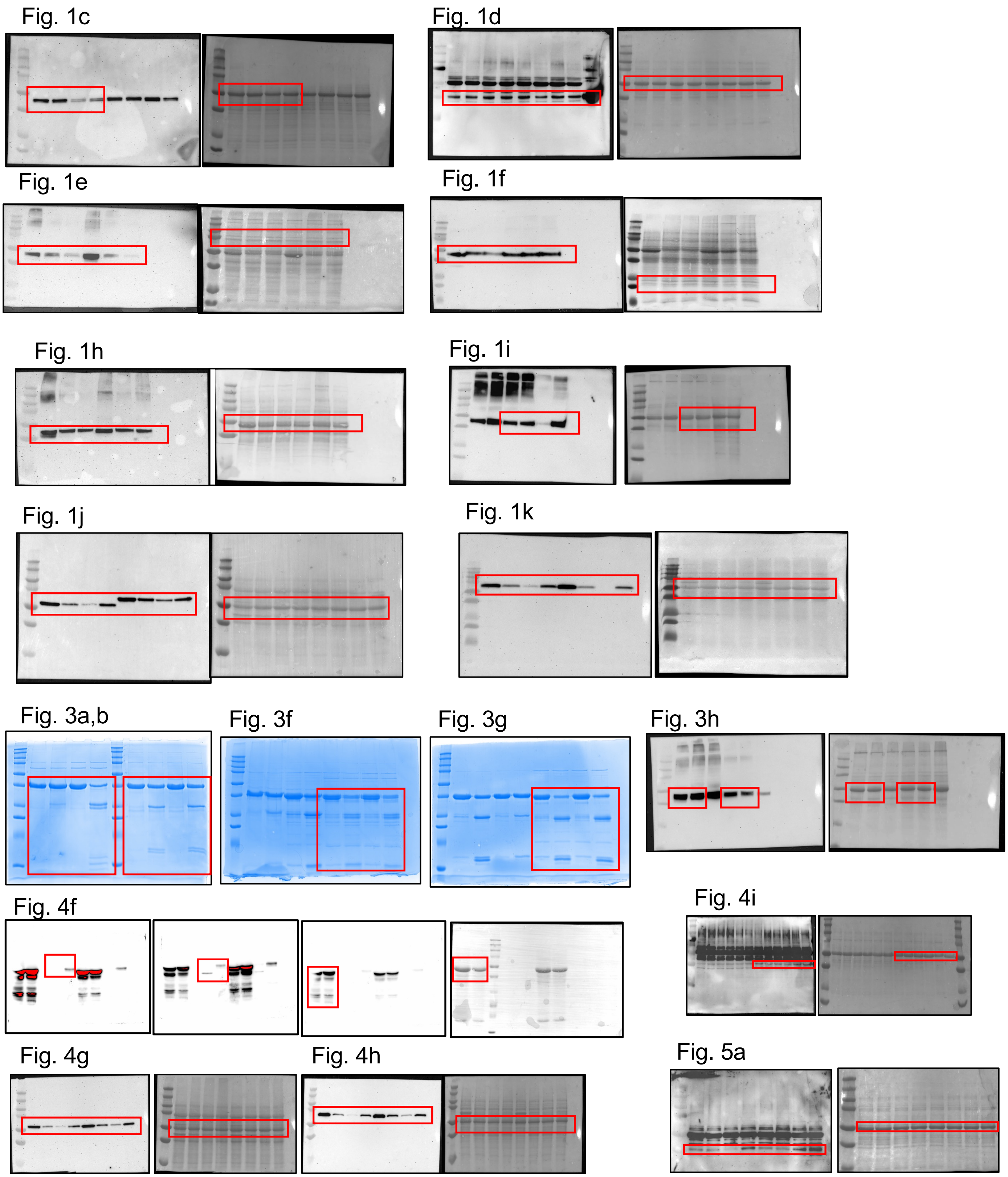

Supplementary Figure 15. Uncropped gel images from main figures. Uncropped gel images from main figures. The red box indicates the gel portion used for figures.

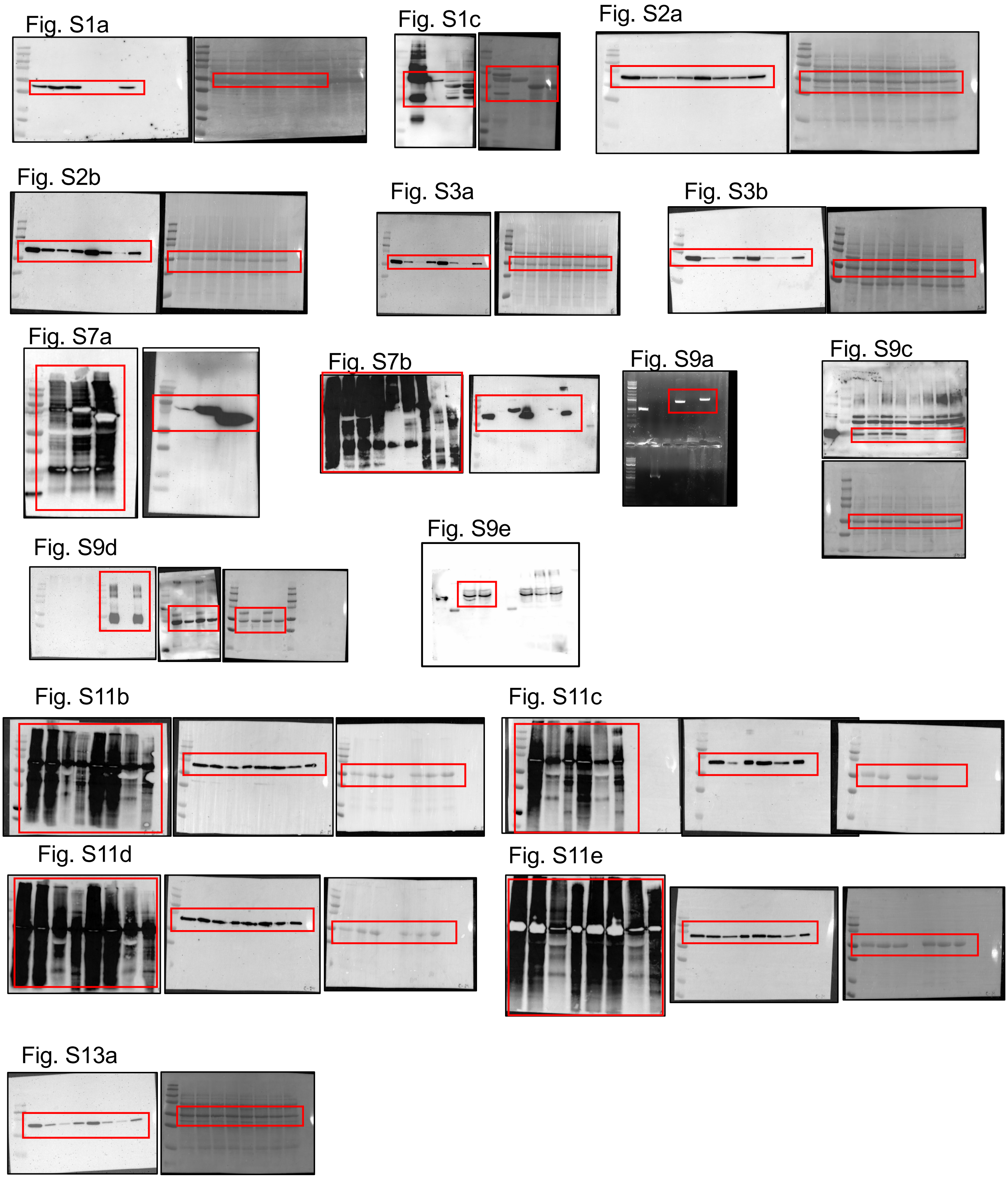

Supplementary figure 16. Uncropped gel images from supplementary figures. Uncropped gel images from main figures. The red box indicates the gel portion used for figures.

Supplementary Table 1. Molecular dynamics simulation summary

Parameter / analysis	Description
Equilibration and convergence	Structural stability assessed using RMSD and RMSF across trajectories. All systems equilibrated within the first ~10 ns and remained stable thereafter, with low variance across replicates indicating convergence (Fig. 2a–d; Supplementary Fig. 4)
Conformational dynamics	Inter-domain distance between CTH-Core of DMR6/DLO1 in both apo and SA bound states were monitored over equilibrated trajectories, revealing ligand induced compaction and conformational transitions (Fig. 2e-h; Supplementary Fig. 5)
Simulation design	Systems were energy minimized, followed by 1 ns NVT and 1 ns NPT equilibration with positional restraints, and 100 ns unrestrained production runs for each system (Methods)
Replicates	Three independent simulations per condition (apo and SA-bound), initialized with different random velocity seeds; analyses performed independently without pooling prior to statistical representation (Fig. 2; Supplementary Fig. 4-5)
Structure-function relationship	Pocket volume and surface area changes correlate with protein stability in vitro and in planta (Fig. 2f-h; Supplementary Fig. 5 and Supplementary movie 1-4)
Force field and solvent	Simulations performed using the CHARMM36m force field for proteins and ions, with the TIP3P explicit water model; systems treated under periodic boundary conditions (Methods)
System setup	Protein placed in a cubic simulation box (~110 × 110 × 110 Å) with ~10-12 Å padding from box edges, solvated with explicit water, neutralized, and supplemented with 150 mM NaCl to mimic physiological ionic strength (Methods)
Simulation parameters	Long-range electrostatics calculated using Particle Mesh Ewald (PME); van der Waals interactions applied with a cutoff (~1.0-1.2 nm); all bonds involving hydrogen constrained using LINCS; 2 fs integration time step; temperature maintained at 300 K using a velocity-rescale thermostat and pressure at 1 bar using a Parrinello-Rahman barostat (Methods)
Software	Simulations carried out using GROMACS v2023.3; trajectory analysis performed using built-in GROMACS tools, Xmgrace, and GraphPad Prism for visualization and statistical analysis (Methods)
Data availability	Input parameter files and representative trajectories are available upon request

Summary of key parameters, validation metrics, and reproducibility criteria for molecular dynamics simulations of DMR6 and DLO1 in apo and SA bound states. Metrics correspond to analyses presented in Fig. 2 and Supplementary Figs. 4–5 and Supplementary movie 1-4.
