# Supplementary material for: Salicylic acid modulates its catabolic enzymes via proteasomal degradation linked to SCF-associated proximity networks
Source: Nat Commun. 2026 Apr 20;17:5468. doi: 10.1038/s41467-026-72241-x (PMC13284210; doi:10.1038/s41467-026-72241-x)
Supplement: Supplementary file 2 — Description of Additional Supplementary Files [file 41467_2026_72241_MOESM2_ESM.pdf]

Description of Additional Supplementary Files

File name: **Supplementary Data 1. DMR6 and DLO1 proximity labeling datasets.**
Description: Comprehensive list of proteins identified by TurboID-based proximity labeling of DMR6 and DLO1 expressed in *Nicotiana benthamiana*. The dataset includes protein identifiers, quantitative enrichment relative to Citrine control samples, and associated statistical values used for filtering candidate interactors.

File name: **Supplementary Data 2. ASK1 proximity labeling datasets under mock and Pst DC3000 conditions.**
Description: Proteomic datasets obtained from TurboID-ASK1 experiments under mock treatment and *Pseudomonas syringae* pv. *tomato* DC3000 infection. Includes protein identifiers, quantitative measurements across biological replicates, and statistical analyses.

File name: **Supplementary Data 3. Comparative analysis of ASK1 proximity labeling datasets.**
Description: Dataset summarizing overlap and differential enrichment of ASK1-associated proteins across mock and infection conditions. Includes comparative metrics used to identify condition-specific and shared interactors.

File name: **Supplementary Data 4. Gene ontology enrichment analysis of ASK1-associated proteins.**
Description: PANTHER GO Biological Process enrichment analysis of proteins identified in ASK1 proximity labeling experiments. Includes enrichment scores, statistical significance, and associated GO terms.

File name: **Supplementary Data 5. ASK1-associated proteins linked to ubiquitin signaling and plant immunity.**
Description: Curated subset of ASK1 proximity labeling datasets highlighting proteins previously associated with ubiquitin-mediated processes and plant immune responses.

File name: **Supplementary Data 6. F-box proteins identified in ASK1 proximity labeling experiments.**

Description: List of F-box proteins detected in ASK1 TurboID datasets, including annotations and enrichment values across experimental conditions.

File name: **Supplementary Data 7. Primer sequences used in this study.**

Description: List of oligonucleotide sequences used for cloning, mutagenesis, and genotyping, including corresponding gene targets and applications.

File name: **Supplementary Movie 1**

Description: Molecular dynamics trajectory of $DMR6^{apo}$ state, illustrating intrinsic conformational flexibility of the catalytic pocket and surrounding regions in the absence of salicylic acid (SA)

File name: **Supplementary Movie 2**

Description: Molecular dynamics trajectory of $DMR6^{SA}$ bound state, showing ligand-induced stabilization and inward movement of the catalytic pocket relative to the $DMR6^{apo}$ form.

File name: **Supplementary Movie 3**

Description: Molecular dynamics trajectory of $DLO1^{apo}$ form, showing a closed conformation of the CTH positioned near the catalytic core.

File name: **Supplementary Movie 4**

Description: Molecular dynamics trajectory of $DLO1^{SA}$ bound state, demonstrating ligand-induced outward movement of the CTH that opens the catalytic pocket.
