# Supplementary material for: Salicylic acid modulates its catabolic enzymes via proteasomal degradation linked to SCF-associated proximity networks
Source: Nat Commun. 2026 Apr 20;17:5468. doi: 10.1038/s41467-026-72241-x (PMC13284210; doi:10.1038/s41467-026-72241-x)
Supplement: Supplementary file 14 — Reporting Summary [file 41467_2026_72241_MOESM14_ESM.pdf]

nature portfolio

Corresponding author(s): Nitzan Shabek
nshabek@ucdavis.edu

Last updated by author(s): Mar 18, 2026

Reporting Summary

Nature Portfolio wishes to improve the reproducibility of the work that we publish. This form provides structure for consistency and transparency in reporting. For further information on Nature Portfolio policies, see our Editorial Policies and the Editorial Policy Checklist.

Statistics

For all statistical analyses, confirm that the following items are present in the figure legend, table legend, main text, or Methods section.

n/a	Confirmed	
☐	☒	The exact sample size (*n*) for each experimental group/condition, given as a discrete number and unit of measurement
☐	☒	A statement on whether measurements were taken from distinct samples or whether the same sample was measured repeatedly
☐	☒	The statistical test(s) used AND whether they are one- or two-sided<br>*Only common tests should be described solely by name; describe more complex techniques in the Methods section.*
☒	☐	A description of all covariates tested
☒	☐	A description of any assumptions or corrections, such as tests of normality and adjustment for multiple comparisons
☐	☒	A full description of the statistical parameters including central tendency (e.g. means) or other basic estimates (e.g. regression coefficient) AND variation (e.g. standard deviation) or associated estimates of uncertainty (e.g. confidence intervals)
☒	☐	For null hypothesis testing, the test statistic (e.g. *F*, *t*, *r*) with confidence intervals, effect sizes, degrees of freedom and *P* value noted<br>*Give P values as exact values whenever suitable.*
☒	☐	For Bayesian analysis, information on the choice of priors and Markov chain Monte Carlo settings
☒	☐	For hierarchical and complex designs, identification of the appropriate level for tests and full reporting of outcomes
☒	☐	Estimates of effect sizes (e.g. Cohen's *d*, Pearson's *r*), indicating how they were calculated

Our web collection on statistics for biologists contains articles on many of the points above.

Software and code

Policy information about availability of computer code

Data collection	No software used.
Data analysis	Western blot data were analyzed in the BioRad Image Lab software suite. Molecular dynamics simulations were carried out using the GROMACS 2020.3 software package. The differential scanning fluorimetry and trypsin-limited proteolysis data were analyzed and plotted in GraphPad. Proximity labeling proteomics data were analyzed and visualized using R and Cytoscape. Structural analysis and imaging were preformed by PyMOL 3.1.3.1. LC/MS-MS metabolite raw data were analyzed using Thermo FreeStyle 1.8 software.

For manuscripts utilizing custom algorithms or software that are central to the research but not yet described in published literature, software must be made available to editors and reviewers. We strongly encourage code deposition in a community repository (e.g. GitHub). See the Nature Portfolio guidelines for submitting code & software for further information.

Data

Policy information about availability of data

All manuscripts must include a data availability statement. This statement should provide the following information, where applicable:
- Accession codes, unique identifiers, or web links for publicly available datasets
- A description of any restrictions on data availability
- For clinical datasets or third party data, please ensure that the statement adheres to our policy

The mass spectrometry proteomics data have been deposited to the ProteomeXchange Consortium via the PRIDE [1] partner repository with the dataset identifiers

PXD063820 and 10.6019/PXD063820 for ASK1-Pst proximity labeling, and dataset identifiers PXD063821 and 10.6019/PXD063821 for DMR6 & DLO1 proximity labeling. Raw data can be accessed with the following accessions and token IDs: Project accession: PXD063821 and Token: 9dzXq2YSmwdq for DMR6 & DLO1 proximity labeling, and Project accession: PXD063820 and Token: nlwC6UEWnq61 for Pst-ASK1 proximity labeling. Raw metabolite quantification data can be found in the source data file associated with this manuscript.

Research involving human participants, their data, or biological material

Policy information about studies with human participants or human data. See also policy information about sex, gender (identity/presentation), and sexual orientation and race, ethnicity and racism.

Reporting on sex and gender	No sex and gender-based analyses performed in this study
Reporting on race, ethnicity, or other socially relevant groupings	N/A for this study
Population characteristics	N/A for this study
Recruitment	N/A for this study
Ethics oversight	N/A for this study

Note that full information on the approval of the study protocol must also be provided in the manuscript.

Field-specific reporting

Please select the one below that is the best fit for your research. If you are not sure, read the appropriate sections before making your selection.

[x] Life sciences   [ ] Behavioural & social sciences   [ ] Ecological, evolutionary & environmental sciences

For a reference copy of the document with all sections, see nature.com/documents/nr-reporting-summary-flat.pdf

Life sciences study design

All studies must disclose on these points even when the disclosure is negative.

Sample size	For experiments with whole adult Arabidopsis or N. benthamiana plants, at least 3-6 plants were used for each experimental condition based on similar studies. For experiments with Arabidopsis seedlings, a minimum of 10-15 seedlings were pooled for each experimental condition similarly based on previous studies.
Data exclusions	No data were excluded
Replication	Each experiment was independently reproduced at least three times.
Randomization	Allocation of plants was not random, but covariates were controlled because all plant material used was grown under identical and controlled conditions in a controlled environment facility. Therefore within each experiment, all plants used were of the exact same age and had been exposed to the exact same conditions.
Blinding	Not blinded.

Reporting for specific materials, systems and methods

We require information from authors about some types of materials, experimental systems and methods used in many studies. Here, indicate whether each material, system or method listed is relevant to your study. If you are not sure if a list item applies to your research, read the appropriate section before selecting a response.

Materials & experimental systems

n/a	Involved in the study	
[ ]	[x]	Antibodies
[x]	[ ]	Eukaryotic cell lines
[x]	[ ]	Palaeontology and archaeology
[x]	[ ]	Animals and other organisms
[x]	[ ]	Clinical data
[x]	[ ]	Dual use research of concern
[ ]	[x]	Plants

Methods

n/a	Involved in the study	
[x]	[ ]	ChIP-seq
[x]	[ ]	Flow cytometry
[x]	[ ]	MRI-based neuroimaging

Antibodies

Antibodies used	Ubiquitin with ubiquitin monoclonal antibody at a 1:1000 dilution (Enzo BML-PW0930), His-tagged proteins with monoclonal 6x-His (Invitrogen MA1-21315) at a 1:2000 dilution, HA-tagged proteins with monoclonal HA-HRP at 1:2000 (Invitrogen #26183-HRP), and myc-tagged proteins with monoclonal myc-HRP at 1:2000 (Invitrogen #R951-25). For non-HRP conjugated monoclonal antibodies, secondary antibody goat anti-mouse-HRP was used at a 1:2000 dilution (Invitrogen #32430). DMR6 antiserum was generated through PhytoAb.
Validation	Antiserum was validated by blotting against recombinant DMR6 and DLO1 as well as native extracts from Col-0 and pUBQ::DMR6-3xHA (Supplementary Fig. 1b,c).

Dual use research of concern

Policy information about dual use research of concern

Hazards

Could the accidental, deliberate or reckless misuse of agents or technologies generated in the work, or the application of information presented in the manuscript, pose a threat to:

No	Yes	
x		Public health
x		National security
x		Crops and/or livestock
x		Ecosystems
x		Any other significant area

Experiments of concern

Does the work involve any of these experiments of concern:

No	Yes	
x		Demonstrate how to render a vaccine ineffective
x		Confer resistance to therapeutically useful antibiotics or antiviral agents
x		Enhance the virulence of a pathogen or render a nonpathogen virulent
x		Increase transmissibility of a pathogen
x		Alter the host range of a pathogen
x		Enable evasion of diagnostic/detection modalities
x		Enable the weaponization of a biological agent or toxin
x		Any other potentially harmful combination of experiments and agents

Plants

Seed stocks	T-DNA insertion mutant (SALK_072882) for Kelch F-box AT4G00893 was obtained from the Arabidopsis Biological Resource Center in Columbus, Ohio (https://abrc.osu.edu/).
Novel plant genotypes	Stable transgenic Arabidopsis lines expressing HA-epitope-tagged DMR6 were produced via the Agrobacterium floral dip method. One independent line was analyzed in experiments, and experiments were performed on the T2 generation.
Authentication	Genotyping PCR and western blot analysis were used to validate the genotypes of the used plants.
