# Supplementary material for: Salicylic acid modulates its catabolic enzymes via proteasomal degradation linked to SCF-associated proximity networks
Source: Nat Commun. 2026 Apr 20;17:5468. doi: 10.1038/s41467-026-72241-x (PMC13284210; doi:10.1038/s41467-026-72241-x)
Supplement: Supplementary file 15 — Transparent Peer Review File [file 41467_2026_72241_MOESM15_ESM.pdf]

nature portfolio

Peer Review File

Salicylic acid modulates its catabolic enzymes via proteasomal degradation linked to SCF-associated proximity networks

Corresponding Author: Professor Nitzan Shabek

Parts of this Peer Review File have been redacted as indicated to remove third-party material.

This file contains all reviewer reports in order by version, followed by all author rebuttals in order by version.

Version 0:

Reviewer comments:

Reviewer #1

(Remarks to the Author)
Comments to the Authors:

I have spent a good amount of time reading and thinking about the manuscript submitted for publication in Nature Communications. The language, style and general presentation are at a high level and fine, no changes needed. The same goes for the technical aspects of the methodology and description thereof. The Introduction conveys the information necessary to understand the results.
My main issue with the paper is that on the one hand it seeks to deliver a mechanistic explanation of DMR6 and DLO1 mode of action in plant immunity by way of regulating SA levels, however the data presented at the moment falls short of this. The later part of the paper becomes broader, reporting a second proximity labeling experiment that no longer directly focusses on DMR6 or DLO1. I suggest this chapter should be omitted and the present data supplemented with further experiments to solidify the conclusions drawn about DMR6 and DLO1 action and their regulation of SA turn-over and its relevance in plant immunity. More specifically, see my comments to the individual sections below. The discussion should be modified accordingly.

Comments to section 1 of the Results:
I find some of the presented in planta and cell free degradation analysis data inconclusive.
1. In the Arabidopsis DMR6 overexpressing lines, 3 hours of exogenous SA treatment led to DMR6 degradation which was reduced when MG132 was applied (Figure 1c). Was MG132 applied for the same duration as SA and why did the proteasome inhibitor not fully restore DMR6 levels after 3 hours of SA treatment.
2. In the wild type the authors report DMR6 degradation following 1 hour of SA treatment which was abolished by MG132 treatment, however the signal intensity of the bands in western visually looks more or less the same (Figure 1d). Also the MG132 band for SA does not really suggest degradation arrest.
3. The CHX chase experiments depicted in figure 1e and 1f show increased degradation rate of DMR6 and decreased degradation rate/stabilization of DLO1 dependent on SA. How many times were the experiments as well as the ones mentioned above (figures 1 c and d) performed? I couldn’t find this information in the main text, materials and methods or figure legends.
4. The band intensities in figures 1g (more rapid DMR6 degradation dependent on SA in cell-free degradation assay) and 1k (catalytically inactive mutant degrades slower than wild type) are not well suited for quantifying the relatively small changes that are reported because they look essentially the same.
The authors would be well advised to additionally quantify DMR6 and DLO1 in these assays with another technique such as targeted mass spectrometry (SRM, PRM) to substantiate their conclusions. Also, SA dependent ubiquitylation of DMR6 and DLO1 should be shown, preferably by targeted MS of di-Gly modified peptides, but an anti-body based strategy should also be possible. Additionally, as DMR6 and DLO1 are SA hydroxylases and presumably affect SA levels and immunity by clearing the active hormone, SA hydroxylation products 2,5-DHBA and/or 2,3-DHBA should be quantified, again via targeted MS (SRM).

Comments to section 2 of the Results:
I looked at the AphaFold models of DMR6 and DLO1 and the pLDDT was above 90 for most of the protein structure for both proteins, so the models should be valid to be used as the beginning of further modeling studies. I cannot comment on anything beyond this, because computational protein modeling is not my area of expertise.
It struck my attention that the bar charts in figures 2g and 2h seem to be swapped, i.e. the DMR6 bar chart seems to fit the DLO1 gel and vice versa, please check or explain. Figures 2j and 2k plot average melting curves as stated in figure legends, please add error bars or some measure of dispersion (error band indicating confidence interval around the mean, for instance). In figure 2n, how many experiments were performed?

Comments to section 3 of the Results
Section 3 describes Bio-ID (Turbo-ID) based proximity labeling of potential DMR1 and DLO6 interaction partners. The authors are experienced with Bio-ID experiments having previously published ASK1 interactomes using this technique. I am curious to know why Citrine-TurboID was used as a control as oppose to wild type. Looking at the Western Blots in Figure S5a it seems Citrine interacts unspecificaly with most cytoplasmic proteins producing a background biotinylation signal that I would interpret as a type of positive control. If this is the case I see why a fold-change in abundance of proteins in DMR6 or DLO1 preparations would indicate specific interaction. Nevertheless, why not use a negative control as wild type. In our hands this produced almost no background signal in contrast to the eLIFE paper by Mair et al. Is the q-value threshold perhaps to high, what about alpha = 0.05?
I do think the authors should verify the interaction of potential partners that they touch upon in the manuscript with another technique such as BiFC as they have done before. At the very least this should be done for the F-Box candidate AT4G00893, which they study in more detail.

Comments to section 4 of the Results.
This section seems somewhat detached from the story and comes across like an add-on although a substantial one. I would remove it because it doesn't really add anything to the mechanistic insights of DMR6 and DLO1 mode of action in SA dependent plant defense, but instead broadens the narrative in a more or less purely descriptive manner. It may form the basis of another paper.
1. At this point, after having verified the interaction of AT4G00893 with DMR6 and DLO1 as requested above, DMR6 and DLO1 ubiquitylation, abundance should be monitored in the AT4G00893 mutant background after SA treatment and PstDC300 infection with targeted mass spectrometry approaches as explained under comments to section 1.
2. PstDC3000 infection assays (bacterial recovery) in the DMR6, DLO1 and AT4G00893 mutant backgrounds should be included to show that these proteins directly affect plant defense. These experiments were described in the materials and methods section, but I couldn't find anything relating to them in the results.
3. How do the authors reconcile exogenous SA dependent degradation of DMR6 with PstDC3000 dependent stabilization of DMR6 when PstDc3000 infection is known to lead to a rapid and substantial increase in SA levels? Measurements of SA and hydroxylation products in these infection scenarios should be performed to shed light on this.

Comments to figure 5 (model).
The model should be expanded according to incorporate results of the additional experiments. For instance, ubiquitylation should be added before degradation when it has been measured. Also, AT4G00893 and the results of the patho-assays could be added.

Reviewer #2

(Remarks to the Author)
This manuscript investigated the regulation of the SA hydroxylases DMR6 and DLO1 by proteosome-mediated degradation. The authors found that SA binding triggers conformational changes in DMR6, particularly in the conserved C-terminal helix. These changes make DMR6 susceptible to degradation. The DLO1 C-terminal helix is less conserved, and SA binding-induced conformational change is not significantly, As the result, SA induces DMR6 degradation but stabilizes DLO1. Using Turbo-ID proximity labeling, the authors found a putative F-box protein that appears to be involved in DMR6 and DLO1 degradation and that DC3000 infection induces the ASK1-SCF proxeome reprogramming.
Major comments:
The authors tested DMR6 and DLO1 degradation under multiple conditions. One is cell-free or in vitro degradation assay. Figure 1g,h and Supplementary Figure 2a,b are cell-free or in vitro degradation. His-DMR6 degradation is much faster in the Supplementary Figure. It seems the authors used EtOH to dissolve SA and water to dissolve 2,5-DHBA. Is the reduced degradation in Figure 2a,b caused by EtOH? The authors may need to use sodium salicylate in water to accurately determine the half-life of the protein.
Since SA is a substrate of DMR6, it is not surprising that SA binding causes conformational changes in DMR6, affects trypsin-limited proteolysis, and makes DMR6 more susceptible to proteosome-mediated degradation. Although defining the exact conformational change at the CTH is significant, it does not explain the underlying mechanism.
Using TurboID proximity labeling, the authors identified a Kelch-domain F-box protein and provided evidence that the Arabidopsis ortholog is involved in DMR6 degradation. F-box protein is known to bind to substrate and the authors also predicted an interface between the Kelch domain and DMR6 (Supplementary Figure 7b). SA binding may enhance the interaction between the Kelch domain and DMR6, which would explain the observed result. This needs to be tested, since the authors have all the materials in hand.
The regulation of in vivo DMR6 degradation is more difficult to dissect, since pathogen infection increase DMR6 protein levels. This is likely due to increased gene expression, as the authors proposed. On the other hand, CHX and MG132 treatments (Figure 1k,l, it is not clear if SA was added here) indicate that DMR6 is intrinsically unstable in the absence of SA

treatment (if SA was not added). The D214A mutant is stable, indicating this mutation could break the DMR6-F-box protein interaction. This should be tested as well.
NPR1 is known to suppress SA accumulation by repressing ICS1 expression. It would be interesting to test if SA signaling instead of SA per se is the enhancer of DMR6 degradation. This could be tested using the DMR6 antibody and npr1 mutants.
Minor comments:
Supplementary Figure 1c, at first glance, it was surprising to see the size difference between 6xHis-DMR6 and untagged DMR6. The figure legend says it is actually 6xHis-MysB-DMR6.
Figure 1k,l, the basal level of DMR6-3xHA in (l) is extremely low compared to that in (k).
Supplementary Figure 5a, Myc signal is missing in some samples?
Figure 3h, what happened in wild type?
Supplementary Figure 7b, is the CTH required for DMR6-Kelch interaction if it is a degron-like module recognized by UPS as the authors proposed?

Reviewer #3

(Remarks to the Author)
Shabek and co-workers studied dual-scale regulatory mechanism for maintaining salicycilic acid homeostasis. The work is mainly focused on biochemical experiments and uses molecular dynamics simulation for supporting the experimental findings. I was specifically asked to review the molecular dynamics simulations reported in this manuscript. I have major concerns about the MD part of this manuscript, which are included below. The manuscript could have benefited from a collaboration with a MD expert who could have easily addressed several critical simulation setup issues with the current work. I believe that this is an excellent manuscript which could be published after major revisions to the molecular modeling section.

Comments
1. Choice of Force Field: The authors have employed the CHARMM36 force field for their simulations. However, CHARMM36m is an improved and widely adopted version that provides better backbone sampling, particularly for intrinsically disordered regions and proteins. Can the authors justify their choice of CHARMM36 over CHARMM36m? Given the improvements and widespread use of CHARMM36m in recent literature, I strongly recommend that the authors consider repeating key simulations using CHARMM36m and compare the results with their current findings.

2. Choice of Barostat: Authors have used Berendsen barostat for their simulations. It is a widely used barostat available in the GROMACS package. Several studies have shown that the Berendsen barostat gives incorrect ensembles. Therefore, I would advise authors to change the barostat in their simulations. Please refer to (1) J. Chem. Theory Comput., 2013, 9, 2, 909–926; (2) J. Chem. Theory Comput. 2015, 11, 12, 5583–5597 for more information on the issues related to Berendsen barostat.

3. Simulation Replicates: For robust statistical interpretation and to account for stochastic variability in MD, it is standard practice to run at least three independent replicates for each condition. If replicates were not performed, I recommend conducting them and using the results to estimate statistical errors for all major analyses. This will help ensure that the observed trends are reproducible and not artefacts of a single trajectory.

4. Simulation Length: The simulations are limited to 100 ns, which may not be sufficient to capture slow or large-scale conformational changes. Recent studies commonly use simulation timescales in the microsecond regime, often discarding the initial few hundred nanoseconds as equilibration. Can the authors justify why 100 ns was considered sufficient in this context? If computationally feasible, extending the simulations would strengthen the conclusions drawn from the data.

5. Ligand Stability and Pose: The initial ligand pose appears to be selected based on docking scores. However, Fig. 2c and Fig. 2f suggest that the ligand may not remain stably bound in that configuration. I recommend that the authors include ligand-specific RMSD plots over time to track deviations from the initial docking pose. Additionally, performing longer simulations or starting from alternative binding poses could provide more insight into ligand binding stability and dynamics. This would also address concerns regarding the validity of the initial configuration used in the simulations of the SA-bound state.

6. Conformational Changes in DLO1 and DMR6: The authors refer to conformational changes in DLO1 and DMR6, but only provide RMSD as supporting data. RMSD primarily reflects deviation from the starting structure and does not directly quantify biologically relevant conformational changes. I suggest incorporating more targeted metrics such as inter-residue distances, domain angles, or catalytic pocket volume. Such analyses would allow clearer identification and comparison of open versus closed conformations and help better contextualize the observed dynamics between DLO1 and DMR6.

7. Analysis of the predicted alphafold structures. A detailed analysis of the AF2 predicted structures should be included in SI. Furthermore, it is well-known that these models are not able to accurately predict the side chain orientations. Therefore, the stability analysis for the Fe^{3+} ion placed in its binding site should be performed. This ion has high positive charge and I suspect that the structural changes in the protein could simply be caused by using even a slightly incorrect coordination of the ion binding residues.

8. The goal of the MD simulations should be to provide atomistic insights into the mechanism of structural instability. The conclusions reported in the manuscript about structural dynamics are highly suspect given the numerous issues with the simulation setup. However, even if we take the results at their face value, the authors have merely used simulations to generate an observation but failed to provide the biophysical reason for the observation. A more detailed analysis of the simulations should be performed.

Reviewer #4

(Remarks to the Author)
I co-reviewed this manuscript with one of the reviewers who provided the listed reports. This is part of the Nature Communications initiative to facilitate training in peer review and to provide appropriate recognition for Early Career Researchers who co-review manuscripts.

Version 1:

Reviewer comments:

Reviewer #1

(Remarks to the Author)
The authors have gone to some lengths to address the points I raised in my original review. Nevertheless, I am still somewhat less than enthusiastic about some of the quantitative data presented. The authors have gone to great length to explain to me why SA dependent changes in endogenous DMR6 levels are modest and difficult to quantify looking at the western blot alone (new figure 1d). However, this is actually a quintessential piece of information as it represents the physiological in plant situation which seems to be quite subtle.
The authors added a quantitative ratiometric fluorescence assay that shows that DMR6 is degraded more rapidly dependent on SA whereas there is no effect on DMO1. The original cell free degradation assays have been moved to the supplements, more rapid degradation of DMR6 dependent on SA remains inconclusive to me from this figure, indeed I would doubt that the changes at 90 min or even at 180 min are significant. The stabilization of DMO1 dependent on SA is more convincing (new Supplementary figure 2). The authors have however offered some explanations.
Figure 1h presents data that catalytically inactive mutant degrades slower than wild type, but again changes are not really visible to the eye and seem extremely modest. Are they even significant? This is reconciled somewhat in the cell free degradation assays depicted in figure 1j and k.
The authors later characterize the F-Box protein identified in their Turbo-ID experiment as interacting with DMR6 and call it DAF1. They verify interaction with DMR6 by way of co-immunoprecipitation and show stabilization of DMR6 in the daf1 mutant, suggesting that DAF1 promotes proteasomal turn-over, which is supported by the ratiometric assay.
The authors continue to make a case for DMR6 accumulation in the daf1 mutant following SA exposure however Figure 4i, which is supposed to show this, is not clear to me (everything seems to be in the daf1 background, where is the comparison to Col-0?) and the quantitative differences are less than modest, although this is explained. Supplemental figure 9c shows strongly reduced DMR6 abundance in the daf1 background and the conclusion that this is due to “a self-limiting SA-DMR6 feedback loop that fine-tunes DMR6 abundance” is not sufficiently explained and may also be overinterpreted. Reduced DMR6 ubiquitylation is shown by way of a DMR6 pull-down and anti-Ub western, but again differences are hardly visible so the quantification is not conclusive suggesting that the phenomenon of DAF1 mediated DMR6 ubiquitylation is hardly relevant.
The authors have quantified both SA and 2,5-DHBA levels and 2,5-DHBA is reduced in the daf1 mutant. This is counterintuitive at first, as an absence of DAF1 implies attenuated proteasomal degradation and increased DMR6 abundance, the authors reconcile this with a self-limiting regulatory feedback loop where DMR6 abundance is controlled both by SA induced transcriptional induction as well as DAF1 mediated, SA dependent proteolysis.
The authors state that the daf1 mutant shows increased resistance to Pst DC3000 infection (less necrotic lesions) suggesting the protein is a negative regulator of immunity. At the same time DMR6 is also cited as a negative regulator, making it complex to reconcile the functional interaction between these two proteins in light of the papers primary narrative, that DAF1 leads to DMR6 ubiquitylation and proteasomal degradation.
In summary I think a plethora of experiments have been conducted yet the quantified phenomena are often very modest and some of the data remains conflicting or at least difficult to reconcile in a clear mechanistic model.

Reviewer #2

(Remarks to the Author)
In this revised manuscript, the authors have addressed my previous comments and questions and have updated their model by incorporating a self-limiting SA-DMR6-DAF1 feedback loop. Within this loop, SA promotes DMR6 transcription while simultaneously enhancing DAF1-mediated proteasomal degradation of DMR6, thereby modulating cellular DMR6 abundance and consequently SA homeostasis. This model positions DAF1 as a central regulatory component that likely contributes positively to plant immunity. Consistent with this notion, the authors report that DAF1 overexpression induces SA-dependent cell death, whereas pathogen-induced cell death is attenuated in the daf1 T-DNA insertion mutant.

However, the disease susceptibility analysis presented for daf1 is not convincing. The bacterial growth data in Figure 4j appear incorrect. Following spray inoculation, Pst DC3000 typically proliferates by approximately 1000-fold in Col-0 plants

after three days (Melotto et al., 2006, Cell 126:969-980), yet essentially no bacterial growth was observed in the presented results. To ensure reliable interpretation, the authors should perform leaf infiltration to bypass potential confounding effects of stomatal immunity. It should be noted that the virulence of Pst DC3000 can decline following repeated subculture or prolonged storage.

Furthermore, to substantiate the proposed self-limiting feedback regulation, the authors are encouraged to compare SA- and pathogen-induced DMR6 transcriptional kinetics in Col-0 and daf1, along with dynamic measurements of pathogen-induced SA accumulation in both genotypes. These experiments are technically feasible for the authors and would be expected to provide strong mechanistic evidence supporting the revised model.

Reviewer #3

(Remarks to the Author)
Authors have fully addressed the comments from the first round of review. I congratulate the authors on their excellent work.

Reviewer #4

(Remarks to the Author)
I co-reviewed this manuscript with one of the reviewers who provided the listed reports. This is part of the Nature Communications initiative to facilitate training in peer review and to provide appropriate recognition for Early Career Researchers who co-review manuscripts.

Version 2:

Reviewer comments:

Reviewer #1

(Remarks to the Author)
I thank the authors for the time they have taken in addressing my previous comments. I am beginning to appreciate their line of argumentation that the plethora of experiments show a potential feedback loop regulating DMR6 levels, composed on the one hand of SA induced synthesis and on the other by DAF1 mediated DMR6 ubiquitylation and subsequent proteasomal degradation and that this accounts for the nuanced effects observed in many experiments. Also, interesting data on pathogen susceptibility and immunity dependent on DAF1 are shown, characterizing the protein as well as the structural models that support SA dependent conformational shift and changes in stability. The story is the placed into the larger context of protein interaction networks in an infection scenario although I would have liked to see this in the daf1 background to connect the last section with the sections before it.
I think at this point no further experiments will be necessary as many have been performed already producing interesting data that, although in some cases is seemingly contradictory, does support the hypothesis of the feedback loop. However, I think it goes too far to state that it has been unequivocally shown. Therefore, I think the authors should edit the manuscript one more time focusing on making things clearer and phrasing some of their conclusions more carefully. Below are a summary of and a few points that arose in the section reporting the daf1 experiments, which is the last one I have any major issues with.

Lines 436 to 440: DAF1 leads to DMR6 and DLO1 degradation. Fig4 g and h show less protein in Wt compared to daf1.

Lines 440 to 444: In planta situation. DMR6 is very low abundant in daf1 compared to Wt background although it accumulates to slightly higher levels in the mutant than the wild type when proteasome inhibitor is applied. Shouldn't the DAF1 knockout be sufficient to attenuate degradation and lead to DMR6 accumulation? This seems to be the conclusion that can be drawn from the cell free degradation assays above. It hardly seems to be the case in planta as levels are more or less the same when comparing 0, 1 and 2 hours of SA treatment alone without proteasome inhibitor. Addition of MG132 enhances the effect and it seems to me the authors are stating that due to the additional application of SA, DMR6 synthesis induced by SA and degradation mediated by DAF1 jointly modulate DMR6 levels, the later introduced regulatory feedback loop.

Lines 444 to 450: Why is DMR6 so low abundant in the daf1 background? Looking at Suppl fig 9c I can see that DMR6 is degraded 1 hour after DMSO treatment to 0.75 without the application of SA. Also note, comparing to Figure 1d, degradation of endogenous DMR6 is similar with application of SA (0.68). In line 450 the authors state the differences in DMR6 abundance between genotypes supports the feedback loop model but this seems very speculative at this point.

Lines 451 to 457: DMR6 ubiquitylation is reduced in daf1 background showing aberrant DMR6 protein abundance regulation in the genotype. Does DAF1 mediate DMR6 ubiquitylation and so it accumulates in the knockout because less is ubiquitylated and hence targeted for degradation? What happens endogenously when you treat the daf1 only with MG132 but not SA analogous to figure 4i?

Lines 458 to 463: No DMR6 in npr1. Has it been shown that NPR1 is a transcriptional activator of DMR6?
Lines 467 to 469: DAF1 promotes DMR6 degradation and DMR 6 hydroxylates SA to produce 2,5-DHBA. Why are 2,5-

DHBA levels lower when there is no DAF1 as in daf1? The authors state DMR6 activity is reduced in daf1. Where have they shown this? I see no activity assays, enzyme kinetics or anything of the sort in the daf1 background, if I haven't missed it. I see the explanation in lines 660 to 666 of the discussion but it is speculative.

Lines 473 to 478: I can appreciate the authors hypothesis of the concurrent SA induced transcriptional regulation concurrent with the SA induced, DAF1 mediated DMR6 turn-over controlling SA levels to a point, in other words I see hints at this in the presented data, but I believe it is still too speculative. The whole section and conclusions need to be formatted much cautiously also in the discussion.

Lines 487 to 495: I think this whole section should be deleted, it adds no relevant information and interrupts the story which continues on line 496. Loss of DAF1 in daf1 leads to increased immunity. The authors state in lines 484 to 486 that DMR6 is a negative regulator of immunity and that DAF1 promotes its turnover (do you mean degradation Fig 4g), so wouldn't loss of DAF1 promote accumulation of the negative regulator thereby suppressing immunity? Please explain this. In line, DAF1 overexpression is reported to lead to a type of auto-immune phenotype, i.e. leaf necrosis which is alleviated by DMR6 co-expression or depletion of SA by NahG co-expression, suggesting dependence of auto-immunity on SA and regulation by both DMR6 and DAF1. These are interesting findings but should be clarified.

Section on ASK1 Turbo-ID after Pst DC3000 inoculation: The experiment itself is interesting but as I already remarked in an earlier review, DMR6 and DAF1 are dropped from the narrative at this point. What about the same TurboID experiment in daf1 background as it seems it has an effect on plant immunity? Maybe for future studies.

Discussion

Generally, no issues, but I think it goes too far to say the feedback loop has been clearly shown. Discussion should be phrased more carefully.

Reviewer #2

(Remarks to the Author)
It is surprising that the authors did not attempt to properly verify the defense phenotype of the daf1 mutant, given that this is a critical piece of evidence required to support their biochemical conclusions. Without accurate phenotypic characterization, the interpretation of the biochemical data remains uncertain. The daf1 mutation could plausibly alter pathogen-induced SA accumulation and thereby increase or decrease resistance to bacterial pathogens. These possibilities would fundamentally influence how the biochemical results should be understood.

Simply relocating the flawed resistance-assay data to the supplementary information does not resolve the issue, as the underlying experiment was not performed correctly based the pathogen growth on the wild-type plants and the results as presented are misleading. While the biochemical analyses are interesting, rigorous mutant phenotyping is essential in this case. I would request that the editor extend the revision deadline so the authors can properly determine the true defense phenotype of the daf1 mutant.

Open Access This Peer Review File is licensed under a Creative Commons Attribution 4.0 International License, which permits use, sharing, adaptation, distribution and reproduction in any medium or format, as long as you give appropriate credit to the original author(s) and the source, provide a link to the Creative Commons license, and indicate if changes were made.
In cases where reviewers are anonymous, credit should be given to 'Anonymous Referee' and the source.
The images or other third party material in this Peer Review File are included in the article's Creative Commons license, unless indicated otherwise in a credit line to the material. If material is not included in the article's Creative Commons license and your intended use is not permitted by statutory regulation or exceeds the permitted use, you will need to obtain permission directly from the copyright holder.
To view a copy of this license, visit https://creativecommons.org/licenses/by/4.0/

Point-by-point response to reviewers

Reviewer #1

Comments to the Authors:

I have spent a good amount of time reading and thinking about the manuscript submitted for publication in Nature Communications. The language, style and general presentation are at a high level and fine, no changes needed. The same goes for the technical aspects of the methodology and description thereof. The Introduction conveys the information necessary to understand the results.
My main issue with the paper is that on the one hand it seeks to deliver a mechanistic explanation of DMR6 and DLO1 mode of action in plant immunity by way of regulating SA levels, however the data presented at the moment falls short of this. The later part of the paper becomes broader, reporting a second proximity labeling experiment that no longer directly focusses on DMR6 or DLO1. I suggest this chapter should be omitted and the present data supplemented with further experiments to solidify the conclusions drawn about DMR6 and DLO1 action and their regulation of SA turn-over and its relevance in plant immunity. More specifically, see my comments to the individual sections below. The discussion should be modified accordingly.
[Response] We thank the reviewer for their thoughtful evaluation and constructive summary. In the revised version, we substantially strengthened the experimental basis and mechanistic interpretation of DMR6 and DLO1 regulation in relation to SA turnover and plant immunity. The new data include (i) quantitative ratiometric degradation assays confirming SA-dependent proteasomal turnover of DMR6 and differential stability of DLO1, (ii) in vitro and in planta validation of the DMR6–DAF1 (F-box) interaction and its impact on enzyme degradation and immunity, (iii) LC–MS/MS quantification of SA and 2,5-DHBA levels consistent with altered SA metabolism in the *daf1* mutant, and (iv) extensive refined structural and biochemical analyses. We also reorganized and modify the Results and Discussion to maintain a coherent focus on DMR6 and DLO1 while integrating the ASK1 proximity-labeling data as contextual evidence of SCF network remodeling. We hope the reviewer will find in the following detailed point-by-point responses that we carefully addressed all comments, which we found both important and insightful, resulting in a substantially improved and cohesive manuscript that reinforces the proposed regulatory model.

Comments to section 1 of the Results: I find some of the presented in planta and cell free degradation analysis data inconclusive.
1. In the Arabidopsis DMR6 overexpressing lines, 3 hours of exogenous SA treatment led to DMR6 degradation which was reduced when MG132 was applied (Figure 1c). Was MG132 applied for the same duration as SA and why did the proteasome inhibitor not fully restore DMR6 levels after 3 hours of SA treatment.
[Response] We thank the reviewer for pointing out the potentially confusing annotation. Indeed, SA and MG132 were co-applied throughout the full duration of the experiment shown in Fig. 1c. <u>This is now explicitly stated in the Figure, Figure Legend, Results, and Methods sections to ensure clarity.</u>

Regarding the observation that MG132 treatment did not fully restore DMR6 levels, we note that MG132 is a well-established peptide inhibitor of the 26S proteasome, acting primarily on the 20S catalytic (Rui et al 2012). However, MG132 is also known to incompletely block degradation of highly labile substrates in both plant and animal systems. Partial inhibition is commonly attributed to several factors, including: (i) the existence of secondary (specific or not) proteolytic pathways, (ii) variability in MG132 peptide stability or cellular uptake, and (iii) differential tissue permeability or metabolic inactivation of the inhibitor. We have revised the Results to read:

"*In this and subsequent degradation assays, MG132 consistently stabilized DMR6 but did not completely prevent its degradation. This pattern likely reflects a combination of factors, including secondary proteolytic routes, variable MG132 peptide stability, and differential uptake of the inhibitor by plant tissues*[12-17]."

We have also cited six representative studies reporting that MG132 effectively but incompletely blocks degradation of unstable targets, consistent with our observations (e.g., PMID #: 30647455, 11713520, 35484202, 22902562, 26004230).

We also note that DMR6 transcript abundance is itself induced by SA, potentially counteracting complete stabilization at the protein level. Thus, simultaneous induction of DMR6 transcription and proteasome inhibition may yield partial recovery of steady-state protein levels rather than full restoration. These clarifications strengthen the interpretation that MG132 substantially inhibits, but does not fully abolish, SA-induced DMR6 degradation, which is consistent with its behavior as a highly dynamic and proteasome-regulated enzyme.

2. In the wild type the authors report DMR6 degradation following 1 hour of SA treatment which was abolished by MG132 treatment, however the signal intensity of the bands in western visually looks more or less the same (Figure 1d). Also the MG132 band for SA does not really suggest degradation arrest.

[Response] We thank the reviewer for raising this point. We agree that differences in endogenous DMR6 band intensity in Fig. 1d are modest when viewed directly, and we have clarified this point in both the Results and figure legend. These data represent endogenous DMR6 expressed from its native promoter rather than overexpressed protein, which naturally yields weaker but physiologically relevant signals. To improve clarity, we revised figure legend and the **Results**:

"To determine whether endogenous DMR6 in wild-type Col-0 is also regulated during SA treatment, we generated a specific anti-DMR6 antibody to monitor DMR6 expressed under its native promoter (see Methods; Supplementary Fig. 1b–c). DMR6 protein abundance modestly decreased one hour after SA treatment, and this reduction was substantially stabilized by MG132, although degradation was not completely prevented (Fig. 1d). The effect on endogenous levels was expectedly less pronounced than in overexpression lines, consistent with simultaneous SA-induced transcriptional upregulation of DMR6 [18], which can partially offset proteasome-dependent turnover."

To our knowledge, this is the first report directly monitoring DMR6 protein levels following SA treatment and proteasome inhibition in planta. Because SA simultaneously induces *DMR6* transcription, CHX (cycloheximide) was not applied to avoid interference with MG132 activity; this experimental design allows limited new protein synthesis that can counteract complete stabilization. As reference, we include Fig. R1 (adapted from Zhang et al., 2017, Plant Physiology), which shows that DMR6 transcript levels increase more than threefold within one hour of SA treatment, consistent with our interpretation that transcriptional upregulation accompanies proteasome-dependent turnover. The antibody used in these

[editorial note: third party material redacted]

Fig. R1 for reviewer (Zhang et al, 2017)

experiments was custom-generated (PhytoAB) and validated against recombinant DMR6, confirming specificity. The *in planta* degradation assays were repeated independently with consistent trends, supporting the reproducibility of the MG132 stabilization effect.
Together, these clarifications ensure that the data are interpreted correctly, as subtle but reproducible stabilization of endogenous DMR6 by MG132, consistent with a dynamic balance between SA-induced synthesis and proteasome-mediated turnover.

3. The CHX chase experiments depicted in figure 1e and 1f show increased degradation rate of DMR6 and decreased degradation rate/stabilization of DLO1 dependent on SA. How many times were the experiments as well as the ones mentioned above (figures 1 c and d) performed? I couldn't find this information in the main text, materials and methods or figure legends

[Response] We thank the reviewer for pointing out this omission. All degradation experiments, including those in Figs. 1c–f were independently repeated three to five times with consistent results. This information has now been explicitly added to the Methods, Results, and Figure 1 legend. We also clarified in the figure legend that the data shown are representative of three independent biological replicates and that band intensities were quantified using Bio-Rad Image Lab.

4. The band intensities in figures 1g (more rapid DMR6 degradation dependent on SA in cell-free degradation assay) and 1k (catalytically inactive mutant degrades slower than wild type) are not well suited for quantifying the relatively small changes that are reported because they look essentially the same.

The authors would be well advised to additionally quantify DMR6 and DLO1 in these assays with another technique such as targeted mass spectrometry (SRM, PRM) to substantiate their conclusions. Also, SA dependent ubiquitylation of DMR6 and DLO1 should be shown, preferably by targeted MS of di-Gly modified peptides, but an anti-body based strategy should also be possible.

Additionally, as DMR6 and DLO1 are SA hydroxylases and presumably affect SA levels and immunity by clearing the active hormone, SA hydroxylation products 2,5-DHBA and/or 2,3-DHBA should be quantified, again via targeted MS (SRM).

[Response] We thank the reviewer for these constructive suggestions and have substantially strengthened this part of the study. The original cell-free degradation assay has now been complemented by a quantitative ratiometric fluorescence assay, which provides higher-precision measurement and dynamic resolution of DMR6 and DLO1 protein stability. In this system, DMR6 and DLO1 were fused to Venus–mScarlet reporters and transiently expressed in *N. benthamiana*. Following SA or EtOH treatment, fluorescence ratios were monitored over time. The results (now new Fig. 1g) show that SA treatment significantly accelerates DMR6 degradation but has little effect on DLO1, confirming the in planta trend. The previous cell-free degradation data are retained as Supplementary Fig. 2a–b, with additional brief discussion of their limitations under the Results:

"... we also performed a cell-free degradation assay using recombinant DMR6 and DLO1 proteins incubated with wild-type Arabidopsis extracts (Supplementary Fig. 2a–b). This assay recapitulated proteasome-dependent turnover for both enzymes and revealed that DLO1 displayed pronounced stabilization in the presence of SA and MG132, whereas DMR6 remained rapidly degraded. The magnitude of SA responsiveness was lower for DMR6 than in planta, likely reflecting the absence of subcellular compartmentalization and residual endogenous SA in the extract. "

We took this opportunity to clarify several excellent points raised by the reviewer and integrated them throughout the revised Results and Introduction sections:

1. Cell-free assay and SA sensitivity. While the assay strongly supports proteasome-dependent degradation of DMR6 and DLO1, exogenous SA addition was not productive, likely due to residual endogenous SA in concentrated extracts (thus resulting in the addition of exogenous SA having less of a differential effect) and the loss of subcellular compartmentalization. Moreover, recombinant DMR6 is extremely sensitive to small amounts of SA (K_m = 5.15 ± 1.44 μM; Zhang et al., 2017), which may explain the diminished SA response in vitro. By moving these data to the Supplementary section, we both address this point and preserve additional biochemical evidence for proteasome-dependent degradation while transparently noting assay limitations.
2. Ubiquitination evidence. We expanded the text to clearly connect DMR6 and DLO1 ubiquitination to their proteasomal degradation. Ubiquitination of both enzymes has been independently demonstrated: (a) our previous ASK1 (SCF) proximity-labeling study showed co-immunoprecipitation of HA–Ub with myc-tagged DMR6 and DLO1 (Sun et al., New Phytologist, 2024; DOI 10.1111/nph.20014); and (b) a ubiquitylome study identified di-Gly-modified DMR6 and DLO1 peptides (Walley et al., Plant Cell, 2024; DOI 10.1093/plcell/koae310).

   This part is now better clarified under the Introduction:

   "Our recent proximity-labeling analysis of ASK1 (Arabidopsis SKP1 homolog) and CULLIN1, both tagged with TurboID, identified DMR6 and DLO1 among candidate SCF complex substrates, suggesting a potential link between SA catabolism and SCF-mediated protein degradation9. Overexpression of tagged DMR6 and DLO1 in tobacco leaves can co-immunoprecipitate tagged ubiquitin, showing a characteristic polyubiquitination pattern. Moreover, a recent proteomic study further detected di-glycine (ubiquitin remnant) modifications on DMR6 and DLO1, providing orthogonal evidence that these enzymes are bona fide ubiquitination targets.[9,10]"

   In addition to this point, we have also included new experimental data (Supplementary Fig. 9d) showing that polyubiquitin levels of endogenous DMR6 are slightly elevated in wild-type plants compared with the E3 mutant (termed DAF1 in the revised version). Although this difference is not statistically significant, as might be expected when monitoring transient in planta ubiquitination, altogether with the observed trend is fully consistent with the model that DMR6 is a ubiquitinated substrate targeted by the SCF-type E3 ligase.
3. SA and 2,5-DHBA quantification. We conducted new LC–MS/MS analyses of SA and its catabolite 2,5-DHBA (new Supplementary Fig. 9f–g). Basal SA was detectable even without exogenous treatment, and 2,5-DHBA levels were significantly reduced in the *daf1* mutant, consistent with altered DMR6 turnover and SA homeostasis. As discussed in few points throughout the manuscript, these values are in line with Zhang et al. (2017), who precisely measured SA and 2,5-DHBA in DMR6/DLO1 overexpression and mutant lines (see Figure R2, for reference).

[editorial note: third party material redacted]

Fig. R2 for reviewer (Zhang et al, 2017)

We appreciate the reviewer's suggestion to employ targeted MS (SRM/PRM) for direct quantification of DMR6 and DLO1 protein levels. However, the extremely low endogenous abundance of these enzymes, particularly when expressed from their native promoters, renders targeted MS technically infeasible without isotope-labeled internal standards.
The newly implemented ratiometric fluorescence system provides an equally quantitative and reproducible readout with real-time temporal resolution that is difficult to achieve by SRM/PRM, and thus effectively fulfills the reviewer's objective for robust and precise measurement of DMR6 and DLO1 stability.
Together, these revisions strengthen this section by adding a higher-precision quantitative assay, clarifying the scope of the cell-free experiment, consolidating evidence of ubiquitination, and including direct metabolite measurements that further support our conclusions.

Comments to section 2 of the Results:

It struck my attention that the bar charts in figures 2g and 2h seem to be swapped, i.e. the DMR6 bar chart seems to fit the DLO1 gel and vice versa, please check or explain. Figures 2j and 2k plot average melting curves as stated in figure legends, please add error bars or some measure of dispersion (error band indicating confidence interval around the mean, for instance). In figure 2n, how many experiments were performed?
[Response] We thank the reviewer for their close attention to this oversight. The bar charts in the original Figures 2g and 2h were indeed swapped (now Figures 3a and b in the revision), and this has now been corrected. We also replotted the DSF melting curves with the standard error across triplicates shown as shaded error envelopes, which are now included in the revised Figure 2 (now Figures 3d and e in the revision). As with all degradation assays, the analysis shown in Figure 2 (now Figure 3h) was performed three independent times, now clarified in both the figure legend and the Methods section.
Finally, we bring to the reviewer's attention that the figure numbering has shifted in the revised manuscript due to the addition of new structural analyses that improved the overall flow of the paper; as a result, the previous Figure 2 is now Figure 3.

Comments to section 3 of the Results
Section 3 describes Bio-ID (Turbo-ID) based proximity labeling of potential DMR1 and DLO6 interaction partners. The authors are experienced with Bio-ID experiments having previously published ASK1 interactomes using this technique. I am curious to know why Citrine-TurboID was used as a control as oppose to wild type. Looking at the Western Blots in Figure S5a it seems Citrine interacts unspecificaly with most cytoplasmic proteins producing a background biotinylation signal that I would interpret as a type of positive control. If this is the case I see why a fold-change in abundance of proteins in DMR6 or DLO1 preparations would indicate specific interaction. Nevertheless, why not use a negative control as wild type. In our hands this produced almost no background signal in contrast to the eLIFE paper by Mair et al. Is the q-value threshold perhaps to high, what about alpha = 0.05?
[Response] The reviewer brings up an excellent and valid point; the appropriate control for proximity labeling experiments is something we actively discuss as well. Using wild type as the control corrects for background binding to the strep column but does not correct for non-specific

labeling by TurboID. Using “free,” or citrine-fused TurboID as the control helps correct for both background binding to strep column as well as control for non-specific TurboID labeling. This point is nicely tested in a recent paper by Jiang et al in MCP (DOI: 10.1016/j.mcpro.2025.101001) where they show using TurboID-GFP as the control, even when expression is mis-matched with the TurboID-bait, works better than using a wild-type control. With that said we too have gotten nice results in unrelated projects using wild type as the control. Regarding q-value threshold, with a q ≤ 0.1 less than 10% of the significant hits are likely to be false. We have found that q ≤ 0.1 nicely balances false controlling for false positives without inflating false negatives. Testing lower q-values in previous studies eliminated many known true interactors (i.e. inflated false negatives). Thus, we think that a false discovery rate threshold of q ≤ 0.1 is appropriate.

I do think the authors should verify the interaction of potential partners that they touch upon in the manuscript with another technique such as BiFC as they have done before. At the very least this should be done for the F-Box candidate AT4G00893, which they study in more detail.

[Response] We thank the reviewer for this valuable suggestion. We agree that independent validation strengthens proximity-based interaction data, particularly for the F-box candidate AT4G00893, hereafter referred to as DMR6-ASSOCIATED F-BOX 1 (DAF1).

To address this, we performed co-immunoprecipitation (Co-IP) assays, which verified a direct physical association between DAF1 and DMR6 in planta (new Fig. 4f). In these assays, 3×myc–DAF1 efficiently pulled down 3×HA–DMR6 from N. benthamiana extracts, confirming a specific interaction consistent with F-box–substrate recognition.

We further established functional linkage through multiple independent approaches. In cell-free degradation assays, DMR6 and DLO1 were degraded more slowly in daf1 mutant extracts than in wild type (Fig. 4g–h), and co-expression of DAF1 reduced DMR6 reporter fluorescence in a ratiometric degradation assay (new Supplementary Fig. 9b), supporting that DAF1 promotes DMR6 turnover. Immunoprecipitation followed by anti-Ub immunoblotting revealed reduced DMR6–Ub conjugates in *daf1* (new Supplementary Fig. 9d), indicating impaired ubiquitination in the absence of DAF1. While DAF1 functions as an E3 ligase adaptor predicted to promote DMR6 degradation, steady-state DMR6 protein remained low in *daf1* (Fig. 4i; Supplementary Fig. 9c), reflecting a self-regulatory feedback loop within the SA–DMR6–DAF1 network. SA accumulation induces DMR6 transcription, while DAF1-mediated turnover limits DMR6 abundance; loss of DAF1 disrupts SA catabolism and attenuates DMR6 transcriptional induction, leading to reduced steady-state protein levels despite impaired degradation. Genetic and metabolic analyses reinforce this model: *daf1* mutants show reduced 2,5-DHBA accumulation and enhanced resistance to Pst DC3000, consistent with diminished DMR6 activity and stabilized SA signaling (new Fig. 4j–k; Supplementary Fig. 9f–i). Conversely, DAF1 overexpression triggered leaf necrosis that is likely SA-dependent, as shown by suppression of this necrosis by co-expression of DMR6 or the bacterial salicylate hydroxylase NahG (new Fig. 4l; Supplementary Fig. 10a–c).

These extensive new data are now presented in an entirely new Results section, supported by new main (Fig. 4) and supplementary (Figs. 9–10) figures. Collectively, they provide independent biochemical, genetic, and physiological validation of the DMR6–DAF1 association, establishing DAF1 as a bona fide F-box component of an SCF-type E3 ligase that regulates DMR6 stability

and SA-dependent immunity. This comprehensive addition fully addresses and substantially strengthens the reviewer's original request for functional verification of the TurboID-identified interactor.

Comments to section 4 of the Results.

This section seems somewhat detached from the story and comes across like an add-on although a substantial one. I would remove it because it doesn't really add anything to the mechanistic insights of DMR6 and DLO1 mode of action in SA dependent plant defense, but instead broadens the narrative in a more or less purely descriptive manner. It may form the basis of another paper.

[Response] We thank the reviewer for this constructive observation and agree that the ASK1–TurboID dataset provides broader, systems-level context that extends beyond the immediate DMR6 and DLO1 function. Our rationale for including this section was to explore whether the SCF complex environment, which mediates ubiquitin-dependent proteolysis, is dynamically reorganized during pathogen-induced SA signaling, thereby influencing DMR6 and DLO1 turnover. To clarify this connection, <u>we have revised the text to explicitly</u> explain the motivation for this analysis. The revised paragraph now reads:

"*While DAF1 may contribute to the proteolysis of DMR6 and DLO1 under basal conditions, pathogen infection may further influence their abundance and regulatory interactions. In particular, Pseudomonas syringae pv. tomato (Pst DC3000)-induced SA accumulation and immune signaling could modulate SCF complex composition, altering target recruitment and degradation. These observations prompted us to examine how DMR6 protein levels and ASK1-associated proximity networks are reshaped during bacterial infection, providing insight into the context-dependent regulation of SA catabolic enzymes*."

We believe this integration strengthens the narrative by positioning the ASK1 interactome as a mechanistic bridge between E3 complex remodeling and substrate regulation during immunity. The revised section (now under Results) is directly linked to the main theme of SA-dependent DMR6 and DLO1 regulation and highlights how proteasomal control of these enzymes is embedded within dynamic SCF network remodeling under biotic stress.

1. At this point, after having verified the interaction of AT4G00893 with DMR6 and DLO1 as requested above, DMR6 and DLO1 ubiquitylation, abundance should be monitored in the AT4G00893 mutant background after SA treatment and PstDC300 infection with targeted mass spectrometry approaches as explained under comments to section 1.

[Response] We agree that quantitative assessment of DMR6 and DLO1 abundance and ubiquitylation in the AT4G00893 mutant background under SA and pathogen challenge provides important mechanistic insight. <u>We have addressed this point extensively in the revised version through multiple complementary approaches that provide equivalent mechanistic and quantitative information. In the revised Figure 4-5 and Supplementary Figure 9-10, we now show</u> DMR6 protein levels in wild-type and *daf1* plants following SA treatment with or without MG132, as well as under basal and pathogen-infected conditions. These experiments reveal that DMR6 remains only faintly detectable in *daf1*, with limited response to MG132 (Fig. 4i), and that polyubiquitinated endogenous DMR6 species are reduced in *daf1* seedlings (Supplementary Fig. 9c–d). These results demonstrate that DAF1 loss affects both DMR6 ubiquitination and steady-state

abundance. We further expanded our analyses to include ASK1-associated proximity labeling under *Pst DC3000* infection, which revealed infection-dependent remodeling of SCF networks and changes in DMR6 association consistent with dynamic regulation during immune activation.

While we appreciate the value of targeted MS, this approach is technically challenging in our system because DMR6 and DLO1 are expressed at extremely low endogenous levels, particularly under native promoters and transient infection conditions. Generating labeled internal standards or achieving sufficient ion intensity for reproducible quantification would require extensive method development. Moreover, our ratiometric fluorescence degradation assay introduced in this revision (Fig. 1g, Supplementary Fig. 9b) already provides precise, quantitative measurement of DMR6 turnover in planta with temporal resolution that would be difficult to capture using targeted MS. Thus, while targeted MS would be an excellent complementary approach, we believe that the combination of quantitative immunoblotting, ratiometric assays, and infection-dependent analyses provides robust mechanistic insight fulfilling the intent of the reviewer's suggestion. To further explore this point, we repeated the SA + MG132 treatment in wild-type and *daf1* side-by-side, confirming that DMR6 protein remains lower in *daf1* despite attenuated proteasomal degradation (Supplementary Fig. 9c). This finding, although counterintuitive at first glance, highlights a self-limiting feedback mechanism that balances DMR6 synthesis and degradation. Specifically, SA promotes DMR6 transcription (as shown previously, e.g., Zhang et al 2017) but also triggers DMR6 proteasomal degradation, potentially through SA-dependent conformational modulation of DMR6 that facilitates DAF1 recognition (which we provide deep structural analysis as new Figure 2, S4-5). Depletion of DMR6 protein allows SA to re-accumulate, re-inducing DMR6 expression and restoring balance. In the absence of DAF1, however, SA catabolism becomes less controlled, leading to overall SA depletion, weaker DMR6 transcriptional induction, and consequently lower total DMR6 protein despite reduced degradation.

Such self-regulatory feedback loops are well established in plant signaling. Analogous paradigms include: Ethylene signaling (EIN3/EBF2 loop): EIN3 activates EBF2, an F-box protein that ubiquitinates EIN3, thereby terminating the signal (Guo & Ecker, Cell 2003; Potuschak et al., Cell 2003); Auxin signaling (Aux/IAA–SCF-TIR1 loop): Aux/IAAs repress auxin-responsive transcription, including their own genes; when degradation is blocked, their mRNA and protein both decline (Gray et al., Nature 2001; Ramos et al., Nature 2001).
By analogy, DMR6 and DAF1 likely constitute an SA-responsive feedback module in which SA induces both DMR6 transcription and DAF1-mediated degradation, thereby maintaining SA homeostasis through coupled transcriptional and proteolytic control.
Needless to say, this discovery opens an exciting new avenue that we plan to explore in future studies, including deeper phenotypic analysis of DAF1 function, structural and biophysical investigation of how SA influences DMR6 conformational dynamics and E3 ligase recruitment, and examination of whether similar feedback architectures operate among other hormone-catabolic enzymes.
Together, these biochemical, infection-dependent, and mechanistic analyses provide quantitative support for DAF1-mediated regulation of DMR6 and DLO1, fulfilling the reviewer's request through multiple orthogonal approaches. The new results also reveal a novel feedback mechanism that integrates SA signaling, transcriptional induction, and proteasomal regulation, offering deeper mechanistic insight than targeted MS quantification alone could provide.

2. PstDC3000 infection assays (bacterial recovery) in the DMR6, DLO1 and AT4G00893 mutant backgrounds should be included to show that these proteins directly affect plant defense. These experiments were described in the materials and methods section, but I couldn't find anything relating to them in the results.

[Response] We thank the reviewer for this valuable comment and agree that bacterial recovery assays in the *dmr6, dlo1*, and *daf1* mutant backgrounds are critical to demonstrate their contribution to pathogen defense. Previous studies have already reported detailed *Pseudomonas syringae pv*. tomato (*Pst DC3000*) infection assays for *dmr6* (also referred to as *s5h*), *dlo1* (*s3h*), and the *dmr6 dlo1* double mutant (Zhang et al., 2017 , see reference figure). These analyses showed that *dmr6* and *dmr6 dlo1* mutants accumulate higher levels of SA and exhibit significantly reduced bacterial growth compared to wild type, confirming their roles in promoting pathogen susceptibility. We have now cited this explicitly in the Introduction to provide context for their function in immunity. In our study, bacterial recovery assays were initially conducted to verify uniform inoculum application and uptake across the stable TurboID lines used for ASK1 proximity labeling (previously described in the Methods and Supplementary Information). We recognize that this was not clearly stated in the Results and have now added a sentence clarifying this point:

"*Bacterial recovery assays confirmed that all genotypes received an equivalent amount of inoculum and supported equal Pst uptake and proliferation during the first 24 hours of infection (Supplementary Fig. 11a).*"

To directly address the reviewer's suggestion, we performed new bacterial recovery assays in the *daf1* T-DNA mutant (SALK_072882). These results are now presented in Figure 4j and Supplementary Fig. 9h–i, showing significantly reduced bacterial growth and lesion formation in daf1 relative to Col-0. The experiment included 11 biological replicates across three independent trials, with CFUs quantified as mean ± SD.

We further clarified this connection in the Discussion, emphasizing that the observed daf1 resistance phenotype aligns with previously reported dmr6 and dmr6 dlo1 mutant responses and reinforces that proteasomal regulation of DMR6 via DAF1 contributes directly to pathogen susceptibility. Together, these additions clearly link DMR6, DLO1, and DAF1 to SA-dependent plant defense, demonstrating that all three genes influence immunity through interconnected metabolic and proteolytic regulation.

[editorial note: third party material redacted]

Fig. R3 for reviewer (Zhang et al, 2017).

3. How do the authors reconcile exogenous SA dependent degradation of DMR6 with PstDC3000 dependent stabilization of DMR6 when PstDc3000 infection is known to lead to a rapid and substantial increase in SA levels? Measurements of SA and hydroxylation products in these infection scenarios should be performed to shed light on this.

[Response] We thank the reviewer for highlighting this important point regarding the apparent discrepancy between exogenous SA–accelerated degradation of DMR6 and the *Pst DC3000*–associated increase in DMR6 protein, even though Pst infection is known to elevate SA levels (Block et al., 2005; Spoel et al., 2003; Tsuda et al., 2008). We agree that this distinction is mechanistically important and have clarified it explicitly in the revised Results and Discussion.

Our interpretation is that pathogen-induced and exogenous SA treatments differ substantially in temporal dynamics and regulatory context, leading to distinct outcomes for DMR6 stability. Exogenous SA provides a uniform and acute increase in SA concentration that can directly promote DMR6 conformational changes, facilitating DAF1-mediated proteasomal degradation under controlled experimental conditions (Fig. 1). In contrast, Pst DC3000 infection triggers a complex immune signaling network involving not only SA accumulation but also strong transcriptional activation of DMR6, redox changes, MAPK signaling, and remodeling of the SCF ubiquitin ligase network. In this infection context, transcriptional upregulation and post-translational stabilization of DMR6 likely outweigh the degradation component observed under exogenous SA treatment, leading to apparent DMR6 accumulation during infection. This interpretation is consistent with previous transcriptomic and biochemical studies showing that *Pst DC3000* infection robustly induces DMR6 transcription (Zhang et al., 2017- see **fig. R4** for reference), and with prior quantification of SA hydroxylation products during infection that demonstrated increased 2,5-DHBA and 2,3-DHBA accumulation (Zhang et al., 2017). These data imply that DMR6 activity rises during infection, consistent with our observation of higher DMR6 protein abundance in infected tissue. Thus, while exogenous SA can accelerate DMR6 degradation under steady-state conditions, pathogen-induced SA accumulation occurs alongside strong DMR6 transcriptional induction and immune pathway activation, which together promote net DMR6 accumulation.

[editorial note: third party material redacted]

Fig. R4 for reviewer (Zhang et al, 2017).

We have now clarified this point in both the Results and Discussion sections. Specifically, we added the following statement in the Discussion:

"*The apparent stabilization of DMR6 during Pst DC3000 infection, despite high endogenous SA levels, can be explained by the interplay between transcriptional induction and post-translational regulation. Whereas exogenous SA application imposes an acute, uniform stimulus that promotes DMR6 proteasomal degradation through DAF1-mediated turnover, pathogen infection triggers a broader immune network involving SA synthesis, transcriptional activation of DMR6 and DAF1, and dynamic remodeling of the SCF complex. In this immune context, transcriptional upregulation of DMR6 likely exceeds the rate of proteasomal degradation, resulting in net accumulation of DMR6 protein. Similar patterns have been reported in transcriptomic and metabolite analyses, where Pst DC3000 infection leads to elevated DMR6 mRNA and increased 2,5-DHBA levels (Zhang et al., 2017). Together, these findings highlight that SA regulates DMR6 through multiple layers, transcriptional, conformational, and proteolytic, that can be differentially weighted depending on the physiological and immune context*."

Together, these clarifications reconcile the infection- versus treatment-dependent DMR6 behavior and further underscore that SA modulates DMR6 turnover through a multifactorial network involving transcriptional activation, conformational control, and SCF-mediated proteolysis.

Comments to figure 5 (model).

The model should be expanded according to incorporate results of the additional experiments. For instance, ubiquitylation should be added before degradation when it has been measured. Also, AT4G00893 and the results of the patho-assays could be added.

[Response] We thank the reviewer for this helpful suggestion. We have revised Figure 5 (now new Figure 6) to incorporate the experimental data and to more clearly depict the regulatory logic supported by our results. The revised schematic now places ubiquitination upstream of proteasomal degradation, explicitly includes the putative F-box protein DAF1 (AT4G00893) as the adaptor within the E3 ligase SCF complex, and summarizes context-dependent effects under SA treatment and pathogen infection. Insets summarize the daf1 mutant phenotypes (reduced DMR6–Ub conjugates, lower 2,5-DHBA, and enhanced *Pst DC3000* resistance). The figure legend and Discussion have been updated accordingly. We propose a self-limiting regulatory loop in which SA promotes DMR6 transcription, as previously demonstrated, while simultaneously triggering DMR6 proteasomal degradation, likely through SA-induced conformational changes that facilitate E3 ligase recruitment. Depletion of DMR6 by proteasomal turnover allows SA to re-accumulate to a certain threshold, which then re-induces DMR6 transcription and synthesis of new DMR6 protein. In the absence of DAF1, DMR6 remains more active and inactivates SA less selectively, resulting in gradual SA depletion and diminished DMR6 transcription. Consequently, although DMR6 protein is more stable in terms of reduced degradation, its overall abundance decreases due to attenuated transcriptional induction.

Similar self-regulating feedback loops occur in other hormone signaling pathways. In ethylene signaling, the transcription factor EIN3 activates the F-box EBF2, which ubiquitinates EIN3 and terminates the signal; EIN3 is also required for EBF2 transcription (Guo & Ecker, Cell 2003; Potuschak et al., Cell 2003). In auxin signaling, Aux/IAA proteins repress auxin-responsive transcription, including their own genes, and are normally short-lived via SCF-TIR1 -mediated ubiquitination; in degradation-impaired mutants, hyperstable Aux/IAAs suppress auxin signaling and repress their own transcription (Gray et al., Nature 2001; Ramos et al., Nature 2001). By analogy, our model provides a mechanistic framework in which SA-induced conformational dynamics of DMR6 facilitate its ubiquitination and turnover by DAF1, forming a feedback circuit that maintains SA homeostasis and prevents excessive immune activation. The revised Figure 6 and legend now reflect this integrated and cautious view.

Reviewer #2

This manuscript investigated the regulation of the SA hydroxylases DMR6 and DLO1 by proteosome-mediated degradation. The authors found that SA binding triggers conformational changes in DMR6, particularly in the conserved C-terminal helix. These changes make DMR6 susceptible to degradation. The DLO1 C-terminal helix is less conserved, and SA binding-induced conformational change is not significantly, As the result, SA induces DMR6 degradation but stabilizes DLO1. Using Turbo-ID proximity labeling, the authors found a putative F-box protein that appears to be involved in DMR6 and DLO1 degradation and that DC3000 infection induces the ASK1-SCF proxeome reprogramming.

Major comments*:*

1. The authors tested DMR6 and DLO1 degradation under multiple conditions. One is cell-free or in vitro degradation assay. Figure 1g,h and Supplementary Figure 2a,b are cell-free or in vitro degradation. His-DMR6 degradation is much faster in the Supplementary Figure. It seems the authors used EtOH to dissolve SA and water to dissolve 2,5-DHBA. Is the reduced degradation in Figure 2a,b caused by EtOH? The authors may need to use sodium salicylate in water to accurately determine the half-life of the protein.

[Response] We thank the reviewer for raising this important methodological point. The apparent difference in degradation rate between panels likely reflects minor variability among extract preparations and detection conditions rather than solvent effects. All direct comparisons were performed within the same membrane to ensure internal consistency, and replicate experiments yielded the same relative trends. Following established protocols (Plant Physiology 2005, doi.org/10.1104/pp.105.059303; J. Exp. Botany 2016, doi.org/10.1093/jxb/erv468*), SA was dissolved in ethanol at a final concentration of 0.01%, a level far below that known to influence proteasome or enzyme activity. To directly address the reviewer's suggestion, we repeated the assay using SA dissolved either in ethanol or as sodium salicylate in water.

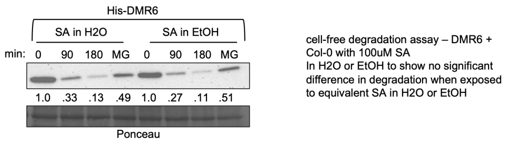

Incorporated in new supplementary figure 13

The resulting DMR6 degradation kinetics were indistinguishable (new Supplementary Fig. 13), confirming that solvent composition does not affect DMR6 stability.

The Methods now read:

"*In all experiments, SA was dissolved in ethanol as previously described; parallel assays using sodium salicylate in water yielded indistinguishable results.*"

2. Since SA is a substrate of DMR6, it is not surprising that SA binding causes conformational changes in DMR6, affects trypsin-limited proteolysis, and makes DMR6 more susceptible to proteosome-mediated degradation. Although defining the exact conformational change at the CTH is significant, it does not explain the underlying mechanism.

[Response] We thank the reviewer for highlighting the need to clarify the mechanistic role of the C-terminal helix (CTH) in DMR6 regulation. Our data support that the CTH contributes to recognition by the E3 ligase rather than solely mediating catalytic dynamics. The CTH-swap experiments in the revised Fig. 3 show that replacing the DMR6 CTH with that of DLO1 abolishes the SA-induced limited proteolysis sensitivity observed in wild-type DMR6, whereas inserting the DMR6 CTH into DLO1 accelerates its degradation but does not confer SA responsiveness (Fig.

3f–h – previously Fig. 2). These results identify the DMR6 CTH as a transferable determinant of proteasomal sensitivity, while full SA-dependent regulation likely requires cooperative structural elements beyond the helix itself. In the revised manuscript, we have also expanded our structure-based analysis, combining molecular docking and molecular dynamics simulations, which further corroborate the SA-induced conformational change and support its functional relevance for E3 recruitment (new Fig. 2, Figure S4-S5, S9). We have also carried out substantial new work in planta to characterize the association between DMR6 and its cognate E3 ligase.

We agree that the precise molecular mechanism linking CTH remodeling to E3 engagement will require additional structural studies, particularly in complex with the putative F-box protein DAF1. To clarify this point, the Discussion now emphasizes that future crystallographic or cryo-EM analyses will be needed to define how SA-induced rearrangements expose degron-like surfaces for E3 recognition.

Importantly, our revised text situates this mechanism within a broader paradigm of hormone-dependent conformational gating. In strigolactone signaling, ligand-induced rearrangements of the receptor D14 promote interaction with the F-box protein MAX2, leading to receptor ubiquitination and degradation (Yao et al., Nature 2016; Shabek et al., Nature 2018; Tal et al., Nature Plants 2020). Analogous ligand-triggered plasticity has been described for the strigolactone-specific carboxylesterases CXE15 and CXE20, which undergo distinct conformational changes upon catalysis (Palayam et al., Nature Comm 2024). We have incorporated these parallels to highlight that DMR6 likely represents a similar structural module in which SA binding induces local remodeling, particularly at the CTH, that enhances accessibility for DAF1-mediated ubiquitination.

These revisions clarify that while the CTH is a determinant of SA-responsive degradation, full E3 engagement likely involves cooperative allosteric changes extending beyond this region. The revised Discussion explicitly outlines this model and positions DMR6 within a conserved mechanistic framework of metabolite-driven substrate recognition by SCF-type E3 ligases.

3. Using TurboID proximity labeling, the authors identified a Kelch-domain F-box protein and provided evidence that the Arabidopsis ortholog is involved in DMR6 degradation. F-box protein is known to bind to substrate and the authors also predicted an interface between the Kelch domain and DMR6 (Supplementary Figure 7b). SA binding may enhance the interaction between the Kelch domain and DMR6, which would explain the observed result. This needs to be tested, since the authors have all the materials in hand.

[Response] We thank the reviewer for this constructive suggestion. In the revised manuscript, we have carried out substantial new work to characterize the association between DMR6 and the Kelch-type F-box protein AT4G00893, which we now designate DAF1 (DMR6-Associated F-box). Using complementary biochemical, genetic, and in planta approaches, we collectively establish DAF1 as a major E3 component mediating DMR6 degradation:

(1) Protein–protein interaction. Co-immunoprecipitation experiments demonstrate that DAF1 physically associates with DMR6 when co-expressed in *N. benthamiana*, confirming their in planta interaction (new Fig. 4f).

(2) Functional role in proteasomal turnover. Cell-free degradation assays using extracts from daf1 mutants reveal markedly slower degradation of both DMR6 and DLO1 relative to wild type (new Fig. 4g,h). In complementary in planta assays, MG132 treatment partially stabilizes endogenous DMR6 in the *daf1* background (Fig. 4i), consistent with DAF1-mediated proteasomal targeting. Immunoprecipitation of endogenous DMR6 from Arabidopsis seedlings followed by anti-ubiquitin blotting further shows a modest reduction in DMR6–Ub conjugates in daf1 extracts (new Supplementary Fig. 9d).
(3) Quantitative in planta degradation. Ratiometric Venus–mScarlet assays in *N. benthamiana* reveal that co-expression of DAF1 significantly accelerates DMR6 degradation compared to controls (new Supplementary Fig. 9b), providing quantitative evidence of DAF1-dependent turnover.
(4) Physiological relevance. *daf1* mutants display reduced 2,5-DHBA accumulation (LC–MS/MS) and enhanced resistance to *Pst DC3000* infection (Fig. 4j,k; Supplementary Fig. 9f–i), consistent with attenuated DMR6 function. Conversely, DAF1 overexpression induces cell death in *N. benthamiana* leaves, which is suppressed by co-expression of *DMR6* or the bacterial salicylate hydroxylase NahG (new Fig. 4l; new Supplementary Fig. 10a–c). These in planta assays collectively demonstrate that DAF1 regulates DMR6 stability and SA-dependent immune homeostasis. We have further elaborated this regulatory logic in the Discussion, proposing a self-limiting SA–DMR6–DAF1 feedback loop, where SA promotes *DMR6* transcription and conformational remodeling while DAF1-mediated proteasomal degradation prevents excessive SA catabolism.

While much remains to be explored regarding the precise sequence of molecular events and structural determinants of DMR6–DAF1 engagement, the scope of this study establishes a novel framework for E3-mediated turnover of a key enzyme in SA inactivation. We propose that this represents part of a broader mechanism through which plants fine-tune immune responses by coupling hormone metabolism to proteasome-dependent regulation.

4. The regulation of in vivo DMR6 degradation is more difficult to dissect, since pathogen infection increase DMR6 protein levels. This is likely due to increased gene expression, as the authors proposed. On the other hand, CHX and MG132 treatments (Figure 1k,l, it is not clear if SA was added here) indicate that DMR6 is intrinsically unstable in the absence of SA treatment (if SA was not added). The D214A mutant is stable, indicating this mutation could break the DMR6-F-box protein interaction. This should be tested as well.

[Response] We thank the reviewer for their insightful comments and agree with their interpretation of the data. As clarified in the revised manuscript, no exogenous SA was added in the CHX and MG132 assays (Fig. 1h–k). These experiments were designed to assess basal proteasomal turnover of DMR6. It is important to note, however, that low endogenous levels of SA are present in most plants, which cannot be easily or fully eliminated. Indeed, as shown by ours and others LC–MS/MS analyses, measurable amounts of SA are detected even in untreated plants, consistent with the basal presence of most phytohormones. This factor should be considered when interpreting CHX and MG132 experiments, as DMR6 instability under “untreated” conditions may still reflect low-level SA-dependent effects.

With regard to the D214A mutant, this residue lies within the catalytic pocket, coordinating the Fe(II) cofactor, and substitutions here are known to disrupt DMR6 enzymatic activity. Our data indicate that catalytic activity contributes to DMR6's rapid degradation, reminiscent of the D14 receptor in strigolactone signaling, where catalysis facilitates receptor turnover. However, degradation of the D214A mutant is not completely blocked, suggesting that proteasomal targeting can still occur in the absence of catalysis. Because D214 is buried within the active site and not solvent-exposed, it is unlikely to directly mediate interaction with the E3 ligase. Indeed, both in planta and in vitro assays show that the mutant remains subject to proteasomal degradation, indicating that E3 recruitment still occurs, albeit less efficiently than in wild-type DMR6.

Based on these observations, we propose that SA-induced structural plasticity facilitates efficient recognition of DMR6 by the F-box protein DAF1, while catalytic turnover promotes conformational states favorable for degradation. Nevertheless, even when catalysis is impaired, DMR6 can likely still be recruited, albeit less efficiently, by the same or potentially alternative E3 ligases. While we agree that direct D214A–DAF1 interaction assays would be informative, the results may be difficult to interpret because recruitment and degradation are partially independent of catalytic function. We have now expanded the *Discussion* to explicitly address these considerations, emphasizing that DMR6 degradation is governed by the combined effects of basal SA levels, catalytic activity, and conformational flexibility, all of which converge on DAF1-mediated proteasomal turnover.

5. NPR1 is known to suppress SA accumulation by repressing ICS1 expression. It would be interesting to test if SA signaling instead of SA per se is the enhancer of DMR6 degradation. This could be tested using the DMR6 antibody and npr1 mutants.

[Response] We thank the reviewer for this comment. As suggested, we tested DMR6 protein levels in the *npr1-1* mutant using our endogenous DMR6 antibody. The results, now included in the revised manuscript (Supplementary Fig. 9e), show that DMR6 protein abundance is drastically reduced in *npr1-1* compared to wild type, indicating that SA-dependent signaling through NPR1 contributes to DMR6 regulation. This finding supports the idea that SA signaling, rather than SA accumulation alone, promotes DMR6 turnover, likely by coupling transcriptional activation with post-translational degradation. We have added a statement in the *Discussion* explicitly addressing this point:

"*Reduced DMR6 abundance in the npr1-1 mutant suggests that SA signaling through NPR1, and not merely SA levels, contributes to DMR6 regulation, consistent with coordinated transcriptional and proteasomal control within the SA–DMR6–DAF1 feedback loop*."

Together, these results strengthen the proposed model in which SA signaling integrates transcriptional induction and E3-mediated degradation to maintain immune homeostasis.

Minor Comments*:*

1. Supplementary Figure 1c, at first glance, it was surprising to see the size difference between 6xHis-DMR6 and untagged DMR6. The figure legend says it is actually 6xHis-MysB-DMR6.

[Response] We agree that this figure, which was included to validate the antibody, appeared misleading. We apologize that the annotation was incomplete: the construct labeled 6×His-DMR6 carries a longer 6×His-MsyB fusion tag that provides a necessary solubilization component for recombinant protein expression. This has been corrected and clarified in the corresponding figure legends and Methods. During this revision, we also identified a few additional instances where construct annotations could be more clearly illustrated and corrected throughout the figures and text for consistency.

2. Figure 1k,l, the basal level of DMR6-3xHA in (l) is extremely low compared to that in (k).

[Response] We thank the reviewer for bringing this up. We agree that the basal level of DMR6-3×HA in Fig. 1l appears lower than in Fig. 1k (now Figures 1 h and i in the revision) and other time = 0 experiments. This difference reflects the distinct experimental design of the two assays. In Fig. 1l, DMR6 is shown side by side with MG132-treated tissue; inhibition of the proteasome leads to strong accumulation of DMR6, making the basal (steady state levels), untreated sample appear faint by comparison. This behavior is consistent with the rapid turnover of DMR6 under normal conditions. In contrast, Fig. 1k monitors DMR6 stability following a cycloheximide chase, which halts de novo protein synthesis and tracks the decay of pre-existing protein pools. We have clarified these distinctions in the Results section and figure legend, and we have also added additional annotations throughout the figures to specify treatment conditions and time points more clearly.

3. Supplementary Figure 5a, Myc signal is missing in some samples?

[Response] We agree with the reviewer and have indeed observed similar challenges in visualizing Myc-tagged TurboID fusion proteins across multiple proximity-labeling experiments over the years. These difficulties likely stem from fluctuations in TurboID expression, normalization during the western blotting process (as only small protein inputs, typically <5 µg, are used to avoid signal oversaturation when detecting biotinylated proteins with Strep–HRP), or partial masking of the Myc epitope in overexpressed TurboID fusions. Importantly, the dataset remains fully validated, as cis-biotinylation of DMR6 is clearly observed, confirming the integrity and activity of the TurboID fusion. Nevertheless, we agree that this particular western blot shows a relatively weak Myc signal. To address this, <u>we have added an additional panel</u> (Supplementary Fig. 7a) showing protein expression side by side, confirming that the Myc-tagged protein was present in the extract but expressed at a relatively low level. This clarification has been incorporated into the revised figure legend.

4. Figure 3h, what happened in wild type?

[Response] We thank the reviewer for this question. The main purpose of this panel (now new Fig. 4i) was to characterize the impact of the F-box mutant by assessing steady-state DMR6 levels in the daf1 background. Endogenous DMR6 levels were previously shown in wild-type (Col-0) in Fig. 1 for reference. To address this point more explicitly, we have now repeated the experiment side by side with Col-0, as shown in new Supplementary Fig. 9c. Consistent with our model, we observed a pronounced decrease in DMR6 protein levels in the *daf1* mutant compared with wild type, further supporting the proposed self-limiting SA–DMR6–DAF1 feedback loop described in the Results and illustrated in the updated figures.

5. Supplementary Figure 7b, is the CTH required for DMR6-Kelch interaction if it is a degron-like module recognized by UPS as the authors proposed?

[Response] We thank the reviewer for this question. Our data indicate that the C-terminal helix (CTH) contributes to, but is probably not solely required for, DMR6 degradation or E3 engagement. The CTH-swap experiments (Fig. 3d–h) show that replacing the DMR6 CTH with that of DLO1 abolishes the SA-induced limited proteolysis sensitivity observed in wild-type DMR6, whereas inserting the DMR6 CTH into DLO1 accelerates its degradation but does not confer SA responsiveness. These findings suggest that while the DMR6 CTH enhances proteasomal targeting, additional regions of DMR6 are required for full SA-dependent responsiveness. In the revised manuscript, <u>we also performed protein-protein docking simulations to explore the predicted DMR6–Kelch interaction (new Fig. S10)</u>. While these remain predictive models in the absence of experimental structures, the analyses suggest that the Kelch domain preferentially docks near the DMR6 C-terminal region. However, simulations using the CTH alone did not reproduce the full interface, implying that surrounding residues contribute to stable binding. We therefore interpret the DMR6 CTH as a contributing but not autonomous degron-like element, whose recognition is enhanced by SA-induced conformational dynamics. Alternatively, the accelerated degradation of the DLO1–CTH chimera may reflect the loss of stabilizing features native to DLO1. <u>Our updated structural model also incorporates DAF1 as part of the SCF complex, showing an orientation in which the DMR6 C-terminal region faces the Kelch domain and lies proximal to the E2–ubiquitin module, consistent with a substrate poised for ubiquitination</u>. We consider this a working model that will guide future experimental work, particularly structural and biochemical analyses, to define the precise architecture of the DMR6–DAF1–SCF complex. We are eager to pursue these follow-up studies to fully elucidate the sequence of molecular events underlying this regulatory mechanism.

Reviewer #3

Shabek and co-workers studied dual-scale regulatory mechanism for maintaining salicycilic acid homeostasis. The work is mainly focused on biochemical experiments and uses molecular dynamics simulation for supporting the experimental findings. I was specifically asked to review the molecular dynamics simulations reported in this manuscript. I have major concerns about the MD part of this manuscript, which are included below. The manuscript could have benefited from a collaboration with a MD expert who could have easily addressed several critical simulation setup issues with the current work. I believe that this is an excellent manuscript which could be published after major revisions to the molecular modeling section.

[**Response**]: We thank Reviewer 3 for the detailed and expert evaluation of the MD component of our study. We have substantially expanded and refined the MD analyses in response to these comments, including the use of the CHARMM36m force field, replicate simulations, targeted structural metrics, and new docking analyses. While the MD analysis was not intended as a central pillar of this work, it serves as a complementary, hypothesis-driven framework that supports and rationalizes our experimental observations. While we found the additional computational work insightful and informative, it did not alter our initial observations or hypotheses; rather, it reinforced and contextualized them within a more comprehensive mechanistic framework. The revised MD section now meets the technical standards and provides consistent, reproducible results; however, the overall conclusions of the study remain fully supported even in its absence.

1. Choice of Force Field: The authors have employed the CHARMM36 force field for their simulations. However, CHARMM36m is an improved and widely adopted version that provides better backbone sampling, particularly for intrinsically disordered regions and proteins. Can the authors justify their choice of CHARMM36 over CHARMM36m? Given the improvements and widespread use of CHARMM36m in recent literature, I strongly recommend that the authors consider repeating key simulations using CHARMM36m and compare the results with their current findings.

[**Response**]: We thank the reviewer for this important suggestion. To address this concern, we have regenerated the system inputs using the CHARMM-GUI web interface with the CHARMM36m force field and repeated the molecular dynamics simulations in triplicates using GROMACS 2023.3 version. The results obtained with CHARMM36m are in very good agreement with our original CHARMM36 simulations, and importantly, all of our main conclusions remain unchanged. We have updated the Methods section to clearly indicate the use of CHARMM36m and have included the corresponding results in the revised manuscript.

2. Choice of Barostat: Authors have used Berendsen barostat for their simulations. It is a widely used barostat available in the GROMACS package. Several studies have shown that the Berendsen barostat gives incorrect ensembles. Therefore, I would advise authors to change the barostat in their simulations. Please refer to (1) J. Chem. Theory Comput., 2013, 9, 2, 909–926; (2) J. Chem. Theory Comput. 2015, 11, 12, 5583–5597 for more information on the issues related to Berendsen barostat.

[**Response**]: We thank the reviewer for this important comment and apologize for the lack of clarity in the Methods. To clarify, all NPT equilibration and production simulations were carried out with the Parrinello–Rahman barostat, which samples the correct NPT ensemble and yields proper volume and pressure fluctuations. Specifically, production runs used Parrinello–

Rahman pressure coupling (isotropic, τ_p = 5 ps, compressibility = 4.5×10^{-5} bar^{-1}) together with a v-rescale thermostat (τ_t = 1 ps), PME electrostatics, and Verlet cutoffs. We have revised the Methods to state these settings explicitly.

3. Simulation Replicates: For robust statistical interpretation and to account for stochastic variability in MD, it is standard practice to run at least three independent replicates for each condition. If replicates were not performed, I recommend conducting them and using the results to estimate statistical errors for all major analyses. This will help ensure that the observed trends are reproducible and not artefacts of a single trajectory.

[**Response**]: We thank the reviewer for this valuable suggestion. To address this point, we have performed three independent 100 ns GROMACS simulations for each condition (DLO1-apo, DLO1–SA bound, DMR6-apo, and DMR6–SA bound). All analyses presented in the manuscript are based on these triplicates, using charmm36m forcefield and the reported values include statistical errors derived from them. This ensures that the observed conformational trends are robust, reproducible, and not artifacts of a single trajectory.

4. Simulation Length: The simulations are limited to 100 ns, which may not be sufficient to capture slow or large-scale conformational changes. Recent studies commonly use simulation timescales in the microsecond regime, often discarding the initial few hundred nanoseconds as equilibration. Can the authors justify why 100 ns was considered sufficient in this context? If computationally feasible, extending the simulations would strengthen the conclusions drawn from the data.

[**Response**]: We acknowledge that microsecond-scale simulations can provide additional insights into slow or large-scale conformational changes. However, due to computational limitations, it was not feasible for us to perform microsecond-long simulations in triplicates for all systems. Instead, we performed 100 ns simulations in triplicate for each condition (DLO1-apo, DLO1–SA bound, DMR6-apo, and DMR6–SA bound). This approach allowed us to obtain statistically reproducible results while balancing available computational resources. Importantly, within the 100 ns timescale, the systems reached equilibrium and the observed conformational differences between apo and ligand-bound forms were consistent across replicates, supporting the robustness of our conclusions.

5. Ligand Stability and Pose: The initial ligand pose appears to be selected based on docking scores. However, Fig. 2c and Fig. 2f suggest that the ligand may not remain stably bound in that configuration. I recommend that the authors include ligand-specific RMSD plots over time to track deviations from the initial docking pose. Additionally, performing longer simulations or starting from alternative binding poses could provide more insight into ligand binding stability and dynamics. This would also address concerns regarding the validity of the initial configuration used in the simulations of the SA-bound state.

[**Response**]: We thank the reviewer for this valuable suggestion. To address this point, we have now included ligand-specific RMSD analyses for salicylic acid (SA) bound to DMR6 and DLO1. These plots were calculated from triplicate 100 ns molecular dynamics simulations, and the mean ± SD values are shown. As shown in Fig. S4h and S5h, the ligand RMSD remained extremely low and stable throughout the trajectories (below ~0.02 nm for $DLO1^{SA}$ and ~0.03 nm for $DMR6^{SA}$), indicating that the docked poses were preserved with negligible deviation from their initial configurations. The reproducibility across independent replicates confirms that the observed binding modes are robust and not artifacts of the docking procedure. In addition, the key

hydrogen-bond and Fe^{3+}-coordination interactions observed in the initial structures remained intact during the simulations, further validating the stability of the SA-bound states.

6. Conformational Changes in DLO1 and DMR6: The authors refer to conformational changes in DLO1 and DMR6, but only provide RMSD as supporting data. RMSD primarily reflects deviation from the starting structure and does not directly quantify biologically relevant conformational changes. I suggest incorporating more targeted metrics such as inter-residue distances, domain angles, or catalytic pocket volume. Such analyses would allow clearer identification and comparison of open versus closed conformations and help better contextualize the observed dynamics between DLO1 and DMR6.

[**Response**]: We thank the reviewer for this valuable suggestion. In response, we performed additional targeted analyses to quantitatively describe conformational changes in DMR6 and DLO1 upon salicylic acid (SA) binding. For each system, we measured the distance between the C-terminal helix (CTH) and the protein core across all three replicates. The resulting CTH–core distance distributions revealed distinct and reproducible conformational behaviors. In DMR6, the apo state adopted an open conformation, whereas the SA-bound $DMR6^{SA}$ exhibited a closed conformation, reflecting the inward movement of the CTH toward the catalytic site. In contrast, DLO1 displayed the opposite behavior, $DLO1^{Apo}$ structure exhibits closed conformation, and binding of SA induced an opening of the catalytic pocket. To visualize these conformational states, we extracted representative frames corresponding to the 10th (closed) and 90th (open) percentile values of the CTH–core distance distributions. These conformations were further analyzed for catalytic pocket volume and surface area using KVFinder, confirming a reduction in pocket volume for DMR6 upon SA binding and an expansion for DLO1. Interestingly, these opposite conformational responses correlate with our in planta and in vitro degradation assays, which showed that Salicylic acid promotes proteasomal degradation of DMR6 but stabilizes DLO1 (Fig. 1). The structural closure of DMR6 upon SA binding likely reflects a catalytically competent and degradation-prone conformation, whereas the opening of DLO1 may correspond to an inactive, degradation-resistant state. Together, these CTH-Core distance, catalytic pocket volume-based analyses provide a direct, quantitative description of conformational dynamics beyond RMSD and establish a clear mechanistic connection between SA-induced structural transitions and differential proteasomal regulation of DMR6 and DLO1.

7. Analysis of the predicted alphafold structures. A detailed analysis of the AF2 predicted structures should be included in SI. Furthermore, it is well-known that these models are not able to accurately predict the side chain orientations. Therefore, the stability analysis for the Fe^{3+} ion placed in its binding site should be performed. This ion has high positive charge and I suspect that the structural changes in the protein could simply be caused by using even a slightly incorrect coordination of the ion binding residues.

[**Response**]: We thank the reviewer for this important comment. We have now included a detailed analysis of the AlphaFold2 (AF2) predicted structures for DMR6 and DLO1 in the Supplementary Information. The AF2 models were carefully evaluated for overall geometry, residue confidence (pLDDT), and active-site architecture prior to simulation setup.

To accurately position the catalytic Fe^{3+} ion, we aligned the AF2-predicted DMR6/DLO1 structure with the experimentally solved homologous 2OG-dependent oxygenase from Arabidopsis thaliana (PDB ID: 6LSV), which shares 40% structural identity with DMR6/DLO1. The Fe^{3+} ion from 6LSV was transferred into the DMR6 active site based on this structural alignment, ensuring

preservation of the canonical H–D–H metal-binding triad. The Fe^{3+}-fitted model was subsequently refined using the Protein Preparation Wizard in Schrödinger's Maestro/Glide suite, followed by restrained energy minimization to relieve any steric clashes and optimize side-chain orientations around the metal center. This protocol ensured correct coordination geometry and minimized electrostatic artifacts. Following refinement, the Fe^{3+} ion coordination remained stable throughout the molecular dynamics trajectories, with consistent distances (~0.23–0.24 nm) between Fe^{3+} and its coordinating atoms (Asp214/223 OD1–OD2 and His212/269 NE2) across all replicates (Fig. S4g and S5g). These stable coordination distances confirm that the observed conformational transitions are intrinsic to the protein–ligand system and not artifacts of Fe^{3+} misplacement. In addition, we have explicitly stated in the Methods and Supplementary Information that the Fe^{3+}-bound models were validated and energy-minimized prior to docking of salicylic acid using Schrödinger's Glide.
Together, these additional analyses and controls confirm that the Fe^{3+} ion was accurately positioned and stably coordinated throughout our simulations, and that the observed structural changes reflect biologically relevant conformational dynamics rather than model artifacts.

8. The goal of the MD simulations should be to provide atomistic insights into the mechanism of structural instability. The conclusions reported in the manuscript about structural dynamics are highly suspect given the numerous issues with the simulation setup. However, even if we take the results at their face value, the authors have merely used simulations to generate an observation but failed to provide the biophysical reason for the observation. A more detailed analysis of the simulations should be performed.
[**Response**]: We thank the reviewer for this important comment and have carefully expanded our molecular dynamics (MD) analysis to provide deeper atomistic and biophysical insights into the observed structural transitions. To ensure the robustness of our simulations, we first validated the starting models and Fe^{3+} coordination geometry. The AlphaFold2-predicted DMR6 and DLO1 structures were aligned with the experimentally solved homologous Arabidopsis thaliana 2OG-dependent oxygenase (PDB ID: 6LSV, >90% identity). The Fe^{3+} ion was positioned based on this template and the entire complex was energy-minimized using the Protein Preparation Wizard in Schrödinger's Glide, which optimized side-chain orientations and removed steric clashes. The Fe^{3+}–ligand coordination distances (0.23–0.24 nm) remained stable across triplicate trajectories, confirming proper metal geometry throughout the simulations (Fig. S4g and S5g). To move beyond qualitative observations, we performed quantitative and targeted analyses of the trajectories, including:

1. RMSD and per-residue RMSF to assess global and local stability;
2. Ligand RMSD to monitor the persistence of SA in the catalytic pocket;
3. Hydrogen-bond and Fe^{3+}-coordination distance monitoring to evaluate active-site integrity;
4. CTH–core distance distributions to quantify conformational closure or opening; and
5. Pocket-volume measurements (via KVFinder) to assess cavity compaction or expansion.

These analyses collectively revealed distinct and reproducible conformational mechanisms for DMR6 and DLO1. In DMR6, SA binding stabilized the catalytic His–Asp–His coordination network around Fe^{3+} and reduced local fluctuations of the C-terminal helix (CTH). This stabilization promoted a closed catalytic conformation characterized by reduced CTH–core distance and pocket volume. In contrast, DLO1 displayed an opposite behavior: SA binding increased CTH mobility and enlarged the catalytic pocket, consistent with an open, relaxed conformation.

These opposite conformational responses provide an atomistic explanation for the experimentally observed differences in proteasomal turnover: DMR6 adopts a compact, degradation-prone state upon SA binding, whereas DLO1 becomes more open and degradation-resistant. All revised analyses and structural interpretations have been incorporated into the main text (see updated Dynamics section, new Fig. 2a–h) and the corresponding Supplementary Information (new Supplementary Figs. S4-5).

Point-by-point response to reviewers

Reviewer #1

Remarks to the Author:

The authors have gone to some lengths to address the points I raised in my original review. Nevertheless, I am still somewhat less than enthusiastic about some of the quantitative data presented. The authors have gone to great length to explain to me why SA dependent changes in endogenous DMR6 levels are modest and difficult to quantify looking at the western blot alone (new figure 1d). However, this is actually a quintessential piece of information as it represents the physiological in plant situation which seems to be quite subtle.

We thank the reviewer for pointing this out. As mentioned, endogenous DMR6 protein levels are low and tightly regulated in planta, making subtle differences difficult to detect. Figure 1d includes representative relative band intensities below the blot to aid interpretation. We have now clarified in the text and legend that the endogenous signal is weak and that the SA-dependent change is small but consistent, as expected for a homeostatic SA-catabolic enzyme whose turnover is controlled by both transcriptional induction and proteasomal degradation. To address this limited dynamic range at steady state, the ratiometric degradation assay (Fig. 1f–h) provides a more sensitive and quantitative view of SA-dependent DMR6 turnover than western blot detection. The following paragraph was added:

RESULTS: "*Endogenous DMR6 protein levels are naturally low in Arabidopsis leaves, so SA-dependent changes are therefore small in magnitude. This reflects the physiological regulation of DMR6 in plants, in which SA both induces DMR6 transcription and promotes proteasomal degradation. As a result, the steady-state differences are subtle and difficult to detect by immunoblotting. The relative band-intensity values shown below the blot highlight this low-amplitude pattern. Due to the limited dynamic range of endogenous protein detection, we use the ratiometric Venus–mScarlet degradation assay (Fig. 1f–h) to get a more sensitive and quantitative understanding of the SA-dependent increase in DMR6 turnover*."

LEGEND: "*Endogenous DMR6 abundance is low, and SA-dependent shifts are subtle, consistent with its role as a homeostatic SA-catabolic enzyme subject to both transcriptional induction and proteasomal turnover.*"

DISCUSSION: *"We observe that endogenous DMR6 protein levels show modest yet consistent changes following SA treatment. This subtle behavior aligns with the biological role of DMR6 as a homeostatic SA-catabolic enzyme, whose abundance is regulated by a balance between rapid transcriptional activation and proteasomal degradation. Similar minor but biologically significant shifts have been reported for other hormone-regulated enzymes and transcriptional repressors, including Aux/IAA proteins and EIN3, where tight proteostatic control results in small net changes in steady-state levels while allowing large downstream effects. The modest endogenous shifts seen here therefore fit with the expected regulatory logic of this pathway. Additionally, the*

complexity of ubiquitin ligase networks suggests that DAF1 is unlikely to be the only E3 ligase involved in DMR6 turnover, which may further explain the nuanced phenotypes observed in specific assays.”

The authors added a quantitative ratiometric fluorescence assay that shows that DMR6 is degraded more rapidly dependent on SA whereas there is no effect on DMO1. The original cell free degradation assays have been moved to the supplements, more rapid degradation of DMR6 dependent on SA remains inconclusive to me from this figure, indeed I would doubt that the changes at 90 min or even at 180 min are significant. The stabilization of DMO1 dependent on SA is more convincing (new Supplementary figure 2). The authors have however offered some explanations.

We appreciate the reviewer's comment. The modest SA-dependent effect on DMR6 degradation observed in the cell-free assay aligns with the simplified biochemical context of recombinant protein added to crude extracts. As mentioned in the revised Results section, the level of SA responsiveness is lower in this assay compared to in planta conditions, due to the lack of subcellular compartmentalization and the presence of residual endogenous SA. To prevent overinterpretation, we now clearly state in the text that the cell-free data serve as supportive qualitative evidence rather than a quantitative measurement: “*Accordingly, we interpret the cell-free degradation assay as qualitative support for SA-enhanced turnover rather than as a quantitative comparison*.”

Figure 1h presents data that catalytically inactive mutant degrades slower than wild type, but again changes are not really visible to the eye and seem extremely modest. Are they even significant? This is reconciled somewhat in the cell free degradation assays depicted in figure 1j and k.

We appreciate the reviewer’s concern and agree that the difference in degradation between wild-type DMR6 and the catalytic mutant seems modest in the transient expression assay. To better reflect this, we have moved this panel to the Supplementary Information (now Fig. S3e) and revised the text to present this result as supportive, rather than central, evidence. We now highlight that the cell-free degradation assays (Fig. 1j and now in revised Fig. S3) with the MG132 stabilization experiment in *N. benthamiana* together provide stronger and more consistent evidence that the catalytic mutant exhibits reduced turnover. We have also revised the figure legend and results section to clarify that the effect observed in the transient assay is subtle, which aligns with the low dynamic range of this system and the expectation that catalytic impairment influences DMR6 turnover alongside SA-dependent regulation. We hope these changes address the reviewer’s concern:

RESULTS: *“In the transient assay, the difference in turnover between wild-type DMR6 and the catalytic mutant was modest, as expected in this lower-dynamic-range system. We present this result in Supplementary Fig. 3e and highlight the clearer, more reproducible turnover differences seen in the cell-free degradation assay and in the MG132 stabilization experiment (Fig. h-j).”*

DISCUSSION: *“The subtle turnover difference observed for the catalytic mutant in the transient assay likely reflects the limited dynamic range of this system. The more robust effects observed in the cell-free degradation assay and MG132-dependent stabilization provide stronger support for reduced turnover of the catalytically inactive DMR6.”*

The authors later characterize the F-Box protein identified in their Turbo-ID experiment as interacting with DMR6 and call it DAF1. They verify interaction with DMR6 by way of co-immunoprecipitation and show stabilization of DMR6 in the daf1 mutant, suggesting that DAF1 promotes proteasomal turn-over, which is supported by the ratiometric assay. The authors continue to make a case for DMR6 accumulation in the daf1 mutant following SA exposure however Figure 4i, which is supposed to show this, is not clear to me (everything seems to be in the daf1 background, where is the comparison to Col-0?) and the quantitative differences are less than modest, although this is explained. Supplemental figure 9c shows strongly reduced DMR6 abundance in the daf1 background and the conclusion that this is due to "a self-limiting SA-DMR6 feedback loop that fine-tunes DMR6 abundance" is not sufficiently explained and may also be overinterpreted. Reduced DMR6 ubiquitylation is shown by way of a DMR6 pull-down and anti-Ub western, but again differences are hardly visible so the quantification is not conclusive suggesting that the phenomenon of DAF1 mediated DMR6 ubiquitylation is hardly relevant.

We thank the reviewer for these helpful comments and have revised the text to clarify the presentation and interpretation of the DAF1–DMR6 data. In Figure 4i, we focused on the SA response within the *daf1* background because endogenous DMR6 protein levels are extremely low in Col-0. Using the same membrane exposure for both genotypes bleaches out the endogenous DMR6 signal relative to *daf1*, making it difficult to measure protein levels accurately. Therefore, the direct Col-0 comparison is shown in Supplementary Fig. 9c. <u>We now explicitly state this in the Results and figure legend to prevent confusion.</u>

RESULTS: "*In this assay, we imaged daf1 only because endogenous DMR6 is extremely low compared to Col-0, and co-exposing both genotypes on the same membrane bleaches the endogenous DMR6 signal relative to Col-0, making accurate measurement of protein levels difficult. The corresponding Col-0 comparison is shown in Supplementary Fig. 9c, where each genotype could be imaged under appropriate exposure conditions. These side-by-side data confirm the expected difference in endogenous DMR6 abundance between the genotypes and are consistent with a model in which SA-induced transcription and post-translational turnover jointly fine-tune DMR6 levels.*"

LEGEND: *"Direct comparison of DMR6 levels in Col-0 and daf1 is shown separately in Supplementary Fig. 9c because co-exposure on the same membrane bleaches the endogenous DMR6 signal in daf1 relative to Col-0 and prevents accurate measurement."*

We also acknowledge that the endogenous changes in DMR6 abundance are modest. This low-amplitude behavior is expected for a homeostatic SA-catabolic enzyme whose steady-state level reflects the balance between SA-induced transcription and proteasomal turnover. We have softened the interpretation accordingly and now frame these measurements as supportive rather than primary mechanistic evidence. We also discuss that additional E3 ligases may contribute to DMR6 regulation, which could partly explain the nuanced phenotypes observed:

DISCUSSION*: "Although the endogenous DMR6 changes observed in daf1 are modest, such low-amplitude effects are expected for tightly regulated SA-catabolic enzymes. The complexity and partial redundancy of plant E3 ligases likely mean that DAF1 works together with additional*

UPS components to control DMR6 turnover, which may contribute to the nuanced phenotypes seen in certain assays."

Regarding the Ub pull-down, we agree that ubiquitylated DMR6 species are low in stoichiometry and inherently difficult to detect. We have clarified in the text and legend that the signal is transient and low in abundance, as is common for proteasome substrates, and we now describe the pull-down as consistent with, rather than definitive proof of, DAF1-mediated ubiquitylation. We further support this by citing published di-Gly proteomics datasets that identify DMR6 and DLO1 as ubiquitinated proteins, as well as our functional data showing DMR6 stabilization in *daf1* and SA-dependent turnover in the ratiometric assay.

The authors have quantified both SA and 2,5-DHBA levels and 2,5-DHBA is reduced in the daf1 mutant. This is counterintuitive at first, as an absence of DAF1 implies attenuated proteasomal degradation and increased DMR6 abundance, the authors reconcile this with a self-limiting regulatory feedback loop where DMR6 abundance is controlled both by SA induced transcriptional induction as well as DAF1 mediated, SA dependent proteolysis.

We thank the reviewer for this observation. The lower 2,5-DHBA levels in *daf1* may seem counterintuitive at first. We now clarify in the Discussion that DMR6 abundance depends on the combined effects of SA-induced transcription and SA-enhanced DAF1-mediated turnover, and that disrupting this turnover component changes the timing and level of DMR6 availability during SA fluctuations. This dynamic balance naturally leads to reduced 2,5-DHBA accumulation in daf1.

DISCUSSION: "*The lower 2,5-DHBA levels in daf1 may seem counterintuitive at first, but they align with the self-limiting structure of SA breakdown. SA triggers DMR6 transcription while also promoting DAF1-dependent proteasomal degradation; disrupting this degradation process alters when and how much DMR6 is available, leading to decreased 2,5-DHBA. The partial redundancy among plant E3 ligases likely adds more regulation, offering a natural explanation for the subtle metabolic behavior."*

The authors state that the daf1 mutant shows increased resistance to Pst DC3000 infection (less necrotic lesions) suggesting the protein is a negative regulator of immunity. At the same time DMR6 is also cited as a negative regulator, making it complex to reconcile the functional interaction between these two proteins in light of the papers primary narrative, that DAF1 leads to DMR6 ubiquitylation and proteasomal degradation.

[Response] We thank the reviewer for highlighting this point. We have now clarified in the manuscript how the DAF1–DMR6 relationship fits into the broader context of SA-dependent immunity. DMR6 acts as a negative regulator of immunity by catalyzing SA breakdown. In our model, DAF1 functions as an SA-responsive E3 ligase that promotes DMR6 degradation, thereby limiting the buildup of active DMR6 and allowing SA levels to increase appropriately during immune activation. Loss of DAF1 therefore decreases DMR6 degradation and shifts the

balance toward heightened immune responses, consistent with the phenotypes observed in *daf1* mutants and in previously described dmr6 mutants.

We have revised the Discussion to clarify the regulatory logic. We also emphasize that plant UPS pathways often include multiple partially redundant E3 ligases, which can result in subtle or context-dependent phenotypes when individual components are removed. This redundancy likely affects the complex relationship between DAF1 and DMR6 and explains why DMR6 levels, SA metabolites, and pathogen responses do not change in an “all-or-nothing” manner in daf1. These points are now more clearly articulated in the text.

RESULTS: *“DMR6 is a negative regulator of immunity, and DAF1 promotes its turnover. Therefore, loss of DAF1 is expected to shift the balance toward enhanced immune responses, consistent with the reduced disease symptoms observed in daf1.”*

DISCUSSION: “*At first glance, the relationship between DAF1 and DMR6 may seem complex because both influence immunity. However, DMR6 acts as a negative regulator by catalyzing the inactivation of SA, while DAF1 promotes DMR6 turnover in response to SA buildup. Loss of DAF1 decreases DMR6 turnover and boosts SA-dependent immunity, aligning with daf1 phenotypes. The modest, context-dependent effects are also consistent with the partial redundancy common to plant E3 ligase networks, suggesting that DAF1 functions alongside other UPS components to maintain DMR6 homeostasis.”*

In summary I think a plethora of experiments have been conducted yet the quantified phenomena are often very modest and some of the data remains conflicting or at least difficult to reconcile in a clear mechanistic model.

[Response] We appreciate the reviewer’s overall evaluation and agree that several of the quantitative effects observed in our study are small in magnitude. We have revised the Results and Discussion to clearly acknowledge this point and explain why modest, context-dependent changes are expected for endogenous SA-catabolic enzymes, which are tightly regulated to maintain homeostasis. To address the reviewer’s concern, we have significantly softened the language throughout the Results, ensuring that each conclusion matches the size of the underlying effect. We have also reorganized and clarified several figure panels, added more quantitative annotations, and included explanatory details emphasizing the consistency among biochemical, genetic, and metabolite-based evidence. This includes recognizing the likely role of additional E3 ligases in DMR6 turnover, which aligns with the partial redundancy common in plant UPS pathways and offers a natural explanation for the subtle behavior observed in some assays.

Reviewer #2

Remarks to the Author:

In this revised manuscript, the authors have addressed my previous comments and questions and have updated their model by incorporating a self-limiting SA-DMR6-DAF1 feedback loop. Within this loop, SA promotes DMR6 transcription while simultaneously enhancing DAF1-mediated proteasomal degradation of DMR6, thereby modulating cellular DMR6 abundance and consequently SA homeostasis. This model positions DAF1 as a central regulatory component that likely contributes positively to plant immunity. Consistent with this notion, the authors report that DAF1 overexpression induces SA-dependent cell death, whereas pathogen-induced cell death is attenuated in the daf1 T-DNA insertion mutant.

We appreciate the reviewer's positive feedback and the clear summary of our model. We are pleased that the revisions have enhanced the manuscript and clarified the regulatory logic of the SA–DMR6–DAF1 loop. Additionally, we have made further textual and figure clarifications in response to the reviewer's subsequent comments.

However, the disease susceptibility analysis presented for daf1 is not convincing. The bacterial growth data in Figure 4j appear incorrect. Following spray inoculation, Pst DC3000 typically proliferates by approximately 1000-fold in Col-0 plants after three days (Melotto et al., 2006, Cell 126:969-980), yet essentially no bacterial growth was observed in the presented results. To ensure reliable interpretation, the authors should perform leaf infiltration to bypass potential confounding effects of stomatal immunity. It should be noted that the virulence of Pst DC3000 can decline following repeated subculture or prolonged storage.

We thank the reviewer for raising this point. We agree that the spray-inoculation dataset showed a low dynamic range and significantly lower overall *Pst* DC3000 proliferation than what is usually observed under optimal conditions. Since spray infection results can be heavily influenced by environmental factors that affect bacterial entry and virulence, we have moved these CFU measurements to the Supplementary Information and now present them as supportive, but preliminary, observations rather than as mechanistic evidence.

To prevent overinterpretation, we have also softened the language in the Results and Discussion sections and instead highlighted the more consistent SA-dependent cell-death phenotypes observed in *daf1* and in DAF1 overexpression lines. These assays are less affected by entry-related variability and more accurately reflect immune activation outcomes.

Finally, in line with the reviewer's suggestion, we now clearly state that infiltration-based pathogen assays are an important next step for future research aimed at fully understanding the contribution of the SA–DMR6–DAF1 module to bacterial growth restriction.

RESULTS: "*In a spray inoculation assay, the growth of Pst DC3000 was significantly lower in daf1 mutants compared to wild-type Col-0 at 3 days post-spray (Supplementary Fig. 10a, b). Furthermore, trypan blue staining revealed less necrosis on Pst DC3000-infected daf1 leaves relative to Col-0 (Supplementary Fig. 10c, d).*"

DISCUSSION*: “Because spray-inoculation outcomes are highly sensitive to environmental conditions that influence stomatal entry and pathogen virulence, future infiltration-based assays will be important for fully defining the contribution of DAF1 to bacterial growth restriction.”*

Furthermore, to substantiate the proposed self-limiting feedback regulation, the authors are encouraged to compare SA- and pathogen-induced DMR6 transcriptional kinetics in Col-0 and daf1, along with dynamic measurements of pathogen-induced SA accumulation in both genotypes. These experiments are technically feasible for the authors and would be expected to provide strong mechanistic evidence supporting the revised model.

We thank the reviewer for this insightful suggestion. We agree that detailed SA- and pathogen-induced transcriptional and time-course metabolic measurement would further refine the quantitative model of the proposed self-limiting SA–DMR6–DAF1 circuit. However, these experiments require new plant growth and multi-point pathogen time courses that extend beyond the current revision cycle. In line with the editor’s guidance, we have focused on strengthening the clarity and interpretation of the existing data instead of attempting to incorporate new infection-based kinetics. Importantly, the regulatory node we describe functions mainly at the post-translational level: SA enhances DAF1-mediated proteasomal degradation of DMR6. Although DMR6 transcription is induced by SA, our results show that the critical control point is the regulation of DMR6 protein stability, not transcriptional changes. Therefore, transcriptional or SA-accumulation kinetics are not necessary for our mechanistic conclusions. To address the reviewer’s concern, we also softened our interpretation of pathogen-related outcomes and revised the language to avoid overstating that DAF1 directly controls *Pst* DC3000 susceptibility. We now describe these responses as SA-dependent immune-associated processes rather than direct pathogen susceptibility, which aligns better with our data. Additionally, we refined the final model figure to illustrate that DAF1 contributes to DMR6 turnover within a broader, partially redundant ubiquitin-ligase network. We also clarified that DAF1’s role in modulating SA homeostasis is not viewed as a standalone factor determining pathogen growth. Finally, we included further explanation that the kinetic analyses suggested by the reviewer represent promising future work aimed at quantitatively understanding how SA-induced transcription and DAF1-mediated proteolysis intersect during pathogen challenge. We hope these clarifications fully address the reviewer’s concerns.

Under DISCUSSION we also added: “*Because the regulatory node characterized here acts mainly at the post-translational level, the main mechanistic conclusions do not depend on transcriptional or SA-accumulation kinetics. We therefore interpret the daf1 phenotypes as SA-dependent immune responses rather than direct measures of pathogen susceptibility.*
Future infection-based transcriptional and metabolic time-course experiments will be crucial for fully understanding how SA-induced transcription and DAF1-mediated turnover coordinate during pathogen challenge.

Reviewer #3

Authors have fully addressed the comments from the first round of review. I congratulate the authors on their excellent work.

We appreciate the reviewer's positive feedback and are pleased that the revisions have satisfactorily addressed the previous concerns.

Reviewer #4

I co-reviewed this manuscript with one of the reviewers who provided the listed reports. This is part of the Nature Communications initiative to facilitate training in peer review and to provide appropriate recognition for Early Career Researchers who co-review manuscripts

We appreciate the reviewer's input in evaluating our manuscript.

Point-by-point response to reviewers

Reviewer #1 (Remarks to the Author):

I thank the authors for the time they have taken in addressing my previous comments. I am beginning to appreciate their line of argumentation that the plethora of experiments show a potential feedback loop regulating DMR6 levels, composed on the one hand of SA induced synthesis and on the other by DAF1 mediated DMR6 ubiquitylation and subsequent proteasomal degradation and that this accounts for the nuanced effects observed in many experiments. Also, interesting data on pathogen susceptibility and immunity dependent on DAF1 are shown, characterizing the protein as well as the structural models that support SA dependent conformational shift and changes in stability. The story is the placed into the larger context of protein interaction networks in an infection scenario although I would have liked to see this in the daf1 background to connect the last section with the sections before it.
I think at this point no further experiments will be necessary as many have been performed already producing interesting data that, although in some cases is seemingly contradictory, does support the hypothesis of the feedback loop. However, I think it goes too far to state that it has been unequivocally shown. Therefore, I think the authors should edit the manuscript one more time focusing on making things clearer and phrasing some of their conclusions more carefully. Below are a summary of and a few points that arose in the section reporting the daf1 experiments, which is the last one I have any major issues with.

Lines 436 to 440: DAF1 leads to DMR6 and DLO1 degradation. Fig4 g and h show less protein in Wt compared to daf1.

[Response] We thank the reviewer for their close attention to the data and its significance in the overall context of the manuscript. Figure 4g & h depict recombinantly purified DMR6 and DLO1 incubated with native Arabidopsis extracts from either wild-type or *daf1*. As the reviewer states, relative protein abundance is reduced (shorter half-life/faster degradation) in the wild-type condition compared to *daf1*, which is consistent with DAF1 promoting DMR6/DLO1 degradation, given that slower proteasomal degradation is observed when the proteins are incubated with *daf1* knockout extract.
<u>To address this point and make this clearer</u>, in panels Fig. 4g and 4h we replaced “WT” and “*daf1*” with “WT extract” and “*daf1* extract” to emphasize that the western blots depict recombinant DMR6/DLO1 incubated with wild-type or *daf1* extract, not endogenous DMR6 protein levels in the wild-type and *daf1* backgrounds (that is shown in Fig. 4i, where endogenous DMR6 levels are relatively elevated in *daf1* plants. We have also adjusted the wording in the accompanying results section to further emphasize that recombinant protein is visualized in this figure.

Lines 440 to 444: In planta situation. DMR6 is very low abundant in daf1 compared to Wt background although it accumulates to slightly higher levels in the mutant than the wild type when proteasome inhibitor is applied. Shouldn’t the DAF1 knockout be sufficient to attenuate degradation and lead to DMR6 accumulation? This seems to be the conclusion that can be drawn from the cell free degradation assays above. It hardly seems to be the case in planta as levels are more or less the same when comparing 0, 1 and 2 hours of SA treatment alone without proteasome inhibitor. Addition of MG132

enhances the effect and it seems to me the authors are stating that due to the additional application of SA, DMR6 synthesis induced by SA and degradation mediated by DAF1 jointly modulate DMR6 levels, the later introduced regulatory feedback loop.

[**Response**] We agree with the reviewer that endogenous DMR6 protein levels are low in the daf1 background relative to wild type, and that MG132 treatment results in only modest accumulation. Our data indicate that although DAF1 promotes DMR6 proteasomal degradation, degradation is not fully abolished in its absence, as demonstrated by residual turnover in cell-free assays and reduced but detectable ubiquitination in *daf1* (Supplementary Fig. 9d).
These observations suggest that DAF1 is not the sole regulator of DMR6 stability. In addition, DMR6 abundance is strongly influenced by SA-dependent transcriptional induction (Zhang et al., 2017). In *daf1*, reduced 2,5-DHBA levels indicate altered SA turnover (Supplementary Fig. 9g), which likely attenuates SA-mediated DMR6 transcription. Thus, steady-state DMR6 levels reflect the combined effects of transcriptional regulation and proteasomal degradation rather than a simple one-to-one relationship between DAF1 loss and substrate accumulation.
To better clarify and address this point, we have revised the Results and Discussion to emphasize this dual control rather than implying a simple one-to-one relationship between DAF1 loss and DMR6 accumulation.

Lines 444 to 450: Why is DMR6 so low abundant in the daf1 background? Looking at Suppl fig 9c I can see that DMR6 is degraded 1 hour after DMSO treatment to 0.75 without the application of SA. Also note, comparing to Figure 1d, degradation of endogenous DMR6 is similar with application of SA (0.68). In line 450 the authors state the differences in DMR6 abundance between genotypes supports the feedback loop model but this seems very speculative at this point.

[**Response**] We agree that the low steady-state abundance of DMR6 in the daf1 background requires clarification. As noted above, our data indicate that DAF1 promotes DMR6 proteasomal degradation but is not the sole determinant of its turnover. Residual degradation and ubiquitination are still detectable in the absence of DAF1, suggesting additional regulatory inputs. Importantly, in Supplementary Fig. 9c (now Supplementary Fig. 9d), all seedlings were treated with SA. DMSO serves only as the solvent control for MG132. We acknowledge that this was not sufficiently clear in the figure panel and have revised the labeling to explicitly indicate SA treatment in all conditions. Therefore, the apparent reduction to ~0.75 at 1 hour reflects SA-associated dynamics rather than basal degradation in the absence of SA.
With respect to the genotype comparison, steady-state DMR6 abundance likely reflects the combined effects of SA-dependent transcriptional induction and proteasomal turnover. In daf1, reduced 2,5-DHBA levels indicate altered SA metabolism, which may attenuate SA-driven DMR6 transcription. Thus, differences in DMR6 protein abundance between genotypes are consistent with coordinated transcriptional and post-translational regulation rather than a simple one-to-one relationship between DAF1 loss and substrate accumulation.
To address the reviewer's concern, we have revised the Results and Discussion to more carefully frame this as a regulatory model supported by the data, rather than as a definitively established feedback loop.

Lines 451 to 457: DMR6 ubiquitylation is reduced in daf1 background showing aberrant DMR6 protein abundance regulation in the genotype. Does DAF1 mediate DMR6 ubiquitylation and so it accumulates in the knockout because less is ubiquitylated and hence targeted for degradation? What happens endogenously when you treat the daf1 only with MG132 but not SA analogous to figure 4i?

We thank the reviewer for raising this important point. Indeed, our data indicate that DAF1 contributes to DMR6 ubiquitination, as ubiquitinated DMR6 species are somewhat reduced in *daf1*. However, ubiquitination and degradation are not fully abolished in the mutant, indicating that DAF1 may not be the sole regulator of DMR6 turnover. Experiments shown in Fig. 4i and Supplementary Fig. 9 were performed under SA treatment; thus, the time course reflects DMR6 dynamics in the presence of exogenous SA rather than basal conditions. We note that DMR6 protein levels are already markedly lower in *daf1* at time 0, prior to monitoring degradation kinetics in all experiments. This indicates that steady-state abundance differs between genotypes before additional turnover is assessed. As discussed above, DMR6 levels likely reflect the combined effects of SA-dependent transcriptional induction and proteasomal turnover.
To address the reviewer's concern, we have revised the Results section to clarify the experimental context and to avoid overinterpreting genotype differences as definitive evidence of a fully resolved feedback mechanism.

Lines 458 to 463: No DMR6 in npr1. Has it been shown that NPR1 is a transcriptional activator of DMR6?

We thank the reviewer for this important question. Previous studies demonstrate that SA-induced DMR6 expression is attenuated in the *npr1-1* background (PMID: 18248595), indicating that NPR1 contributes to *DMR6* transcription under immune conditions. In addition, TGA2, a transcription factor that interacts with NPR1, has been shown to bind the *DMR6* promoter (PMID: 33037144). While the precise mechanistic requirement for NPR1–TGA interaction in DMR6 activation has not been fully resolved, the available evidence supports a functional link between NPR1-dependent signaling and *DMR6* transcription. We have revised the Results section to clarify this relationship and to avoid implying a direct, fully characterized transcriptional mechanism.

Lines 467 to 469: DAF1 promotes DMR6 degradation and DMR 6 hydroxylates SA to produce 2,5-DHBA. Why are 2,5-DHBA levels lower when there is no DAF1 as in daf1? The authors state DMR6 activity is reduced in daf1. Where have they shown this? I see no activity assays, enzyme kinetics or anything of the sort in the daf1 background, if I haven't missed it. I see the explanation in lines 660 to 666 of the discussion but it is speculative.

We thank the reviewer for this important clarification. We did not perform enzymatic activity assays in the *daf1* background and do not intend to imply altered intrinsic catalytic activity of DMR6. Rather, the reduced 2,5-DHBA levels observed in daf1 correlate with the reduced steady-state abundance of DMR6 protein in this genotype. To avoid overinterpretation, we have revised the Results section to remove references to altered "DMR6 activity" and instead state that decreased 2,5-DHBA levels are consistent with reduced DMR6 protein abundance. The corresponding

Discussion text has also been adjusted to eliminate speculative language regarding catalytic regulation.

Lines 473 to 478: I can appreciate the authors hypothesis of the concurrent SA induced transcriptional regulation concurrent with the SA induced, DAF1 mediated DMR6 turn-over controlling SA levels to a point, in other words I see hints at this in the presented data, but I believe it is still too speculative. The whole section and conclusions need to be formatted much cautiously also in the discussion.

We thank the reviewer for this important comment. We agree that the proposed regulatory framework should be presented with appropriate caution. Our data show that SA promotes DMR6 transcription, that DAF1 contributes to DMR6 proteasomal turnover, and that disruption of DAF1 alters DMR6 abundance and SA inactivation product levels. Together, these findings support a model consistent with coordinated transcriptional and post-translational regulation of DMR6, rather than a definitively established feedback mechanism. Accordingly, we have revised the Results and Discussion sections to frame this explicitly as a regulatory model supported by convergent genetic, biochemical, metabolic, and structural evidence. Language throughout has been adjusted to avoid overstatement and to clearly distinguish direct observations from conceptual interpretation. We have also refined the schematic model (Figure 6), using dashed arrows to denote proposed regulatory relationships.
Importantly, as detailed below, revised infection assays demonstrate increased Pst proliferation in daf1 relative to Col-0, aligning the genetic phenotype with DAF1's role in DMR6 turnover. We believe these additional data strengthen the proposed regulatory relationship between DAF1 and DMR6 while maintaining a cautious interpretation of the broader model.

Lines 487 to 495: I think this whole section should be deleted, it adds no relevant information and interrupts the story which continues on line 496. Loss of DAF1 in daf1 leads to increased immunity.

We thank the reviewer for this helpful suggestion. We agree that the placement of the AF3 modeling and C-terminal helix docking analysis interrupted the flow of the pathogen susceptibility results. To improve clarity and narrative coherence, we have relocated this section to follow directly after the DMR6–DAF1 co-immunoprecipitation results (now line 321). In this revised position, the modeling data serve to support the physical interaction between DMR6 and DAF1 and provide structural context for that interaction, without interrupting the phenotypic analysis.

The authors state in lines 484 to 486 that DMR6 is a negative regulator of immunity and that DAF1 promotes its turnover (do you mean degradation Fig 4g), so wouldn't loss of DAF1 promote accumulation of the negative regulator thereby suppressing immunity? Please explain this. In line, DAF1 overexpression is reported to lead to a type of auto-immune phenotype, i.e. leaf necrosis which is alleviated by DMR6 co-expression or depletion of SA by NahG co-expression, suggesting dependence of auto-immunity on SA and regulation by both DMR6 and DAF1. These are interesting findings but should be clarified.

We thank the reviewer for this important conceptual question. The relationship between DAF1-mediated DMR6 turnover and immune output depends on the net balance between SA-dependent transcriptional induction and proteasomal regulation of DMR6. As noted above, steady-state DMR6 abundance in daf1 does not simply reflect loss of degradation but instead results from combined transcriptional and post-translational influences.

To directly address the immune phenotype, we repeated the *Pst* infection assays using syringe infiltration and a fresh bacterial stock to ensure robust colonization. Under these conditions, daf1 mutants consistently exhibited increased *Pst* proliferation relative to Col-0 at 3 dpi (new Fig. 4j and new Suppl. Fig. 10). These revised data align the genetic phenotype with DAF1's contribution to DMR6 turnover and clarify the apparent discrepancy in the earlier dataset.

With respect to DAF1 overexpression, transient expression in *N. benthamiana* induced leaf necrosis, which was alleviated by DMR6 co-expression or SA depletion via NahG. These findings indicate that DAF1 activity influences SA homeostasis and that both insufficient and excessive DAF1 activity can perturb immune balance.

We have revised the Results and Discussion to clarify this regulatory interplay and to avoid implying a simple linear relationship between DAF1 loss and DMR6 accumulation.

Section on ASK1 Turbo-ID after Pst DC3000 inoculation: The experiment itself is interesting but as I already remarked in an earlier review, DMR6 and DAF1 are dropped from the narrative at this point. What about the same TurboID experiment in the daf1 background as it seems it has an effect on plant immunity? Maybe for future studies.

We thank the reviewer for this insightful comment. The ASK1–TurboID experiment was designed to characterize SCF-associated proximity networks during *Pst* infection following our observation that DMR6 steady-state levels change under these conditions. Although this approach did not directly assess DAF1-specific recruitment dynamics, it confirmed consistent association of DMR6 and DLO1 with SCF complexes and identified additional SA-related components potentially regulated through this pathway. We agree that performing proximity labeling in the *daf1* background during pathogen challenge would provide valuable mechanistic insight into DAF1-dependent network remodeling. While such experiments were beyond the scope of the present study, we have now added a brief statement in the Discussion highlighting this as a logical direction for future investigation.

Discussion

Generally, no issues, but I think it goes too far to say the feedback loop has been clearly shown. Discussion should be phrased more carefully.

We thank the reviewer for this comment. We agree that the Discussion should carefully distinguish between direct experimental findings and conceptual interpretation. While our data support a coordinated regulatory relationship between SA, DMR6, and DAF1, we have revised the Discussion to frame this explicitly as a regulatory model rather than a definitively established feedback loop.

Throughout the manuscript, language has been refined to avoid overstatement and to clearly separate observation from inference. In addition, we have revised the final schematic (Figure 6) to replace “self-limiting feedback loop” with “regulatory circuit” and to denote proposed regulatory relationships using dashed arrows. We believe these revisions more accurately reflect the scope of the data while preserving the conceptual framework supported by our findings.

Reviewer #2 (Remarks to the Author):

It is surprising that the authors did not attempt to properly verify the defense phenotype of the daf1 mutant, given that this is a critical piece of evidence required to support their biochemical conclusions. Without accurate phenotypic characterization, the interpretation of the biochemical data remains uncertain. The daf1 mutation could plausibly alter pathogen-induced SA accumulation and thereby increase or decrease resistance to bacterial pathogens. These possibilities would fundamentally influence how the biochemical results should be understood.

Simply relocating the flawed resistance-assay data to the supplementary information does not resolve the issue, as the underlying experiment was not performed correctly based the pathogen growth on the wild-type plants and the results as presented are misleading. While the biochemical analyses are interesting, rigorous mutant phenotyping is essential in this case. I would request that the editor extend the revision deadline so the authors can properly determine the true defense phenotype of the daf1 mutant.

We thank the reviewer for emphasizing the importance of rigorous phenotypic validation. We agree that accurate characterization of the *daf1* defense phenotype is essential for interpreting the biochemical findings. Upon reevaluation, we recognized that the initial spray inoculation assay yielded uniformly low bacterial proliferation in wild-type plants, limiting its interpretive value. To address this directly, we repeated the infection experiments using syringe infiltration (rather than spray inoculation) and a freshly prepared *Pst* DC3000 stock to ensure robust colonization and reproducible bacterial growth.

Under these conditions, we consistently observed significantly increased *Pst* proliferation in *daf1* compared to Col-0 at 3 dpi, approximately an order of magnitude higher bacterial load (new Figure 4j and Suppl. Fig. 10). These results establish that *daf1* exhibits enhanced susceptibility to *Pst*, thereby aligning the genetic phenotype with DAF1’s role in modulating DMR6 turnover.

We appreciate the reviewer’s insistence on this point, as the revised phenotypic analysis strengthens the mechanistic coherence of the study.
